# Identification of selective *N*-pyridinsulfonyl indole based thiosemicarbazone derivatives as potential antiproliferative agents against lung cancer cells

**DOI:** 10.1039/d6ra02731h

**Published:** 2026-07-02

**Authors:** Zahra Batool, Halil Şenol, Rushba Saman Masood, Tugce Salduz, Fatma Betul Yoladi, Fahri Akbas, Feyzi Sinan Tokali, Norah A. Albekairi, Asif Rasool, Zahid Shafiq, Abdulrahman Alshammari

**Affiliations:** a Institute of Chemical Sciences, Bahauddin Zakariya University Multan 60800 Pakistan zahidshafiq@bzu.edu.pk; b Department of Pharmaceutical Chemistry, Faculty of Pharmacy, Bezmialem Vakif University Fatih Istanbul 34093 Turkiye; c Department of Chemistry, Quaid i Azam University Islamabad 45320 Pakistan; d Department of Molecular Biology and Genetics, Faculty of Engineering and Natural Sciences, Üsküdar University 34662 Istanbul Turkiye; e Department of Pharmaceutical Toxicology, Faculty of Pharmacy, Atatürk University 25240 Erzurum Turkiye; f Department of Medical Biology, Faculty of Medicine, Bezmialem Vakif University Fatih Istanbul 34093 Turkiye; g Department of Material and Material Processing Technologies, Kars Vocational School, Kafkas University 36100 Kars Turkiye; h Department of Pharmacology and Toxicology, College of Pharmacy, King Saud University Post Box 2455 Riyadh 11451 Saudi Arabia; i School of Chemistry and Chemical Engineering, Nanjing University Nanjing 210093 China

## Abstract

Lung cancer remains one of the leading causes of cancer-related mortality worldwide and necessitates the development of novel targeted agents. In this study, a series of new thiosemicarbazone derivatives (5a–r) were synthesized and evaluated for their anticancer potential through combined *in vitro* and *in silico* approaches. Cytotoxic activities were assessed against lung adenocarcinoma cells (A549) and normal bronchial epithelial cells (BEAS-2B). Among the tested compounds, 5p emerged as the most potent derivative, exhibiting an IC_50_ value of 3.41 µM against A549 cells, superior to sorafenib (IC_50_ = 4.02). Importantly, 5p demonstrated a remarkably high selectivity index (SI = 17.5), substantially exceeding that of sorafenib (SI = 5.5), indicating preferential cytotoxicity toward cancer cells. Apoptosis assays revealed that 5p significantly induced apoptotic cell death in A549 cells, supporting that its antiproliferative activity is mediated, at least in part, through apoptosis induction. Molecular docking and MM-GBSA binding free energy analyses against EGFR, VEGFR-1, and VEGFR-2 further supported the experimental findings. Compound 5p exhibited strong docking scores (−11.452, −13.864, and −11.487 kcal mol^−1^ for EGFR, VEGFR-1, and VEGFR-2, respectively) and the most favorable binding free energies (Δ*G*_bind = −91.95, −87.05, and −80.53 kcal mol^−1^), outperforming reference inhibitors gefitinib and sorafenib in several targets. Molecular dynamics simulations (250 ns) confirmed the stability of the 5p–kinase complexes based on RMSD analysis. *In silico* ADME analysis revealed that the synthesized compounds generally possess favorable drug-likeness. The combined *in vitro* cytotoxicity, high selectivity index, and strong *in silico* kinase binding profiles identify compound 5p as a promising lead candidate for further development in lung cancer therapy.

## Abstract 2

Lung cancer remains one of the leading causes of cancer-related mortality worldwide and necessitates the development of novel targeted agents.

## Graphical abstract

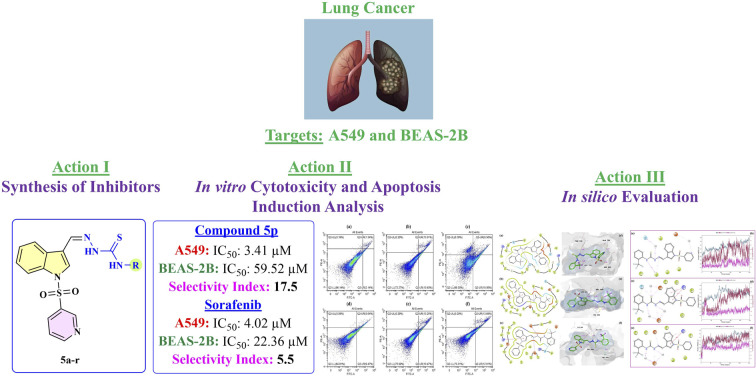

## 1 Introduction

Lung cancer remains one of the most prevalent and deadly malignancies worldwide. According to recent global cancer statistics, lung cancer accounts for a substantial proportion of both newly diagnosed cancer cases and cancer-related deaths each year. Despite advances in diagnostic strategies and therapeutic interventions, the overall survival rate remains unsatisfactory, particularly in advanced-stage disease.^1,2^ Non-small cell lung cancer (NSCLC) constitutes approximately 85% of all lung cancer cases, with adenocarcinoma being the most common histological subtype.^3^ The A549 cell line, derived from human lung adenocarcinoma, is widely used as an *in vitro* model for evaluating potential therapeutic agents due to its relevance to NSCLC biology and responsiveness to targeted treatments.^4^

Chemotherapy remains a cornerstone in the management of lung cancer, particularly in patients with advanced or metastatic disease who are not eligible for surgical intervention. Although chemotherapeutic agents can prolong survival and alleviate symptoms, their clinical utility is often constrained by significant systemic toxicity, including myelosuppression, nephrotoxicity, and neurotoxicity. In addition, the lack of selectivity toward malignant cells frequently results in damage to rapidly dividing normal tissues, thereby limiting the maximum tolerated dose. The emergence of intrinsic or acquired resistance further diminishes long-term therapeutic efficacy, contributing to disease relapse and poor prognosis. These limitations highlight the continued need for novel small-molecule candidates with improved selectivity and reduced adverse effects.^4,5^

Among the molecular pathways implicated in lung cancer progression, receptor tyrosine kinases play a central role in regulating cell proliferation, survival, migration, and angiogenesis. In particular, the epidermal growth factor receptor (EGFR) and vascular endothelial growth factor receptors (VEGFRs) have been extensively associated with tumor growth and metastatic potential in non-small cell lung cancer. Aberrant activation or overexpression of EGFR promotes uncontrolled cellular proliferation and resistance to apoptosis, while signaling mediated by VEGFR-1 and VEGFR-2 contributes to tumor-associated angiogenesis and vascular remodeling.^6,7^ Pharmacological inhibition of these kinases has demonstrated clinical benefit in selected patient populations, supporting their relevance as therapeutic targets. Nevertheless, the development of resistance and the variability of patient responses indicate that the discovery of structurally diverse small molecules capable of interacting with these signaling pathways remains of considerable interest.^8^ In this context, compounds exhibiting antiproliferative activity may warrant further mechanistic exploration with respect to their potential interactions with key oncogenic kinases.

**Scheme 1:**
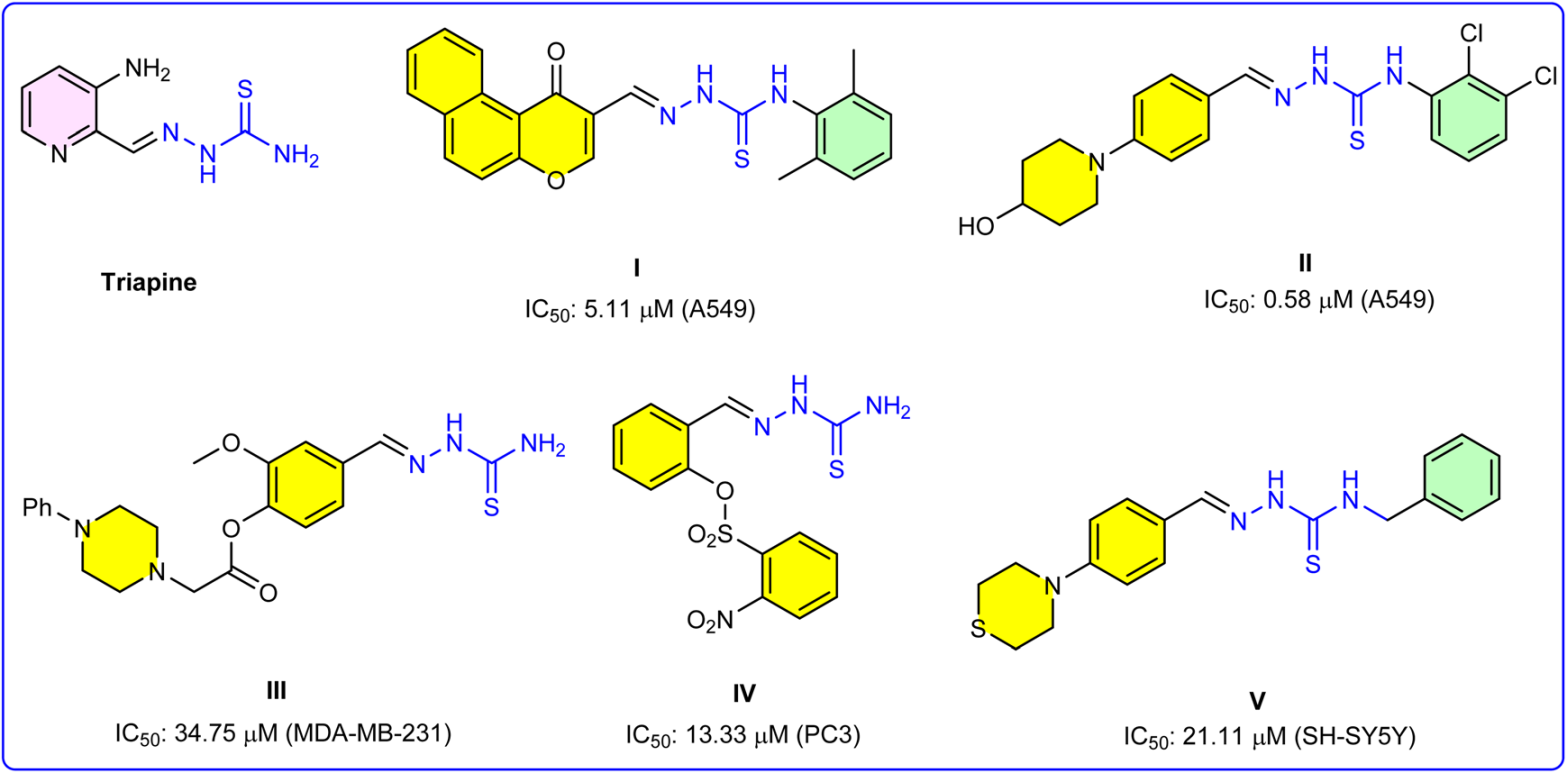
Thiosemicarbazone derivatives with potential anticancer properties.

Thiosemicarbazone derivatives have attracted sustained attention in anticancer drug discovery due to their versatile chemical reactivity and broad spectrum of biological activities. These compounds are known to interact with multiple intracellular targets, partly attributed to their metal-chelating capacity and ability to modulate redox homeostasis.^9^ Several thiosemicarbazone-based molecules have demonstrated pronounced antiproliferative effects across different cancer cell lines, highlighting their potential as scaffolds for the development of novel chemotherapeutic agents (I–V) (Scheme 1).^10–14^ One of the most extensively studied representatives of this class is Triapine, which has been evaluated in clinical settings for various malignancies, particularly in combination regimens. In addition, structural modification at the N-terminal region has been shown to significantly influence their cytotoxic potency and selectivity profiles. Given these multifaceted biological properties, thiosemicarbazone-containing hybrids continue to represent promising candidates for the design of structurally novel anticancer agents with improved pharmacological characteristics.

In the present study, a hybrid molecular design strategy was adopted to integrate multiple pharmacologically relevant structural motifs into a single framework. The selection of the thiosemicarbazone core was primarily motivated by its well-documented antiproliferative potential and its structural versatility, which allows fine-tuning of biological activity through N-terminal substitutions.^15,16^ The presence of azomethine and thioamide functionalities enables diverse polar interactions and coordination capabilities, features that are often associated with enhanced binding affinity toward biologically relevant targets. The indole nucleus was incorporated as a privileged scaffold frequently encountered in bioactive natural products and synthetic anticancer agents. Owing to its planar aromatic structure and favorable electronic properties, the indole ring is capable of engaging in polar and hydrophobic interactions within enzyme active sites and receptor binding pockets. Moreover, indole-based derivatives have repeatedly demonstrated antiproliferative and pro-apoptotic activities in lung cancer models, supporting their inclusion as a key pharmacophoric element in the present design. To further modulate the physicochemical and biological properties of the scaffold, the indole moiety was functionalized with a pyridine-sulfonyl fragment *via* sulfonamide linkage formation. The introduction of the sulfonamide group was envisioned to enhance hydrogen-bonding capacity, improve solubility, and provide additional interaction points with polar amino acid residues. The pyridine ring, on the other hand, contributes an electron-deficient heteroaromatic system that may strengthen binding interactions within kinase-associated binding cavities through complementary electrostatic and π-related contacts. Collectively, this structural integration was designed to generate hybrid molecules with balanced lipophilicity, structural rigidity, and interaction diversity, potentially translating into improved antiproliferative profiles.

Based on the aforementioned considerations, the present study was designed to develop a novel series of indole-based sulfonamide thiosemicarbazone derivatives through a rational hybridization strategy. The antiproliferative activities of the compounds were evaluated against the human lung adenocarcinoma A549 cell line, while their cytotoxic effects on BEAS-2B cells were assessed to estimate selectivity toward malignant cells. The most active compound was further investigated for its apoptosis-inducing potential in order to gain preliminary insight into its mechanism of action. In addition, *in silico* molecular modeling studies were performed to explore the possible interactions of the selected compound with EGFR and VEGFR-1 and 2, thereby providing a structural perspective on its observed biological activity.

## 2 Results and discussion

### 2.1 Chemistry

**Scheme 2:**
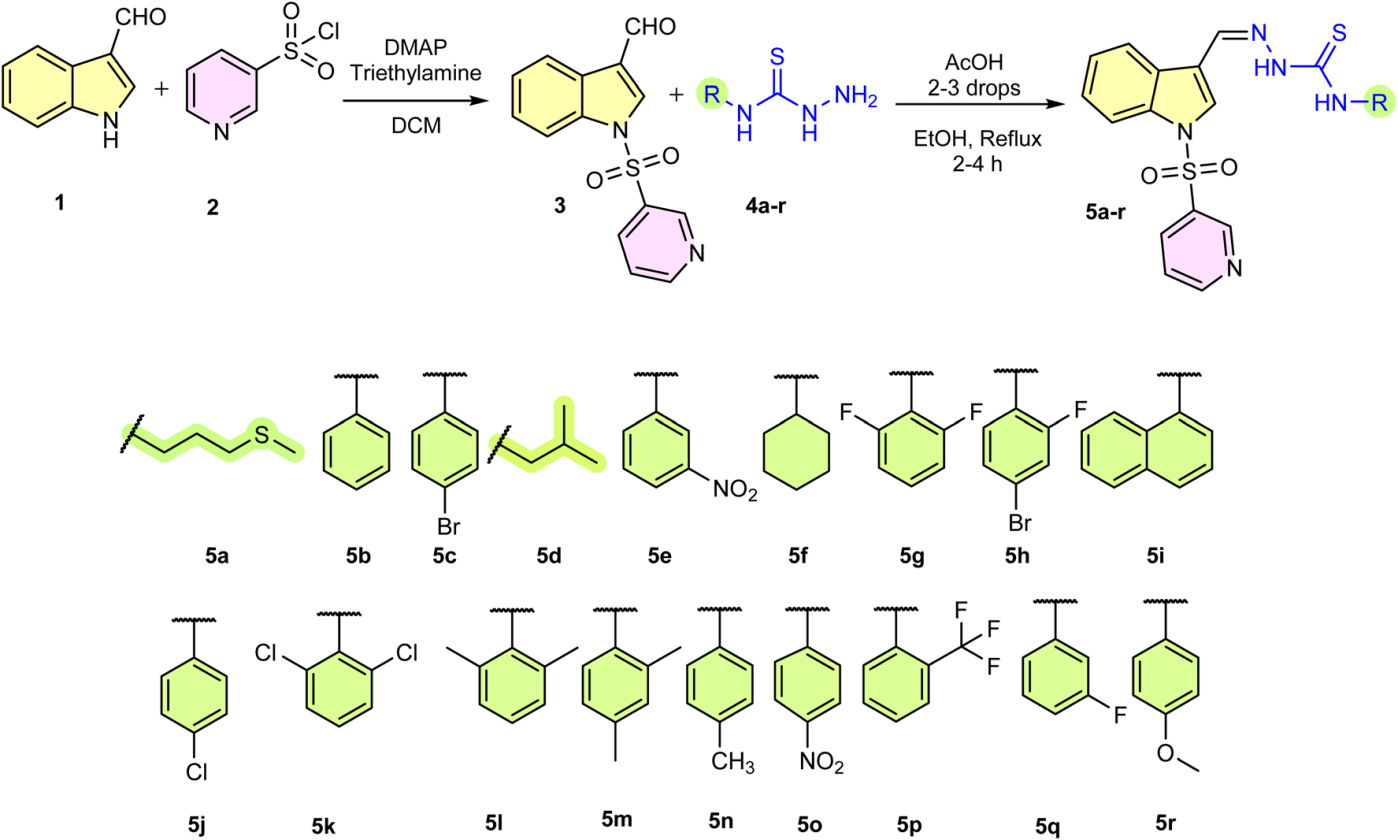
The synthetic route for preparation of compounds 5a–r.

The formation of *N*-pyridylsulfonylindole-based thiosemicarbazones (5a–r) took place in two steps. Firstly, the preparation of N-substituted indole is done by substituting commercially available Indole 3-carbaldehyde (1) with pyridyl sulfonyl chloride (2) using DMAP and triethylamine as the base and DCM as solvent. The targeted *N*-pyridylsulfonylindole-3-carbaldehyde (3) was obtained after extraction with sodium bicarbonate solution as white precipitates in an appreciable yield of 92% (Scheme 2).

In the next step, *N*-pyridylsulfonylindole-3-carbaldehyde intermediate (3) was transformed into a series of thiosemicarbazones (5a–r) by employing appropriate *N*-substituted thiosemicarbazides (4a–r) in an equimolar ratio. Thiosemicarbazone derivatives (5a–r) were synthesized by stirring 3 and the corresponding *N*-substituted thiosemicarbazide (4a–r) (1.1 eq.) in ethanol for 3–5 hours under reflux conditions (Scheme 2). The thiosemicarbazones (5a–r) were obtained in 85–95% yield.

The formation of *N*-substituted indole intermediate (3) and thiosemicarbazones (5a–r) was confirmed by their ^1^H and ^13^C NMR. In the thiosemicarbazone intermediates, the functional motif –NH–C(=S)–NH– gives rise to characteristic signals associated with a thiocarbonyl group. The presence of two singlets for NH protons at 12.24–11.54 ppm and 10.20–9.23 ppm and a singlet at 8.49–8.35 ppm for the azomethine proton in the 1H NMR spectra of corresponding thiosemicarbazone (5a–r) suggests the successful condensation of thiosemicarbazide at the aldehydic position of indole. In the ^13^C NMR, peaks at 177.92–175.81 and 162.67–155.43 ppm can be assigned to the C=S and C=N, respectively.^17–20^

### 2.2. *In vitro* cytotoxicity results and structure–activity relationship (SAR)

The antiproliferative activities of compounds 5a–5r were evaluated against the human lung adenocarcinoma A549 cell line, while cytotoxicity toward BEAS-2B cells was assessed to determine selectivity. The obtained IC_50_ values revealed that several derivatives exhibited pronounced growth inhibitory effects in the low micromolar range (Table 1).

**Table 1:** The cytotoxicity and selectivity results of compounds 3, 5a–r, and reference sorafenib

Compounds	IC_50_ [µM]		Selectivity index
	A549	BEAS2B	BEAS2B/A549
3	39.53 ± 2.35	52.63 ± 2.78	1.3
5a	18.35 ± 2.65	50.02 ± 2.30	2.7
5b	14.62 ± 3.30	58.96 ± 2.14	4.0
5c	7.75 ± 2.07	49.11 ± 2.73	6.3
5d	26.75 ± 2.12	53.49 ± 3.01	2.0
5e	6.11 ± 2.07	27.00 ± 2.87	4.4
5f	19.84 ± 2.14	54.66 ± 2.18	2.7
5g	6.85 ± 1.85	54.70 ± 3.65	7.9
5h	8.05 ± 2.24	50.43 ± 2.45	6.2
5i	13.62 ± 3.25	43.02 ± 3.90	3.1
5j	9.65 ± 1.15	59.08 ± 2.79	6.1
5k	8.67 ± 1.95	60.24 ± 2.09	6.9
5l	18.55 ± 2.72	51.92 ± 2.58	2.8
5m	20.64 ± 2.69	45.14 ± 2.24	2.2
5n	17.53 ± 2.92	41.62 ± 3.63	2.3
5o	9.06 ± 2.36	36.24 ± 3.16	4.0
5p	3.41 ± 0.61	59.52 ± 3.72	17.5
5q	5.12 ± 0.82	50.41 ± 3.10	9.8
5r	17.94 ± 2.79	57.97 ± 3.95	3.2
Sorafenib	4.02 ± 0.19	22.36 ± 2.72	5.5

Among the tested compounds, derivative 5p emerged as the most potent antiproliferative agent, with an IC_50_ value of 3.41 ± 0.61 µM, surpassing the reference drug sorafenib (IC_50_ = 4.02 ± 0.19 µM) in terms of activity against A549 cells. Importantly, 5p displayed markedly reduced toxicity toward BEAS-2B cells (IC_50_ = 59.52 ± 3.72 µM), resulting in a remarkable selectivity index (SI = 17.5), substantially higher than that of sorafenib (SI = 5.5). This finding indicates a favorable therapeutic window and selective cytotoxic action toward malignant cells.

Compounds 5q (IC_50_ = 5.12 µM, SI = 9.8) and 5g (IC_50_ = 6.85 µM, SI = 7.9) also demonstrated strong antiproliferative activity accompanied by high selectivity indices, suggesting that specific structural modifications significantly enhance cancer cell selectivity. Similarly, derivatives 5c, 5h, 5j, and 5k exhibited IC_50_ values below 10 µM and selectivity indices above 6, indicating a consistent trend of potent yet preferential cytotoxicity toward A549 cells.

In contrast, compounds such as 5d, 5f, 5l, 5m, and 5n showed comparatively weaker antiproliferative activity (IC_50_ > 17 µM) and modest selectivity (SI = 2–3), suggesting that certain substituent patterns may negatively influence either target engagement or cellular uptake.

To investigate the contribution of the thiosemicarbazone moiety to the antiproliferative activity, the cytotoxic effect compound 3 was also evaluated. It exhibited moderate activity against A549 cells (IC_50_ = 39.53 µM) with a low selectivity index of 1.3. In contrast, all thiosemicarbazone derivatives displayed improved antiproliferative activities, with IC_50_ values ranging from 3.41 to 26.75 µM and selectivity indices of 2.0–17.5. These findings indicate that incorporation of the thiosemicarbazone pharmacophore substantially enhances both the antiproliferative potency and selectivity of the *N*-pyridylsulfonyl indole scaffold.

The majority of the synthesized thiosemicarbazone derivatives demonstrated selective cytotoxic activity, with several compounds outperforming the reference drug in terms of both potency and selectivity. The particularly high selectivity index observed for compound 5p highlights its potential as a promising lead candidate for further mechanistic and optimization studies.

#### 2.2.1 SAR analysis

The antiproliferative activity of the synthesized thiosemicarbazone derivatives was markedly influenced by the nature and position of the substituent on the terminal *N*-aryl moiety, indicating a strong SAR.

The design of the thiosemicarbazone derivatives was based on systematic variation of the *N*-substituent to explore the influence of electronic properties, steric demand, and lipophilicity on antiproliferative activity. In this context, aliphatic, simple aromatic, halogenated, electron-withdrawing, and electron-donating substituents were intentionally introduced to establish a clear SAR and to identify the key structural features governing cytotoxic potency.

A comparison between aliphatic and aromatic substituents clearly demonstrates that the presence of an aromatic ring is crucial for enhanced activity. Aliphatic derivatives such as 5a (3-methylthiopropyl, IC_50_ = 18.35 µM), 5d (iso-butyl, IC_50_ = 26.75 µM), and 5f (cyclohexyl, IC_50_ = 19.84 µM) exhibited relatively weak antiproliferative effects against A549 cells. This reduction in activity may be attributed to the absence of a π-conjugated aromatic system, which limits the ability to engage in π–π stacking or hydrophobic interactions within the biological target environment. In addition, the increased conformational flexibility of aliphatic chains may reduce binding pre-organization, leading to less efficient target engagement.

Introduction of a phenyl ring led to a noticeable improvement in activity, as observed for compound 5b (phenyl, IC_50_ = 14.62 µM), indicating that aromaticity is a key pharmacophoric requirement. However, simple aromatic substitution alone was not sufficient to achieve optimal potency. Extension of the aromatic system, as in the 1-naphthyl derivative 5i (IC_50_ = 13.62 µM), did not result in a significant further improvement, suggesting that excessive steric bulk and increased hydrophobicity may adversely affect either binding fit or cellular permeability, thereby limiting overall activity.

To further refine the SAR, various electron-withdrawing and electron-donating substituents were introduced at different positions of the aromatic ring to systematically modulate electronic density, steric environment, and lipophilicity. A marked enhancement in activity was observed for halogen-substituted derivatives. Compounds 5c (4-bromophenyl, IC_50_ = 7.75 µM), 5j (4-chlorophenyl, IC_50_ = 9.65 µM), 5k (2,6-dichlorophenyl, IC_50_ = 8.67 µM), 5g (2,6-difluorophenyl, IC_50_ = 6.85 µM), and 5q (3-fluorophenyl, IC_50_ = 5.12 µM) showed significantly improved antiproliferative activity compared to the unsubstituted phenyl analogue 5b. This improvement may be rationalized by the electron-withdrawing nature of halogens, which can stabilize key electronic interactions within the thiosemicarbazone framework, as well as increased lipophilicity that may facilitate membrane permeation. Additionally, halogen atoms can participate in halogen bonding and enhance hydrophobic interactions within potential binding pockets. The particularly strong activity of the CF_3_-substituted derivative 5p (IC_50_ = 3.41 µM), which surpassed the reference drug sorafenib (IC_50_ = 4.02 µM), further supports the importance of a strong electron-withdrawing effect combined with an optimal steric and lipophilic balance.

Nitro-substituted derivatives also exhibited notable cytotoxic activity, particularly compound 5e (3-nitrophenyl, IC_50_ = 6.11 µM), whereas the para-nitro analogue 5o (IC_50_ = 9.06 µM) showed comparatively lower potency. This difference suggests that positional effects play a significant role in modulating electronic distribution and spatial orientation of the pharmacophore. The strong electron-withdrawing character of the nitro group likely contributes to activity enhancement; however, its influence appears to be highly dependent on substitution pattern and resulting molecular geometry.

In contrast, derivatives bearing electron-donating substituents generally exhibited reduced antiproliferative activity. Compounds such as 5l (2,6-dimethylphenyl, IC_50_ = 18.55 µM), 5m (2,4-dimethylphenyl, IC_50_ = 20.64 µM), 5n (4-methylphenyl, IC_50_ = 17.53 µM), and 5r (4-methoxyphenyl, IC_50_ = 17.94 µM) were significantly less potent than electron-withdrawing analogues. This decrease in activity may be attributed to increased electron density on the aromatic system, which could weaken favorable electronic interactions within the thiosemicarbazone core and reduce optimal binding affinity. Furthermore, steric effects, particularly in *ortho*-substituted derivatives such as 5l, may introduce conformational constraints that negatively impact binding orientation and overall biological activity.

As a result, the SAR findings clearly indicate that electron-withdrawing substituents, particularly halogens and trifluoromethyl groups, substantially enhance antiproliferative activity, whereas electron-donating groups are associated with reduced potency. In addition, *meta* substitution appears to provide a favorable balance between electronic influence and steric compatibility. Among all derivatives, compound 5p emerged as the most promising candidate, combining superior potency with outstanding selectivity, thereby representing a valuable lead structure for further mechanistic and optimization studies.

### 2.3 Apoptosis induction analysis

**Fig. 1:**
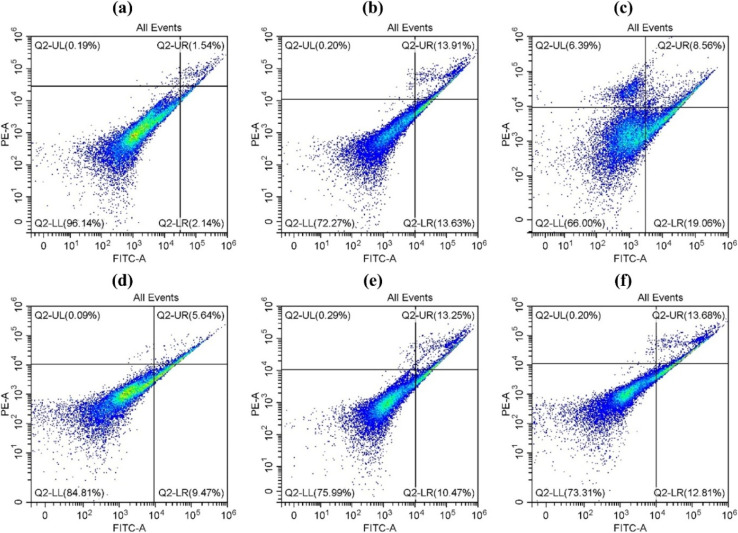
Representative apoptosis profiles of A549 cells treated with compound 5p and sorafenib at increasing concentrations. Panels (a–c) show compound 5p at 1, 5, and 10 µM, respectively, while panels (d–f) show sorafenib at 1, 5, and 10 µM, respectively. Apoptotic populations were analysed as healthy/viable, early apoptosis, late apoptosis, and necrosis. The untreated control group exhibited viable: 95.52%, early apoptosis: 0.90%, late apoptosis: 5.73%, and necrosis: 0.86%.

To determine whether the observed antiproliferative activity was associated with programmed cell death, the most potent derivative, compound 5p, was subjected to apoptosis analysis and compared with the reference drug sorafenib. Apoptotic cell populations were quantified following treatment at 1, 5, and 10 µM concentrations by assessing the distribution of viable, early apoptotic, late apoptotic, and necrotic cells. Representative apoptosis profiles are shown in Fig. 1.

Compound 5p demonstrated a clear concentration-dependent shift from viable to apoptotic cell populations. At 1 µM, 5p induced only a slight increase in early apoptotic cells (2.14%) while maintaining a predominantly viable population (96.14%), indicating limited cytotoxic stress at low concentration. Under the same conditions, sorafenib produced a comparatively stronger apoptotic response.

At 5 µM, compound 5p triggered a pronounced apoptotic response, reaching a total apoptosis rate (early + late) of 27.54%, slightly exceeding that observed for sorafenib (23.72%). Importantly, necrotic cell fractions remained minimal at this concentration for both treatments, suggesting that growth inhibition was primarily mediated through apoptosis rather than nonspecific cytotoxicity.

At 10 µM, 5p further increased early apoptotic cells (19.06%); however, a noticeable rise in necrotic cells (6.39%) was also detected. In contrast, sorafenib maintained a low necrotic fraction (0.20%) with a relatively stable late apoptotic distribution. The increased necrosis observed at the highest concentration of 5p may reflect concentration-dependent cellular stress beyond apoptotic thresholds.

As a result, compound 5p exhibited a strong apoptosis-inducing capacity in A549 cells, particularly at intermediate concentration, where apoptotic induction was comparable to or slightly higher than that of sorafenib while maintaining limited necrotic involvement. These findings suggest that the antiproliferative effect of 5p is at least partially mediated through activation of apoptotic pathways.

### 2.4 Molecular docking

To gain preliminary insights into the potential molecular interactions of the synthesized compounds, molecular docking studies were performed. It should be emphasized that the docking analysis was not intended to establish a definitive mechanism of action. Rather, this computational approach was employed to explore possible interactions with selected tyrosine kinase targets that are commonly implicated in the proliferation and survival of lung cancer cells.

Given that dysregulation of receptor tyrosine kinases plays a central role in the pathogenesis and progression of non-small cell lung cancer, particularly in A549 cells, the epidermal growth factor receptor (EGFR) and vascular endothelial growth factor receptors (VEGFR-1 and 2) were selected as representative targets. EGFR is frequently overexpressed or aberrantly activated in lung cancer and contributes to uncontrolled cell proliferation and survival signalling.^21^ Similarly, VEGFR-1 and 2 are key mediators of angiogenesis and tumor progression, and their inhibition represents a validated therapeutic strategy in lung cancer management.^22–24^

Accordingly, the binding affinities and interaction profiles of the synthesized derivatives toward EGFR, VEGFR-1 and 2 were evaluated through molecular docking simulations. In the analyses, Induced Fit Docking (IFD) scores and MM-GBSA binding free energies were considered together, and the results were compared with reference inhibitors. Gefitinib was used as the reference for EGFR, and Sorafenib was used for VEGFR-1 and 2. The calculated docking scores and key molecular interactions are summarized in Table 2.

**Table 2:** IFD scores and MM-GBSA Δ*G* binding free energies of the compounds and references against EGFR, VEGFR-1 and 2

Compounds	IFD docking scores (kcal mol^−1^)			MM-GBSA Δ*G* bind (kcal mol^−1^)		
	EGFR	VEGFR-1	VEGFR-2	EGFR	VEGFR-1	VEGFR-2
5a	−10.891	−11.365	−9.649	−68.56	−74.90	−62.84
5b	−10.119	−11.412	−9.923	−73.54	−56.13	−60.33
5c	−9.667	−12.554	−10.900	−82.60	−75.69	−68.29
5d	−10.043	−12.710	−8.352	−69.02	−58.83	−58.68
5e	−9.999	−13.422	−9.789	−80.35	−82.22	−56.79
5f	−10.754	−10.759	−9.864	−75.79	−70.30	−59.77
5g	−9.812	−11.618	−9.711	−65.38	−58.80	−54.83
5h	−9.079	−11.315	−10.675	−66.69	−71.97	−64.25
5i	−9.783	−13.531	−11.544	−79.25	−75.34	−76.05
5j	−9.494	−13.850	−10.410	−81.36	−68.25	−67.42
5k	−9.719	−13.594	−9.952	−69.84	−77.46	−75.07
5l	−11.009	−13.511	−11.337	−84.86	−79.35	−74.80
5m	−11.120	−10.513	−10.643	−77.23	−74.38	−71.86
5n	−9.116	−14.153	−11.234	−54.57	−75.68	−78.67
5o	−9.148	−8.982	−10.326	−72.17	−73.07	−73.28
5p	−11.452	−13.864	−11.487	−91.95	−87.05	−80.53
5q	−10.372	−11.028	−9.298	−71.07	−71.46	−59.63
5r	−9.090	−9.082	−10.357	−82.40	−63.32	−67.91
Gefitinib	−9.872	—	—	−76.21	—	—
Sorafenib	—	−11.998	−13.648	—	−83.04	−80.98

In terms of EGFR, the reference compound gefitinib showed an IFD score of −9.872 kcal mol^−1^ and an MM-GBSA value of −76.21 kcal mol^−1^. Several derivatives within the series exhibited lower binding scores than these values. Compound 5p, in particular, stands out with an IFD score of −11.452 kcal mol^−1^ and an MM-GBSA value of −91.95 kcal mol^−1^. These values suggest that 5p may have the potential to form a more stable complex in the EGFR binding pocket than the reference inhibitor.

Examining the VEGFR-1 results, sorafenib showed IFD of −11.998 kcal mol^−1^ and MM-GBSA of −83.04 kcal mol^−1^. Many compounds in the series yielded similar or lower IFD scores than the reference. In this context, 5p exhibited a more advantageous profile than the reference in terms of both docking and binding free energy, with an IFD score of −13.864 kcal mol^−1^ and a MM-GBSA value of −87.05 kcal mol^−1^.

When evaluated for VEGFR-2, sorafenib showed the lowest value at the docking stage with an IFD score of −13.648 kcal mol^−1^. However, considering the MM-GBSA results, 5p's value of −80.53 kcal mol^−1^ is quite close to sorafenib's value of −80.98 kcal mol^−1^, indicating comparable binding stability.

It is noteworthy that compound 5p, which showed the strongest cytotoxic and apoptotic effect in *in vitro* studies, also exhibited the lowest or comparable binding energies to the reference in computational analyses across all targets. This suggests that the cytotoxic activity observed in the A549 cell line may potentially be related to multiple tyrosine kinase interactions. This parallelism suggests a consistent relationship between the biological activity of 5p and its computational binding tendencies; however, a direct mechanism is not proposed here. The aim here is to predict the interactions of compounds with tyrosine kinases, and molecular docking results need to be supported by *in vitro* inhibition experiments to establish a definitive mechanism.

**Fig. 2:**
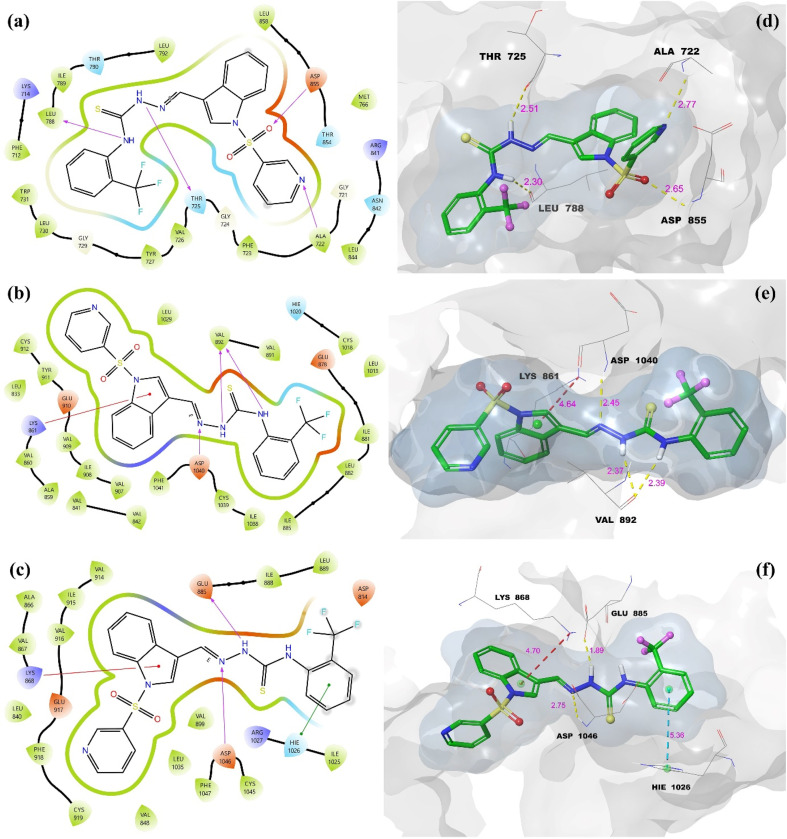
Molecular docking 2D and 3D ligand–protein interactions of 5p with EGFR, VEGFR-1 and 2. 2D 5p–EGFR complex (a), 2D 5p–VEGFR-1 complex (b), 2D 5p–VEGFR-2 complex (c), 3D 5p–EGFR complex (d), 3D 5p–VEGFR-1 complex (e), 3D 5p–VEGFR-2 complex (f).

Considering the *in vitro* cytotoxicity results along with IFD and MM-GBSA scores, compound 5p, which emerged as the strongest candidate, was examined in detail in terms of its molecular interactions with EGFR, VEGFR-1 and 2, and its binding modes are presented in Fig. 2. This analysis aims not to propose a mechanism, but rather to evaluate at the structural level how 5p is positioned in the active sites of these tyrosine kinases and which critical residues it interacts with.

When the 5p–EGFR complex (Fig. 2a and d) is examined, it is seen that the compound is stabilized by multiple hydrogen bond interactions within the active site. Two nitrogen atoms in the thiourea group form two separate hydrogen bonds with residues Leu788 and Thr725 at distances of 2.30 and 2.51 Å, respectively. These interactions are important anchor points supporting the ligand's positioning close to the kinase hinge site.^24^ Furthermore, the hydrogen bond between sulfonamide oxygen and Asp855 at a distance of 2.65 Å is noteworthy. Asp855 is one of the residues located in the catalytic region of EGFR and considered critical for activity; therefore, this interaction may significantly contribute to the stability of the complex.^25^ The hydrogen bond formed by the pyridine nitrogen with Ala722 at a distance of 2.77 Å reinforces the correct orientation of the ligand in the binding pocket.^26^ The coexistence of multiple and short-range hydrogen bonds suggests a binding pattern for 5p that is consistent with the low MM-GBSA values obtained for EGFR.

When the 5p–VEGFR-1 complex is evaluated (Fig. 2b and e), it is again seen that the thiourea group plays a decisive role. Thiourea nitrogen atoms form two hydrogen bonds with Val892 at distances of 2.37 and 2.39 Å, ensuring the ligand's secure attachment to the active site. In addition, the hydrogen bond formed by the azomethine nitrogen with Asp1040 at a distance of 2.45 Å is particularly important in terms of electrostatic stability around the catalytic site; this interaction is noteworthy considering the functional role of aspartate residues in kinase activity.^27^ Furthermore, the cation–π interaction formed by the indole ring with Lys861 at a distance of 4.64 Å provides electrostatic contribution beyond classical hydrogen bonds, increasing complex stability. Such interactions between the positively charged side chains of Lys residues and aromatic systems are considered complementary factors that increase binding strength, especially in kinase inhibitors.^28^

The 5p–VEGFR-2 complex (Fig. 2c and f) exhibits a rich profile in terms of both hydrogen bonds and aromatic interactions. The hydrogen bond formed between thiourea nitrogen and Glu885 at a relatively short distance of 1.89 Å indicates a strong and directional interaction. This distance suggests that hydrogen bonding may significantly contribute to the binding stability. The hydrogen bond observed between azomethine nitrogen and Asp1046 at a distance of 2.75 Å also forms an additional anchor point near the catalytic site. Furthermore, the cation–π interaction between the indole ring and Lys868 at a distance of 4.70 Å shows parallelism with a similar interaction observed in VEGFR-1. In addition, the π–π stacking interaction of the trifluoromethyl-substituted phenyl ring with His1026 at 5.36 Å demonstrates that hydrophobic and aromatic contributions are also involved in the binding stability.^28^ This versatile interaction network is compatible with the robust MM-GBSA values calculated for VEGFR-2 by 5p.

As a result, 5p appears to form repetitive hydrogen bonding motifs with critical residues near the catalytic or hinge region, particularly *via* thiourea and azomethine functional groups, in all three kinase targets. In addition, cation–π interactions between the indole ring and Lys residues, and π–π interactions between aromatic rings, play a complementary role in binding stability. This multiple and complementary interaction pattern provides a consistent binding model that can explain, at a structural level, why 5p stands out in both *in vitro* biological activity and computational binding analyses.

Beyond their *in vitro* antiproliferative activity, the physiological relevance of the synthesized thiosemicarbazone derivatives can be further supported by their predicted interactions with key cancer-related molecular targets. The docking studies, particularly for the most active compound 5p, suggest favorable binding modes within multiple enzyme active sites implicated in tumor progression. These interactions indicate that the observed cytotoxic effects may be associated with the modulation of biologically relevant signaling pathways rather than nonspecific toxicity. Considering the known role of thiosemicarbazone scaffolds in metal chelation and redox imbalance in cancer cells, these findings further support their potential to interfere with essential physiological processes involved in cancer cell survival and proliferation. These results provide a preliminary but meaningful link between the observed *in vitro* activity and possible *in vivo* pharmacological relevance.

### 2.5 Molecular dynamics (MD) simulations

**Fig. 3:**
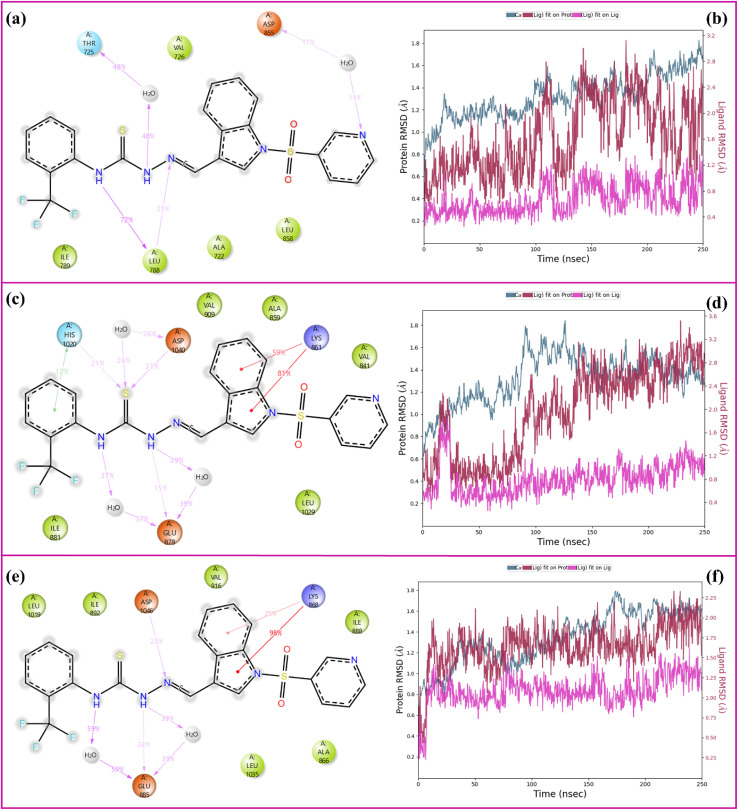
The 250 ns MD simulation analysis of 5p–EGFR, 5p–VEGFR-1 and 2 complexes. (a) 2D key ligand–protein interactions of 5p–EGFR complex, (b) RMSD of ligand and protein atoms of 5p–EGFR complex, (c) 2D key ligand–protein interactions of 5p–VEGFR-1 complex, (d) RMSD of ligand and protein atoms of 5p–VEGFR-1 complex, (e) 2D key ligand–protein interactions of 5p–VEGFR-2 complex, (f) RMSD of ligand and protein atoms of 5p–VEGFR-2 complex.

To evaluate the time-dependent stability of complexes formed by 5p, which stands out as the compound with the strongest binding profile in docking analyses, with target proteins, 250 ns MD simulations were performed. This analysis aims to go beyond static docking results and reveal whether ligand–protein interactions are maintained in a dynamic environment and the temporal fluctuations of complex stability.^29^ This approach provides a complementary analysis for evaluating whether the computationally predicted strong binding tendency of 5p is sustainable under dynamic conditions. The conformational behavior of 5p complexes formed with EGFR, VEGFR-1 and 2 was investigated through basic stability parameters and interaction continuity, and the results obtained are presented in Fig. 3.

When the 5p–EGFR complex (Fig. 3a) was examined, it was observed that thiourea and azomethine nitrogen atoms formed two separate hydrogen bonds with Leu788, and these interactions were maintained at 72% and 21%, respectively, during the simulation. Considering the proximity of Leu788 to the hinge region, the hydrogen bond, which showed a particularly high continuity of 72%, contributed to the stable retention of the ligand in the binding pocket. The other thiourea nitrogen atom formed a 48% water–bridged hydrogen bond with Thr725, while the pyridine nitrogen atom exhibited an 11% water-mediated interaction with Asp855. The fact that Asp855 is a critical residue in the catalytic region suggests that this contact, although at a low percentage, may be functionally significant. In the RMSD analysis, average values of 1.2 Å were obtained for protein Cα atoms, 1.6 Å for the protein-fitted ligand, and 0.8 Å for ligand internal RMSD (Fig. 3b).

The 5p–VEGFR-1 complex exhibits a more dense and diverse interaction network (Fig. 3c). Thiourea nitrogen atoms support the ligand's position in the active site by forming two water-bridged hydrogen bonds (39% and 37%) with Glu878, and an additional direct hydrogen bond (15%). It is noteworthy that thiourea sulfur forms 21% hydrogen bonds with His1020 and exhibits 31% direct and 26% water-bridged hydrogen bond interactions with Asp1040. The repeated contacts, particularly with Asp1040, suggest a stable anchoring mechanism around the catalytic site, consistent with the interactions observed during the docking phase. Furthermore, the indole ring forms two cation–π interactions with Lys861 at rates of 81% and 59%, respectively, providing a strong electrostatic contribution to complex stability; these high persistence percentages indicate that the aromatic system–Lys interaction is maintained even under dynamic conditions. RMSD values were calculated as 1.4 Å for protein Cα, 2.0 Å for the ligand fitted to the protein, and 0.8 Å for ligand internal RMSD (Fig. 3d).

The 5p–VEGFR-2 complex exhibits a stable profile in terms of both hydrogen bonds and cation–π interactions (Fig. 3e). Thiourea nitrogen atoms formed a strong interaction network in the active site with Glu885, consisting of two water-bridged bonds (59% and 39%) and one direct hydrogen bond (24%). The fact that Glu885 is one of the residues playing a significant role in kinase activity makes these high persistence rates structurally meaningful. Azomethine nitrogen formed 23% hydrogen bonds with Asp1046, thus providing an additional stabilization point near the catalytic site. Furthermore, the indole ring exhibited two cation–π interactions with Lys868 at 98% and 25%, particularly noteworthy for its high persistence value of 98%; this indicates that the ligand is strongly held in the binding pocket of the aromatic system. In RMSD analysis, values of 1.2 Å were obtained for protein Cα atoms, 1.75 Å for the protein-fitted ligand, and 1.25 Å for the ligand internal RMSD (Fig. 3f).

As a result, the MD results reveal that 5p largely maintains contact with critical residues identified during the docking phase, even under dynamic conditions. In particular, acidic residues near the hinge/catalytic site (Asp and Glu), cation–π interactions with Lys residues, and the repeating hydrogen bonding motifs of the thiourea group present a common and consistent binding pattern for the three targets. The low range of RMSD values in all complexes supports the formation of stable complexes in terms of both protein backbone and ligand conformation.

### 2.6 ADME predictions

Early evaluation of pharmacokinetic behavior plays a critical role in the drug development process, since a large proportion of bioactive candidates fail at later stages due to suboptimal ADME (absorption, distribution, metabolism, and excretion) characteristics. In this context, computational ADME prediction has emerged as a widely accepted strategy for assessing drug-likeness, gastrointestinal uptake, and membrane permeability of newly designed compounds. Such *in silico* approaches enable a rapid and reliable estimation of whether the physicochemical profiles of candidate molecules are compatible with the acceptable pharmacokinetic space.^30,31^ In the present work, the ADME characteristics of the synthesized compounds were evaluated using calculated molecular descriptors, and the corresponding results for compounds 5a–r are summarized in Table 3.

**Table 3:** ADME prediction results of the compounds 5a–r^a^

Comp	Ro5	Ro3	%HOA	QPPCaco	QPPMDCK	QP log BB	QP log Po/w	QP log *S*	aHB	dHB	MW
5a	0	0	93	475	989	−1.268	3.144	−5.543	11	2	447.59
5b	0	0	91	412	379	−1.295	2.923	−5.447	10	2	435.52
5c	1	1	81	409	997	−1.144	3.465	−6.244	10	2	514.41
5d	0	0	92	558	627	−1.105	2.742	−4.987	10	2	415.53
5e	0	0	70	50	38	−2.512	2.203	−5.549	11	2	480.52
5f	0	1	95	548	665	−1.079	3.173	−5.771	10	2	441.57
5g	0	1	95	480	1178	−1.023	3.360	−5.93	10	2	471.50
5h	1	1	83	454	1810	−0.988	3.701	−6.477	10	2	532.40
5i	0	1	100	579	647	−1.098	3.910	−6.338	10	2	485.58
5j	0	1	94	409	926	−1.152	3.391	−6.139	10	2	469.96
5k	1	1	86	558	2284	−0.851	3.823	−6.402	10	2	504.41
5l	0	1	96	518	442	−1.166	3.418	−5.843	10	2	463.57
5m	0	1	96	521	490	−1.205	3.542	−6.274	10	2	463.57
5n	0	1	93	414	382	−1.33	3.213	−5.969	10	2	449.54
5o	0	0	70	49	38	−2.521	2.208	−5.575	11	2	480.52
5p	1	1	86	572	1971	−0.877	3.869	−6.435	10	2	503.52
5q	0	1	92	411	683	−1.192	3.150	−5.792	10	2	453.51
5r	0	0	91	422	392	−1.353	2.994	−5.531	11	2	465.54

a MW 130 to 725 (molecular weight); dHB 0 to 6 (H-bond donors); aHB 2 to 20 (H-bond acceptors); QP log Po/w −2 to 6.5 (octanol/water partition coefficient); QP log *S* −6.5 to 0.5 (aqueous solubility, log *S*); QPPCaco <25 poor, >500 great (intestinal permeability); QP log BB −3 to 1.2 (brain/blood partition); QPPMDCK <25 poor, >500 great (BBB permeability); % HOA >80 high, <25 poor (oral absorption); Ro5 ≤4 (Lipinski); Ro3 ≤3 (Jorgensen).

The ADMET profile of compounds 5a–r indicates favorable drug-like properties across the series. Most derivatives complied with Lipinski's rule of five with either no or only a single violation, suggesting good overall drug-likeness. Similarly, Jorgensen's rule of three parameters were largely within acceptable ranges, supporting the pharmacokinetic suitability of the compounds.

The predicted intestinal permeability (QPPCaco) values were generally high to very high for most compounds, with many exceeding 400 and several surpassing 500, indicating strong potential for gastrointestinal absorption. Consistently, the % HOA values were predominantly high (mostly above 90%), further supporting favorable oral absorption profiles across the series.

In contrast, QP log BB values ranged from approximately −0.85 to −2.52, suggesting limited blood–brain barrier penetration, which may be advantageous for anticancer agents intended for peripheral action and reduced central nervous system exposure.

In terms of physicochemical properties, QP log Po/w values (2.2–3.9) fall within the optimal range for drug-like lipophilicity, while QP log *S* values (−4.9 to −6.4) indicate moderate to low aqueous solubility, which is commonly observed for structurally similar hydrophobic scaffolds.

Compounds such as 5p, 5k, 5h, and 5g stand out with simultaneously high Caco-2/MDCK permeability and high % HOA values, suggesting that these derivatives represent the most promising candidates in terms of overall ADMET performance within the series.

## 3 Conclusion

In this study, a novel series of thiosemicarbazone-based derivatives was synthesized and evaluated for anticancer activity against A549 lung cancer cells. The cytotoxicity screening revealed that several compounds exhibited meaningful antiproliferative effects, with compound 5p emerging as the most potent derivative, displaying an IC_50_ value of 3.41 µM. Importantly, 5p demonstrated a favorable selectivity profile toward cancer cells compared to the non-cancerous cell line, with a selectivity index of 17.5, indicating preferential cytotoxicity. Apoptosis analysis further confirmed that 5p induced concentration-dependent apoptotic cell death rather than nonspecific toxicity. At 5 µM, total apoptosis reached 27.54%, exceeding that of the reference drug under the same conditions, supporting a controlled antiproliferative effect.

Computational studies performed against EGFR, VEGFR-1 and 2 revealed that 5p consistently displayed the most favorable binding profile within the series. It showed superior or comparable IFD docking scores and MM-GBSA binding energies relative to the reference inhibitors, accompanied by stable hydrogen-bonding networks with key hinge and catalytic-site residues. Molecular dynamics simulations further confirmed the structural stability of these complexes, with low RMSD deviations throughout the simulation period.

The ADME profiling indicated that the majority of the synthesized thiosemicarbazone derivatives exhibit physicochemical and pharmacokinetic properties consistent with drug-like space. Several compounds demonstrated a balanced combination of high permeability and acceptable lipophilicity, highlighting their promise as potential lead candidates for further development.

Although the present findings highlight 5p as a promising lead candidate, this study is limited to *in vitro* biological assays and *in silico* computational analyses. The proposed interactions with tyrosine kinases are based solely on docking and molecular dynamics simulations and do not constitute direct evidence of kinase inhibition. Therefore, specific *in vitro* enzymatic tyrosine kinase inhibition assays are required to experimentally validate the predicted binding profiles. In addition, comprehensive *in vivo* studies are essential to evaluate pharmacokinetic behavior, systemic toxicity, and therapeutic efficacy. These further investigations will be necessary to clarify the precise mechanism of action and to determine the true translational potential of compound 5p.

## 4 Materials and methods

### 4.1 Chemistry

In order to generate thiosemicarbazones based on pyridin-3-ylsulfonyl indole, the following solvents and chemicals were purchased from Merck and utilized as such: petroleum ether, glacial acetic acid, DMAP, triethylamine, ethyl acetate, methanol, ethanol, and DCM. Silica gel plates with aluminum backs were used to track the reaction's progress and completion. With a Bruker Ascend 600 MHz NMR spectrometer, ^1^H and ^13^C NMR spectra were obtained in DMSO-*d*_6_ at 25 °C (600 MHz for ^1^H and 151 MHz for ^13^C). Coupling constants (*J*) were displayed in Hertz (Hz) and NMR spectra were displayed as chemical shifts (ppm) to illustrate signal multiplicity. High-resolution mass spectrometry (HRMS) analyses were carried out on a Thermo Fisher Scientific Q Exactive™ Hybrid Quadrupole-Orbitrap™ mass spectrometer.

#### 4.1.1 Synthesis of compound 3

Indole-3-carbaldehyde (1) (2 mmol, 0.29 g) was combined with a solution of triethylamine (2.6 mmol, 363 µL), DMAP (2 mmol, 0.245 g), and 3-pyridylsulfonyl chloride (2) (2 mmol, 0.35 g) in DCM (15 mL) under an argon atmosphere. At room temperature, the reaction mixture was agitated for 12 hours. The reaction mixture was treated with a saturated sodium bicarbonate solution. DCM was then used to extract the reaction mixture. A 1 : 4 ethyl acetate and hexane solution was used in flash chromatography to purify the residue of *N*-pyridylsulfonylindole-3-carbaldehyde (3) that was obtained.^20^

##### 4.1.1.1 *N*-pyridylsulfonylindole-3-carbaldehyde (3)

White solid, yield: 92%. ^1^H NMR (600 MHz, DMSO-*d*_6_) *δ* 10.08 (1H, s), 9.32 (1H, dd, *J* = 2.5, 0.8 Hz), 8.53 (1H, ddd, *J* = 8.2, 2.5, 1.5 Hz), 8.12 (1H, dt, *J* = 7.7, 1.0 Hz), 8.02 (1H, dt, *J* = 8.5, 0.9 Hz), 7.68 (1H, ddd, *J* = 8.3, 4.9, 0.8 Hz), 7.47 (1H, ddd, *J* = 8.4, 7.3, 1.3 Hz), 7.40 (1H, td, *J* = 7.5, 1.0 Hz); ^13^C NMR (151 MHz, DMSO-*d*_6_) *δ* 186.89, 155.82, 147.69, 138.49, 135.51, 134.45, 133.32, 126.67, 125.95, 125.54, 125.10, 122.15, 122.10, 113.33.

#### 4.1.2. Synthesis of compounds 5a–r

A solution of *N*-pyridylsulfonyl indole (3) (0.34 mmol, 0.1 g) in 10 mL of ethanol was prepared, to which equimolar amounts (0.34 mmol) of substituted thiosemicarbazides (4a–r) were added, along with several drops of acetic acid as a catalyst. The reaction mixture was refluxed for 4 hours. The reaction progress was assessed by TLC using a 1 : 5 petroleum ether/ethyl acetate (v/v) mixture as the eluent. After the reaction was completed, as demonstrated by TLC, the precipitates were washed with ethanol and filtered to get the desired thiosemicarbazones (5a–r) in good to moderate yields.^32,33^

##### 4.1.2.1 (E)-*N*-(3-(methylthio)propyl)-2-((1-(pyridin-3-ylsulfonyl)-1*H*-indol-3-yl)methylene)hydrazine-1-carbothioamide (5a)

Yield: 94%, M.P: 209 °C, color: white; ^1^H NMR (600 MHz, DMSO-*d*_6_) *δ* 11.52 (1H, s), 9.23 (1H, d, *J* = 2.4 Hz), 8.85 (1H, dd, *J* = 4.9, 1.5 Hz), 8.44 (1H, dt, *J* = 8.3, 1.9 Hz), 8.35 (1H, s), 8.27 (2H, d, *J* = 12.4 Hz), 8.15 (1H, t, *J* = 6.0 Hz), 8.01 (1H, d, *J* = 8.3 Hz), 7.63 (1H, dd, *J* = 8.2, 4.8 Hz), 7.50–7.43 (1H, m), 7.37 (1H, t, *J* = 7.6 Hz), 3.67 (2H, q, *J* = 6.7 Hz), 2.54–2.48 (5H, m), 2.05 (3H, s), 1.88 (2H, p, *J* = 7.2 Hz); ^13^C NMR (151 MHz, DMSO) *δ* 177.04, 155.47, 147.36, 137.41, 135.15, 134.80, 133.48, 129.80, 126.96, 126.27, 124.98, 124.81, 123.42, 118.61, 113.25, 43.00, 30.93, 28.36, 14.84. ESI-HRMS (*m*/*z*): chemical formula: C_19_H_21_N_5_O_2_S_3_, calcd [M − H]^+^: 446.0779, found [M − H]^+^: 446.0792.

##### 4.1.2.2 (E)-*N*-phenyl-2-((1-(pyridin-3-ylsulfonyl)-1*H*-indol-3-yl)methylene)hydrazine-1-carbothioamide (5b)

Yield: 97%, M.P: 234 °C, color: white; ^1^H NMR (600 MHz, DMSO-*d*_6_) *δ* 11.87 (1H, s), 9.77 (1H, s), 9.24 (1H, d, *J* = 2.5 Hz), 8.86 (1H, dd, *J* = 4.9, 1.5 Hz), 8.45 (2H, d, *J* = 9.6 Hz), 8.38 (1H, s), 8.28 (1H, d, *J* = 7.9 Hz), 8.02 (1H, d, *J* = 8.3 Hz), 7.64 (1H, dd, *J* = 8.3, 4.8 Hz), 7.57 (2H, d, *J* = 8.0 Hz), 7.48–7.42 (1H, m), 7.37 (3H, td, *J* = 7.7, 3.4 Hz), 7.20 (1H, t, *J* = 7.4 Hz); ^13^C NMR (151 MHz, DMSO) *δ* 176.03, 155.49, 147.37, 139.35, 137.98, 135.15, 134.77, 133.48, 129.93, 128.31, 127.06, 126.27, 125.84, 125.70, 125.48, 125.01, 124.86, 123.32, 118.50, 113.24. ESI-HRMS (*m*/*z*): chemical formula: C_21_H_17_N_5_O_2_S_2_, calcd [M + H]^+^: 436.0902, found [M + H]^+^: 436.0895.

##### 4.1.2.3 (E)-*N*-(4-bromophenyl)-2-((1-(pyridin-3-ylsulfonyl)-1*H*-indol-3-yl)methylene)hydrazine-1-carbothioamide (5c)

Yield: 96%, M.P: 239 °C, color: white; ^1^H NMR (600 MHz, DMSO-*d*_6_) *δ* 11.96 (1H, s), 9.81 (1H, s), 9.24 (1H, d, *J* = 2.4 Hz), 8.86 (1H, dd, *J* = 4.9, 1.5 Hz), 8.47–8.42 (2H, m), 8.38 (1H, s), 8.27 (1H, d, *J* = 7.9 Hz), 8.02 (1H, d, *J* = 8.3 Hz), 7.64 (1H, dd, *J* = 8.2, 4.8 Hz), 7.59–7.52 (4 H, m), 7.45 (1H, ddd, *J* = 8.4, 7.3, 1.2 Hz), 7.37 (1H, t, *J* = 7.5 Hz); ^13^C NMR (151 MHz, DMSO) *δ* 175.96, 155.50, 147.37, 138.81, 138.35, 135.15, 134.76, 133.47, 131.11, 130.07, 127.82, 127.03, 126.28, 125.00, 124.85, 123.37, 118.44, 117.68, 113.23. ESI-HRMS (*m*/*z*): chemical formula: C_21_H_16_BrN_5_O_2_S_2_, calcd [M + H]^+^: 514.0007, found [M + H]^+^: 515.9981 (^81^Br).

##### 4.1.2.4 (E)-*N*-isobutyl-2-((1-(pyridin-3-ylsulfonyl)-1*H*-indol-3-yl)methylene)hydrazine-1-carbothioamide (5d)

Yield: 97%, M.P: 237 °C, color: white; ^1^H NMR (600 MHz, DMSO-*d*_6_) *δ* 11.52 (1H, s), 9.23 (1H, dd, *J* = 2.5, 0.8 Hz), 8.85 (1H, dd, *J* = 4.8, 1.6 Hz), 8.43 (1H, ddd, *J* = 8.2, 2.6, 1.6 Hz), 8.36 (1H, s), 8.28 (1H, s), 8.24–8.20 (1H, m), 8.05–7.99 (2H, m), 7.63 (1H, ddd, *J* = 8.2, 4.8, 0.8 Hz), 7.45 (1H, ddd, *J* = 8.4, 7.2, 1.3 Hz), 7.36 (1H, td, *J* = 7.6, 7.2, 1.0 Hz), 3.49–3.40 (2H, m), 1.99 (1H, hept, *J* = 6.8 Hz), 0.90 (6H, d, *J* = 6.7 Hz); ^13^C NMR (151 MHz, DMSO) *δ* 177.23, 155.43, 147.33, 137.21, 135.11, 134.81, 133.46, 129.80, 126.97, 126.25, 124.95, 124.77, 123.19, 118.61, 113.30, 50.85, 27.95, 20.08. ESI-HRMS (*m*/*z*): chemical formula: C_19_H_21_N_5_O_2_S_2_, calcd [M + H]^+^: 416.1215, found [M + H]^+^: 416.1203.

##### 4.1.2.5 (E)-*N*-(3-nitrophenyl)-2-((1-(pyridin-3-ylsulfonyl)-1*H*-indol-3-yl)methylene)hydrazine-1-carbothioamide (5e)

Yield: 95%, M.P: 227 °C, color: light yellow; ^1^H NMR (600 MHz, DMSO-*d*_6_) *δ* 12.20–12.06 (1H, m), 10.10 (1H, s), 9.24 (1H, dd, *J* = 2.5, 0.8 Hz), 8.86 (1H, dd, *J* = 4.8, 1.5 Hz), 8.65 (1H, t, *J* = 2.2 Hz), 8.48 (1H, s), 8.45 (1H, ddd, *J* = 8.2, 2.6, 1.6 Hz), 8.42 (1H, s), 8.30 (1H, d, *J* = 7.9 Hz), 8.09–7.98 (3 H, m), 7.70–7.59 (2H, m), 7.46 (1H, ddd, *J* = 8.4, 7.3, 1.3 Hz), 7.38 (1H, td, *J* = 7.6, 1.0 Hz); ^13^C NMR (151 MHz, DMSO) *δ* 176.03, 155.49, 147.49, 147.35, 140.66, 139.01, 135.14, 134.76, 133.45, 132.00, 130.33, 129.44, 126.99, 126.30, 124.99, 124.85, 123.44, 119.79, 118.36, 113.22. ESI-HRMS (*m*/*z*): chemical formula: C_21_H_16_N_6_O_4_S_2_, calcd [M − H]^+^: 479.0596, found [M − H]^+^: 479.0604.

##### 4.1.2.6 (E)-*N*-cyclohexyl-2-((1-(pyridin-3-ylsulfonyl)-1*H*-indol-3-yl)methylene)hydrazine-1-carbothioamide (5f)

Yield: 90%, M.P: 240 °C, color: white; ^1^H NMR (600 MHz, DMSO-*d*_6_) *δ* 11.54 (1H, s), 9.23 (1H, dd, *J* = 2.5, 0.7 Hz), 8.85 (1H, dd, *J* = 4.8, 1.5 Hz), 8.43 (1H, ddd, *J* = 8.2, 2.5, 1.5 Hz), 8.37 (1H, s), 8.28 (1H, s), 8.08 (1H, dd, *J* = 7.7, 1.3 Hz), 8.03 (1H, d, *J* = 8.4 Hz), 7.67–7.59 (2H, m), 7.46 (1H, ddd, *J* = 8.5, 7.3, 1.3 Hz), 7.39 (1H, td, *J* = 7.6, 1.1Hz), 4.25–4.04 (1H, m), 1.91 (2H, dq, *J* = 12.3, 3.8 Hz), 1.69 (2H, dp, *J* = 12.2, 3.7 Hz), 1.57 (1H, dq, *J* = 12.7, 3.9 Hz), 1.42 (2H, qd, *J* = 11.9, 3.3 Hz), 1.32 (2H, tdd, *J* = 14.9, 11.5, 3.3 Hz), 1.21 (1H, ddt, *J* = 15.0, 11.5, 5.6 Hz); ^13^C NMR (151 MHz, DMSO) *δ* 175.81, 155.44, 147.33, 137.16, 135.12, 134.82, 133.44, 129.79, 127.00, 126.27, 124.95, 124.88, 122.72, 118.52, 113.43, 52.21, 31.81, 25.19, 24.61. ESI-HRMS (*m*/*z*): chemical formula: C_21_H_23_N_5_O_2_S_2_, calcd [M + H]^+^: 442.1371, found [M + H]^+^: 442.1363.

##### 4.1.2.7 (E)-*N*-(2,6-difluorophenyl)-2-((1-(pyridin-3-ylsulfonyl)-1*H*-indol-3-yl)methylene)hydrazine-1-carbothioamide (5g)

Yield: 96%, M.P: 228 °C, color: off-white; ^1^H NMR (600 MHz, DMSO-*d*_6_) *δ* 12.11 (1H, s), 9.35 (1H, s), 9.25 (1H, d, *J* = 2.4 Hz), 8.86 (1H, dd, *J* = 4.8, 1.4 Hz), 8.45 (2H, d, *J* = 5.0 Hz), 8.39 (2H, d, *J* = 6.8 Hz), 8.01 (1H, d, *J* = 8.4 Hz), 7.65 (1H, dd, *J* = 8.2, 4.8 Hz), 7.44 (2H, dt, *J* = 17.2, 8.2 Hz), 7.34 (1H, t, *J* = 7.6 Hz), 7.17 (2H, t, *J* = 8.1Hz); ^13^C NMR (151 MHz, DMSO) *δ* 177.92, 160.15, 160.12, 158.51, 158.48, 155.52, 147.40, 139.08, 135.18, 134.75, 133.50, 130.45, 129.23, 129.17, 129.10, 126.86, 126.32, 125.03, 124.81, 123.77, 118.30, 117.42, 117.31, 117.20, 113.13, 111.94, 111.91, 111.79. ESI-HRMS (*m*/*z*): chemical formula: C_21_H_15_F_2_N_5_O_2_S_2_, calcd [M − H]^+^: 470.0557, found [M − H]^+^: 470.0564.

##### 4.1.2.8 (E)-*N*-(4-bromo-2-fluorophenyl)-2-((1-(pyridin-3-ylsulfonyl)-1*H*-indol-3-yl)methylene)hydrazine-1-carbothioamide (5h)

Yield: 96%, M.P: 233 °C, color: white; ^1^H NMR (600 MHz, DMSO-*d*_6_) *δ* 12.11 (1H, s), 9.59 (1H, s), 9.25 (1H, d, *J* = 2.4 Hz), 8.86 (1H, dd, *J* = 4.9, 1.5 Hz), 8.48–8.43 (2H, m), 8.39 (1H, s), 8.32 (1H, d, *J* = 7.9 Hz), 8.02 (1H, d, *J* = 8.4 Hz), 7.68 (1H, t, *J* = 8.4 Hz), 7.66–7.61 (2H, m), 7.48–7.41 (2H, m), 7.39–7.32 (1H, m); ^13^C NMR (151 MHz, DMSO) *δ* 176.66, 157.84, 156.18, 155.52, 147.39, 138.88, 135.18, 134.78, 133.48, 130.96, 130.55, 127.32, 127.29, 127.21, 126.85, 126.33, 125.02, 124.82, 123.48, 119.24, 119.08, 118.90, 118.84, 118.27, 113.22. ESI-HRMS (*m*/*z*): chemical formula: C_21_H_15_FBrN_5_O_2_S_2_, calcd [M + H]^+^: 531.9913, found [M + H]^+^: 531.9747.

##### 4.1.2.9 (E)-*N*-(naphthalen-1-yl)-2-((1-(pyridin-3-ylsulfonyl)-1*H*-indol-3-yl)methylene)hydrazine-1-carbothioamide (5i)

Yield: 94%, M.P: 237 °C, color: white; ^1^H NMR (600 MHz, DMSO-*d*_6_) *δ* 11.97 (1H, s), 10.01 (1H, s), 9.25 (1H, dd, *J* = 2.5, 0.8 Hz), 8.87 (1H, dd, *J* = 4.9, 1.5 Hz), 8.48 (1H, s), 8.47–8.43 (2H, m), 8.40 (1H, d, *J* = 8.0 Hz), 8.02 (1H, dt, *J* = 8.3, 0.9 Hz), 7.99–7.95 (1H, m), 7.89 (2H, tt, *J* = 8.2, 2.7 Hz), 7.65 (1H, ddd, *J* = 8.2, 4.9, 0.8 Hz), 7.59–7.54 (2H, m), 7.53 (2H, dtd, *J* = 6.8, 5.4, 4.9, 3.4 Hz), 7.43 (1H, ddd, *J* = 8.5, 7.3, 1.3 Hz), 7.30 (1H, ddd, *J* = 8.1, 7.3, 1.0 Hz); ^13^C NMR (151 MHz, DMSO) *δ* 177.75, 155.49, 147.37, 138.07, 135.90, 135.15, 134.77, 133.89, 133.50, 130.75, 130.00, 128.21, 127.07, 127.03, 126.56, 126.31, 126.26, 126.19, 125.60, 125.02, 124.76, 123.60, 123.34, 118.59, 113.17. ESI-HRMS (*m*/*z*): chemical formula: C_25_H_20_N_5_O_2_S_2_, calcd [M + H]^+^: 486.1058, found [M + H]^+^: 486.1051.

##### 4.1.2.10 (E)-*N*-(4-chlorophenyl)-2-((1-(pyridin-3-ylsulfonyl)-1*H*-indol-3-yl)methylene)hydrazine-1-carbothioamide (5j)

Yield: 98%, M.P: 236 °C, color: white; ^1^H NMR (600 MHz, DMSO-*d*_6_) *δ* 11.95 (1H, s), 9.82 (1H, s), 9.24 (1H, d, *J* = 2.5 Hz), 8.86 (1H, dd, *J* = 4.8, 1.5 Hz), 8.47–8.43 (2H, m), 8.38 (1H, s), 8.27 (1H, d, *J* = 7.9 Hz), 8.02 (1H, d, *J* = 8.3 Hz), 7.45 (1H, ddd, *J* = 8.4, 7.2, 1.3 Hz), 7.43–7.39 (2H, m), 7.39–7.34 (1H, m); ^13^C NMR (151 MHz, DMSO) *δ* 176.06, 155.50, 147.37, 138.38, 138.34, 135.16, 134.77, 133.48, 130.06, 129.49, 128.19, 127.53, 127.04, 126.29, 125.01, 124.86, 123.38, 118.45, 113.23. ESI-HRMS (*m*/*z*): chemical formula: C_21_H_16_ClN_5_O_2_S_2_, calcd [M − H]^+^: 468.0356, found [M − H]^+^: 468.0363.

##### 4.1.2.11 (E)-*N*-(2,6-dichlorophenyl)-2-((1-(pyridin-3-ylsulfonyl)-1*H*-indol-3-yl)methylene)hydrazine-1-carbothioamide (5k)

Yield: 97%, M.P: 224 °C, color: white; ^1^H NMR (600 MHz, DMSO-*d*_6_) *δ* 12.01 (1H, s), 9.63 (1H, s), 9.25 (1H, d, *J* = 2.5 Hz), 8.86 (1H, dd, *J* = 4.9, 1.5 Hz), 8.46–8.40 (3 H, m), 8.38 (1H, s), 8.01 (1H, d, *J* = 8.4 Hz), 7.64 (1H, ddd, *J* = 8.3, 4.8, 0.8 Hz), 7.54 (2H, d, *J* = 8.1Hz), 7.45 (1H, ddd, *J* = 8.4, 7.2, 1.2 Hz), 7.35 (2H, dt, *J* = 21.6, 7.6 Hz); ^13^C NMR (151 MHz, DMSO) *δ* 177.05, 155.49, 147.37, 138.60, 135.52, 135.15, 134.76, 133.48, 130.33, 129.49, 128.44, 126.89, 126.29, 125.01, 124.80, 123.89, 118.43, 113.10. ESI-HRMS (*m*/*z*): chemical formula: C_21_H_15_Cl_2_N_5_O_2_S_2_, calcd [M + H]^+^: 504.0122, found [M + H]^+^: 504.0110.

##### 4.1.2.12 (E)-*N*-(2,6-dimethylphenyl)-2-((1-(pyridin-3-ylsulfonyl)-1*H*-indol-3-yl)methylene)hydrazine-1-carbothioamide (5l)

Yield: 99%, M.P: 238 °C, color: white; ^1^H NMR (600 MHz, DMSO-*d*_6_) *δ* 11.76 (1H, s), 9.43 (1H, s), 9.26–9.22 (1H, m), 8.86 (1H, dd, *J* = 4.9, 1.5 Hz), 8.47–8.39 (3 H, m), 8.37 (1H, s), 8.00 (1H, d, *J* = 8.3 Hz), 7.64 (1H, ddd, *J* = 8.2, 4.9, 0.8 Hz), 7.44 (1H, ddd, *J* = 8.4, 7.3, 1.2 Hz), 7.37–7.30 (1H, m), 7.11 (3 H, q, *J* = 5.6 Hz), 2.19 (6 H, s); ^13^C NMR (151 MHz, DMSO) *δ* 176.73, 155.46, 147.35, 137.72, 137.56, 136.75, 135.12, 134.77, 133.50, 129.89, 127.74, 127.07, 127.01, 126.23, 125.00, 124.78, 123.82, 118.68, 113.09, 18.20. ESI-HRMS (*m*/*z*): chemical formula: C_23_H_21_N_5_O_2_S_2_, calcd [M + H]^+^: 464.1215, found [M + H]^+^: 464.1204.

##### 4.1.2.13 (E)-*N*-(2,4-dimethylphenyl)-2-((1-(pyridin-3-ylsulfonyl)-1*H*-indol-3-yl)methylene)hydrazine-1-carbothioamide (5m)

Yield: 96%, M.P: 229 °C, color: white; ^1^H NMR (600 MHz, DMSO-*d*_6_) *δ* 11.77 (1H, s), 9.48 (1H, s), 9.24 (1H, d, *J* = 2.5 Hz), 8.86 (1H, dd, *J* = 4.9, 1.5 Hz), 8.47–8.41 (2H, m), 8.38–8.30 (2H, m), 8.01 (1H, d, *J* = 8.3 Hz), 7.64 (1H, dd, *J* = 8.2, 4.8 Hz), 7.47–7.41 (1H, m), 7.33 (1H, t, *J* = 7.6 Hz), 7.21 (1H, d, *J* = 8.0 Hz), 7.07 (1H, d, *J* = 2.1Hz), 7.01 (1H, dd, *J* = 8.1, 2.1Hz), 2.28 (3 H, s), 2.19 (3 H, s); ^13^C NMR (151 MHz, DMSO) *δ* 176.78, 155.48, 147.36, 137.71, 135.90, 135.71, 135.14, 135.05, 134.76, 133.50, 130.75, 129.89, 128.58, 127.00, 126.56, 126.24, 125.00, 124.77, 123.54, 118.57, 113.17, 20.75, 17.86. ESI-HRMS (*m*/*z*): chemical formula: C_23_H_21_N_5_O_2_S_2_, calcd [M + H]^+^: 464.1215, found [M + H]^+^: 464.1203.

##### 4.1.2.14 (E)-2-((1-(pyridin-3-ylsulfonyl)-1*H*-indol-3-yl)methylene)-*N*-(*p*-tolyl)hydrazine-1-carbothioamide (5n)

Yield: 96%, M.P: 233 °C, color: white; ^1^H NMR (600 MHz, DMSO-*d*_6_) *δ* 11.82 (1H, s), 9.69 (1H, s), 9.24 (1H, d, *J* = 2.4 Hz), 8.86 (1H, dd, *J* = 4.9, 1.5 Hz), 8.44 (2H, d, *J* = 7.9 Hz), 8.37 (1H, s), 8.26 (1H, d, *J* = 7.9 Hz), 8.02 (1H, d, *J* = 8.3 Hz), 7.64 (1H, dd, *J* = 8.2, 4.8 Hz), 7.48–7.40 (3 H, m), 7.39–7.33 (1H, m), 7.16 (2H, d, *J* = 8.1Hz), 2.30 (3 H, s); ^13^C NMR (151 MHz, DMSO) *δ* 176.10, 155.48, 147.36, 137.79, 136.78, 135.15, 134.76, 134.70, 133.48, 129.82, 128.77, 127.08, 126.26, 125.86, 125.74, 125.00, 124.84, 123.28, 118.53, 113.24, 20.73. ESI-HRMS (*m*/*z*): chemical formula: C_22_H_19_N_5_O_2_S_2_, calcd [M + H]^+^: 450.1058, found [M + H]^+^: 450.1047.

##### 4.1.2.15 (E)-*N*-(4-nitrophenyl)-2-((1-(pyridin-3-ylsulfonyl)-1*H*-indol-3-yl)methylene)hydrazine-1-carbothioamide (5o)

Yield: 98%, M.P: 237 °C, color: yellow; ^1^H NMR (600 MHz, DMSO-*d*_6_) *δ* 12.23 (1H, s), 10.20 (1H, s), 9.24 (1H, d, *J* = 2.3 Hz), 8.86 (1H, dd, *J* = 4.8, 1.5 Hz), 8.49 (1H, s), 8.45 (1H, dt, *J* = 8.4, 2.0 Hz), 8.42 (1H, s), 8.24 (3H, dd, *J* = 9.3, 7.4 Hz), 8.03 (3H, dd, *J* = 8.6, 6.4 Hz), 7.64 (1H, dd, *J* = 8.3, 4.8 Hz), 7.48–7.42 (1H, m), 7.38 (1H, t, *J* = 7.6 Hz); ^13^C NMR (151 MHz, DMSO) *δ* 175.42, 155.52, 147.38, 145.75, 143.51, 139.25, 135.18, 134.79, 133.46, 130.56, 126.98, 126.35, 125.01, 124.89, 124.04, 123.37, 118.32, 113.28. ESI-HRMS (*m*/*z*): chemical formula: C_21_H_16_N_6_O_4_S_2_, calcd [M − H]^+^: 479.0596, found [M − H]^+^: 479.0604.

##### 4.1.2.16 (E)-2-((1-(pyridin-3-ylsulfonyl)-1*H*-indol-3-yl)methylene)-*N*-(2-(trifluoromethyl)phenyl)hydrazine-1-carbothioamide (5p)

Yield: 97%, M.P: 221 °C, color: off-white; ^1^H NMR (600 MHz, DMSO-*d*_6_) *δ* 12.11 (1H, s), 9.55 (1H, s), 9.25 (1H, dd, *J* = 2.5, 0.8 Hz), 8.86 (1H, dd, *J* = 4.8, 1.5 Hz), 8.48–8.43 (2H, m), 8.40 (1H, s), 8.29 (1H, d, *J* = 7.9 Hz), 8.02 (1H, dt, *J* = 8.4, 0.9 Hz), 7.77–7.73 (2H, m), 7.71 (1H, td, *J* = 7.7, 1.4 Hz), 7.64 (1H, ddd, *J* = 8.2, 4.8, 0.8 Hz), 7.52–7.47 (1H, m), 7.44 (1H, ddd, *J* = 8.4, 7.3, 1.2 Hz), 7.31 (1H, ddd, *J* = 8.2, 7.3, 1.0 Hz); ^13^C NMR (151 MHz, DMSO) *δ* 176.98, 155.53, 147.41, 138.79, 137.37, 135.18, 134.81, 133.49, 132.56, 132.03, 130.82, 127.24, 126.77, 126.35, 126.15, 126.12, 125.02, 124.75, 123.42, 118.48, 118.26, 113.42, 113.24. ESI-HRMS (*m*/*z*): chemical formula: C_22_H_16_F_3_N_5_O_2_S_2_, calcd [M + H]^+^: 504.0776, found [M + H]^+^: 504.0763.

##### 4.1.2.17 (E)-*N*-(3-fluorophenyl)-2-((1-(pyridin-3-ylsulfonyl)-1*H*-indol-3-yl)methylene)hydrazine-1-carbothioamide (5q)

Yield: 97%, M.P: 236 °C, color: white; ^1^H NMR (600 MHz, DMSO-*d*_6_) *δ* 11.98 (1H, s), 9.85 (1H, s), 9.24 (1H, dd, *J* = 2.5, 0.8 Hz), 8.86 (1H, dd, *J* = 4.9, 1.6 Hz), 8.49–8.42 (2H, m), 8.39 (1H, s), 8.27 (1H, d, *J* = 7.8 Hz), 8.06–7.99 (1H, m), 7.68–7.59 (2H, m), 7.50–7.34 (4 H, m), 7.02 (1H, tdd, *J* = 8.5, 2.7, 1.3 Hz); ^13^C NMR (151 MHz, DMSO) *δ* 175.77, 162.67, 160.75, 155.47, 147.34, 141.12, 141.03, 138.43, 135.13, 134.75, 133.45, 130.12, 129.77, 129.69, 127.01, 126.27, 124.98, 124.85, 123.31, 121.30, 118.40, 113.22, 112.26, 112.07, 111.94, 111.77. ESI-HRMS (*m*/*z*): chemical formula: C_21_H_16_FN_5_O_2_S_2_, calcd [M + H]^+^: 454.0808, found [M + H]^+^: 454.0795.

##### 4.1.2.18 (E)-*N*-(4-methoxyphenyl)-2-((1-(pyridin-3-ylsulfonyl)-1*H*-indol-3-yl)methylene)hydrazine-1-carbothioamide (5r)

Yield: 98%, M.P: 232 °C, color: white; ^1^H NMR (600 MHz, DMSO-*d*_6_) *δ* 11.78 (1H, s), 9.65 (1H, s), 9.24 (1H, dd, *J* = 2.5, 0.8 Hz), 8.86 (1H, dd, *J* = 4.8, 1.5 Hz), 8.48–8.42 (2H, m), 8.37 (1H, s), 8.28 (1H, d, *J* = 7.9 Hz), 8.02 (1H, dt, *J* = 8.4, 0.9 Hz), 7.64 (1H, ddd, *J* = 8.2, 4.8, 0.8 Hz), 7.47–7.32 (4H, m), 6.96–6.90 (2H, m), 3.76 (3H, s); ^13^C NMR (151 MHz, DMSO) *δ* 176.42, 157.18, 155.45, 147.32, 137.67, 135.11, 134.74, 133.47, 132.23, 129.71, 127.69, 127.60, 127.06, 126.22, 124.97, 124.80, 123.31, 118.53, 113.50, 113.19, 55.41. ESI-HRMS (*m*/*z*): chemical formula: C_22_H_19_N_5_O_3_S_2_, calcd [M + H]^+^: 466.1008, found [M + H]^+^: 466.0997.

### 4.2 Cytotoxicity

#### 4.2.1 Cell cultures

Human bronchial epithelial cells (BEAS-2B, ECACC) and human lung adenocarcinoma cells (A549, CCL-185, ATCC) were cultured in DMEM-F12 medium (Gibco, MD, USA) supplemented with 10% fetal bovine serum (FBS) (Gibco, MD, USA) and 1% penicillin–streptomycin. Cells were maintained under standard incubation conditions at 37 °C in a humidified atmosphere containing 5% CO_2_. For cytotoxicity evaluation, the synthesized compounds were tested on BEAS-2B and HUVEC cells to assess their potential effects on non-cancerous cell lines, whereas A549 cells were employed as a lung cancer model to investigate their antiproliferative activity.^22^

#### 4.2.2 MTT cell viability assay

The cytotoxic activity of the synthesized compounds was determined in A549 and BEAS-2B cell lines using the MTT colorimetric assay. Cells were seeded into 96-well plates at a density of 1 × 10^4^ cells per well and allowed to adhere overnight under standard culture conditions. The following day, cells were treated with seven different concentrations of each compound for 24 hours. DMSO was used as the vehicle control, and its final concentration did not exceed 1% in any experimental group. Following treatment, MTT solution was added to each well to obtain a final concentration of 0.1 mg mL^−1^, and the plates were incubated at 37 °C for 4 hours to allow the formation of insoluble formazan crystals. The medium was then carefully aspirated, and the crystals were solubilized in DMSO. After 30 minutes of incubation in the dark at room temperature, absorbance was measured at 570 nm using a microplate reader (BioTek Instruments, Inc., USA).^34^ Cell viability was calculated as a percentage relative to the untreated control group. All experiments were performed in triplicate and repeated at least three independent times. Results are expressed as mean ± standard deviation (SD). IC_50_ values were calculated from dose–response curves using nonlinear regression analysis. The selectivity of each compound toward cancer cells was expressed as the selectivity index (SI), calculated using the formula: SI = IC_50_ (healthy cells)/IC_50_ (cancer cells).^22^

### 4.3 Apoptosis analysis (Annexin V/PI)

Apoptosis induction was evaluated in A549 using an Annexin V/propidium iodide (PI) staining kit followed by flow cytometric analysis. Cells were cultured in high-glucose DMEM supplemented with 10% fetal bovine serum (FBS) and 1% penicillin/streptomycin under standard conditions (37 °C, 5% CO_2_). A549 cells were seeded into 6-well plates at a density of 4 × 10^5^ cells/well and treated for 24 h with compound 5p and the reference drug sorafenib at 1, 5, and 10 µM. After treatment, cells were trypsinized, collected, and washed with cold PBS. The cell pellets were resuspended in 500 µL of 1 × Annexin V binding buffer at a final density of 2 × 10^5^ cells per mL, followed by staining with 5 µL Annexin V and 10 µL PI. Samples were gently vortexed and incubated for 15 min at 25 °C in the dark, then washed and resuspended in 400 µL of 1 × Annexin V binding buffer. Finally, apoptotic populations were quantified using a CytoFLEX flow cytometer, and the percentages of viable (H), early apoptotic (EA), late apoptotic (LA), and necrotic (N) cells were determined.^35,36^

### 4.4 Computational studies

Molecular docking and molecular dynamics (MD) simulations were conducted using the Schrödinger molecular modeling suite (release 2025–1) through the Maestro interface (v14.3), with Desmond employed for MD calculations. Protein and ligand preparation procedures were carried out according to our established group protocols.^37,38^ The crystal structures of EGFR (PDB ID: 3POZ), VEGFR-1 (PDB ID: 3HNG), and VEGFR-2 (PDB ID: 4ASE) were retrieved from the Protein Data Bank and processed using the Protein Preparation Wizard module, including the addition of hydrogens, assignment of bond orders, and optimization of hydrogen-bonding networks. Docking studies were performed using Glide in extra precision (XP) mode, followed by Induced Fit Docking (IFD) to account for receptor flexibility.^39^ For each ligand, 20 binding poses were generated, and the most favorable conformations were selected based on IFD scoring. Binding free energies (Δ*G*_bind) were subsequently estimated using Prime MM-GBSA calculations with the VSGB solvation model to further refine and rank the ligand–protein complexes.^40,41^

Molecular dynamics simulations were performed using Desmond (Release 2024–3). The selected protein–ligand complexes were placed in an orthorhombic simulation box and solvated with the TIP4P explicit water model. Systems were neutralized with appropriate counter ions, and 0.15 M NaCl was added to approximate physiological ionic strength. All simulations were conducted using the OPLS4 force field. After energy minimization, the systems were equilibrated sequentially under NVT and NPT ensembles. A 250 ns production simulation was subsequently carried out at 300 K and 1.01325 bar, employing the Nosé–Hoover thermostat and the Martyna–Tobias–Klein barostat for temperature and pressure control, respectively. The stability of the protein–ligand complexes and their overall conformational behavior during the simulation were assessed based on root mean square deviation (RMSD) analysis.^42,43^

The pharmacokinetic profiles of the compounds were assessed using the QikProp module (Schrödinger, 2024), which predicts essential ADME (absorption, distribution, metabolism, and excretion) parameters. The calculated descriptors were utilized to evaluate drug-likeness and potential oral bioavailability, based on well-established structure–property relationships derived from experimental data.^42^

## Author contributions

Zahra Batool, Rushba Saman Masood, Norah A. Albekairi: investigation, formal analysis, data curation. Tugce Salduz, Fatma Betul Yoladi, Asif Rasool: formal analysis, validation, data curation, software. Fahri Akbas, Feyzi Sinan Tokali: biological activity, data curation, software, writing original draft. Abdulrahman Alshammari: validation, resources, funding acquisition, review & editing: Halil Şenol and Zahid Shafiq: conceptualization, supervision, writing – review original draft and review & editing.

## Conflicts of interest

The authors have declared no conflicts of interest.

## Supplementary Material

RA-016-D6RA02731H-s001

## Data Availability

All data generated or analyzed during this study are included in this published article and its supplementary information (SI). Supplementary information: ^1^H NMR, ^13^C NMR, and HRMS spectra of all the new compounds are available as supporting material. Furthermore, the cytotoxicity IC_50_ graphs were given in Supplementary Material file. See DOI: https://doi.org/10.1039/d6ra02731h.
