# Supplementary material for: Identification of selective N-pyridinsulfonyl indole based thiosemicarbazone derivatives as potential antiproliferative agents against lung cancer cells
Source: RSC Adv. 2026 Jul 2;16(34):33196–212. doi: 10.1039/d6ra02731h (PMC13326682; doi:10.1039/d6ra02731h)
Supplement: RA-016-D6RA02731H-s001 [file RA-016-D6RA02731H-s001.pdf]

Supplementary Information (SI) for RSC Advances.
This journal is © The Royal Society of Chemistry 2026

# SUPPLEMENTARY MATERIAL

## $^{1}$HNMR of 3

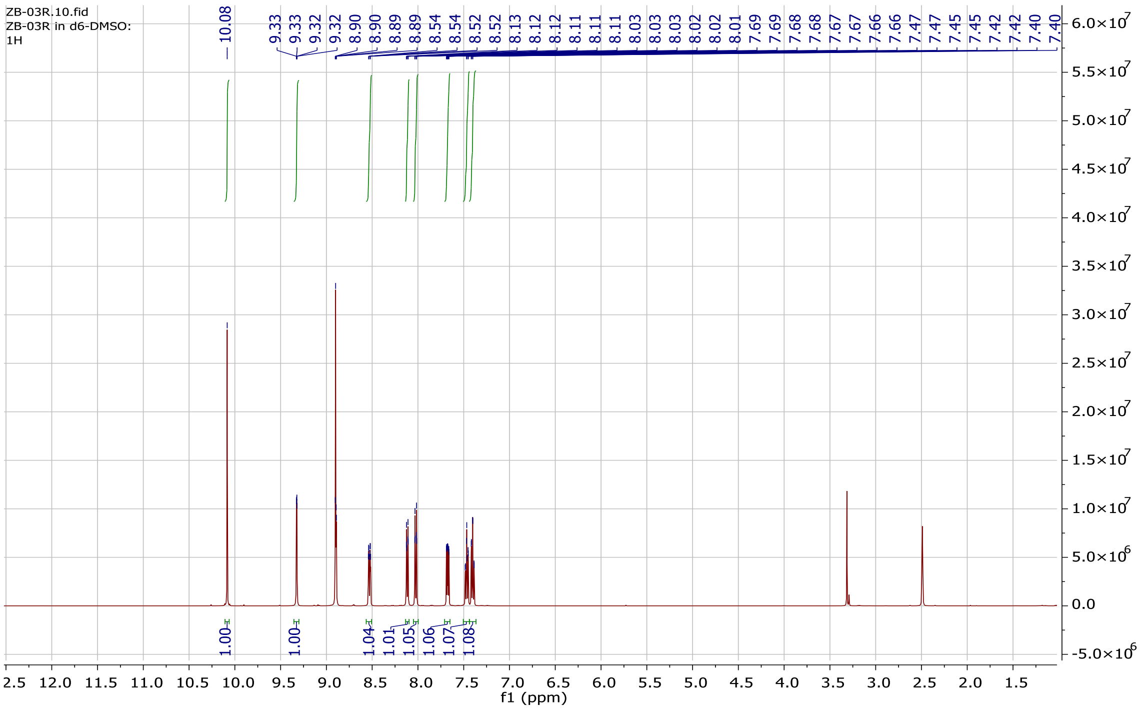


## $^{13}$CNMR of 3

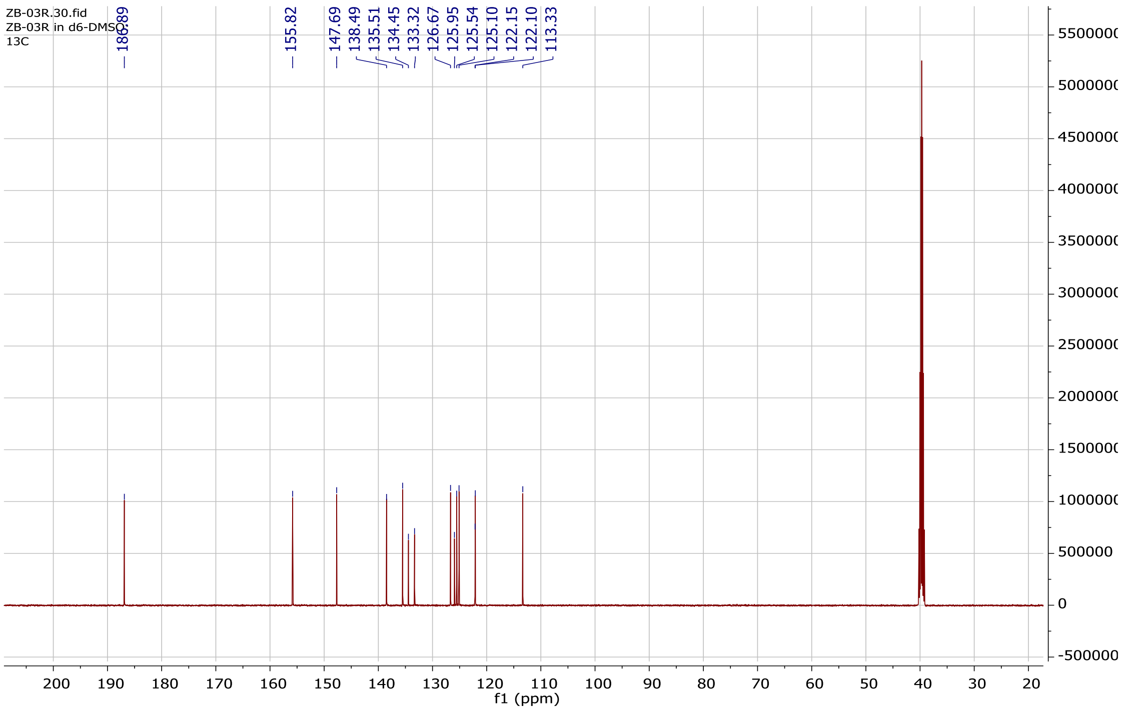

## $^{1}$HNMR of 5a

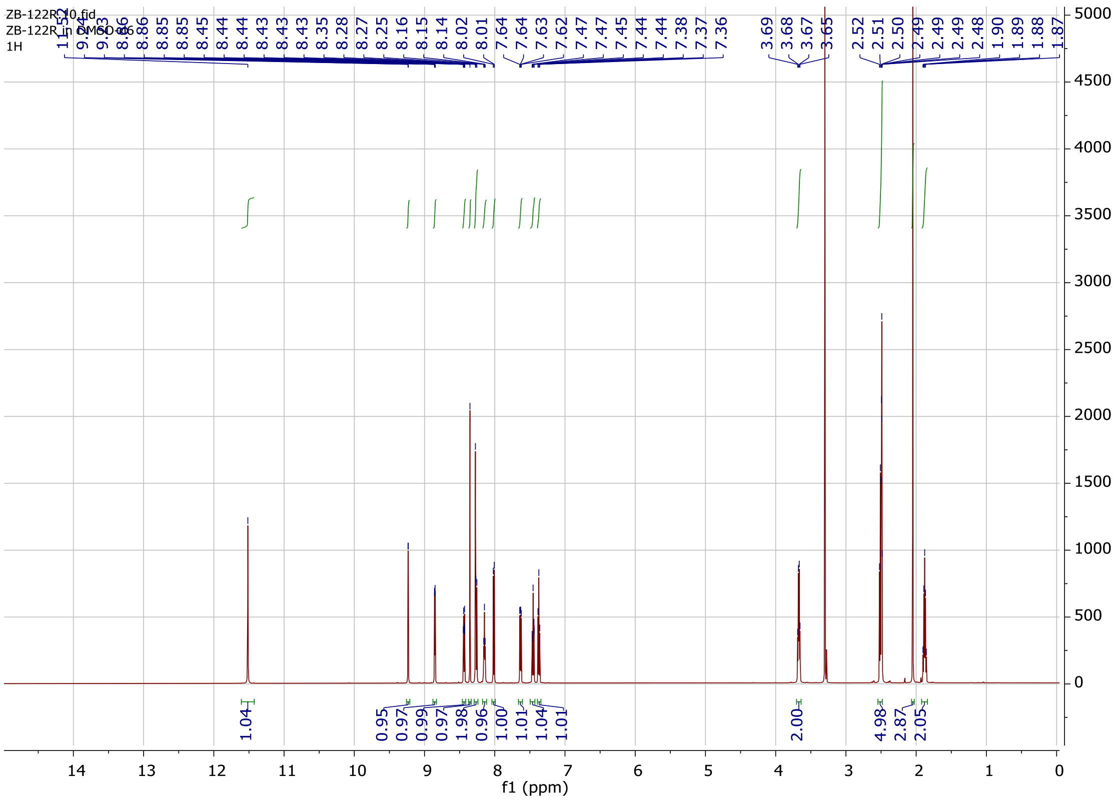

## $^{13}$CNMR of 5a

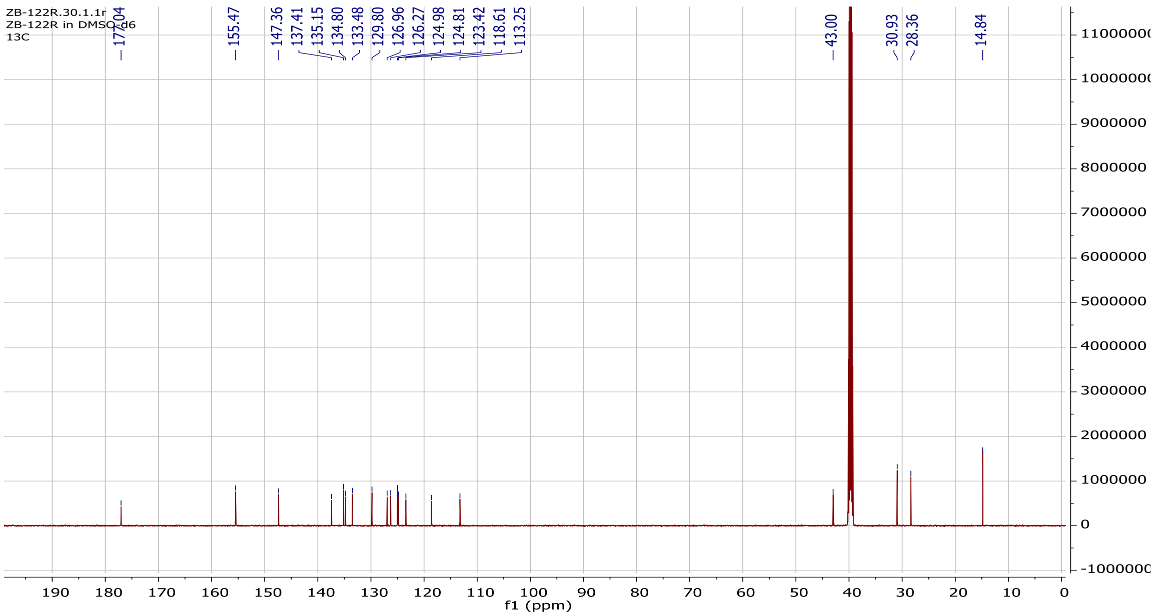

## HRMS of 5a

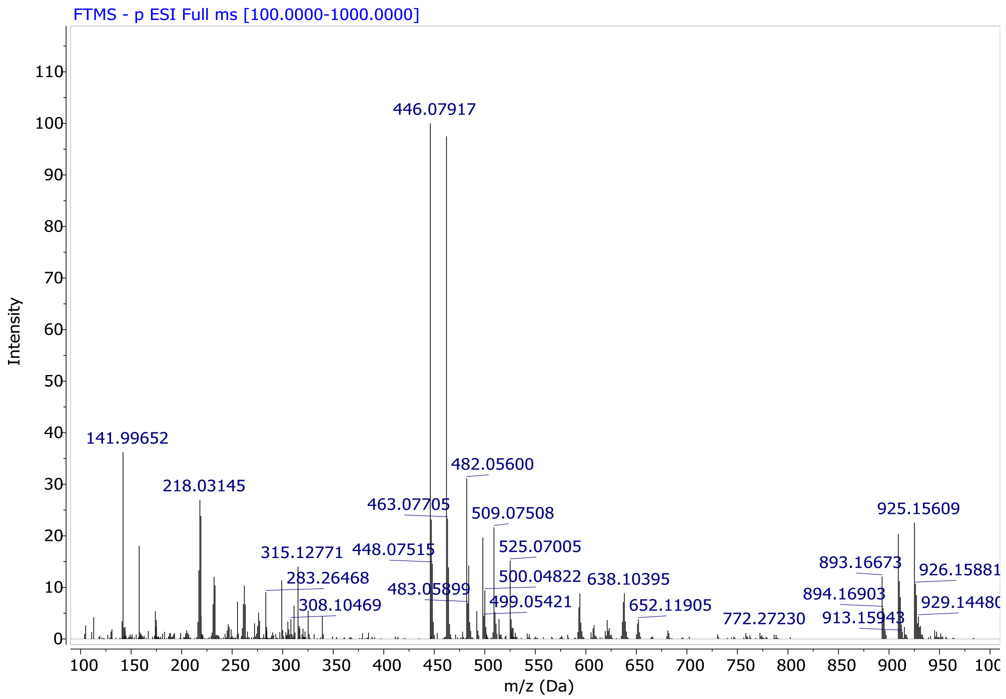


## $^1$HNMR of 5b

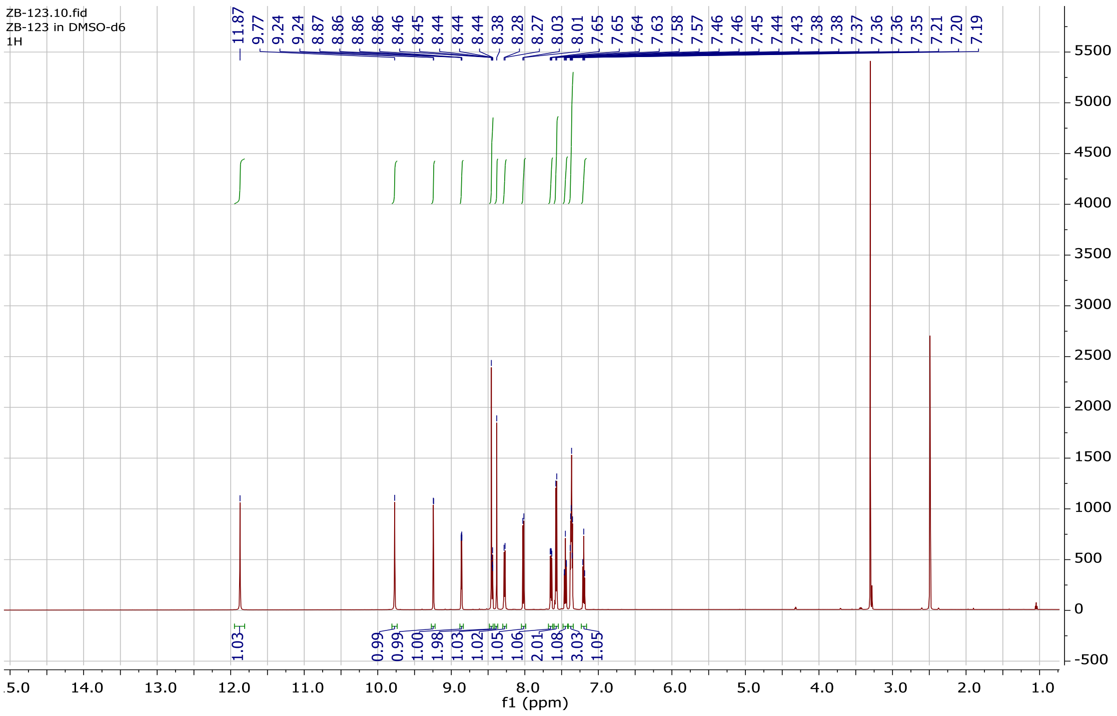

**$^{13}$CNMR of 5b**

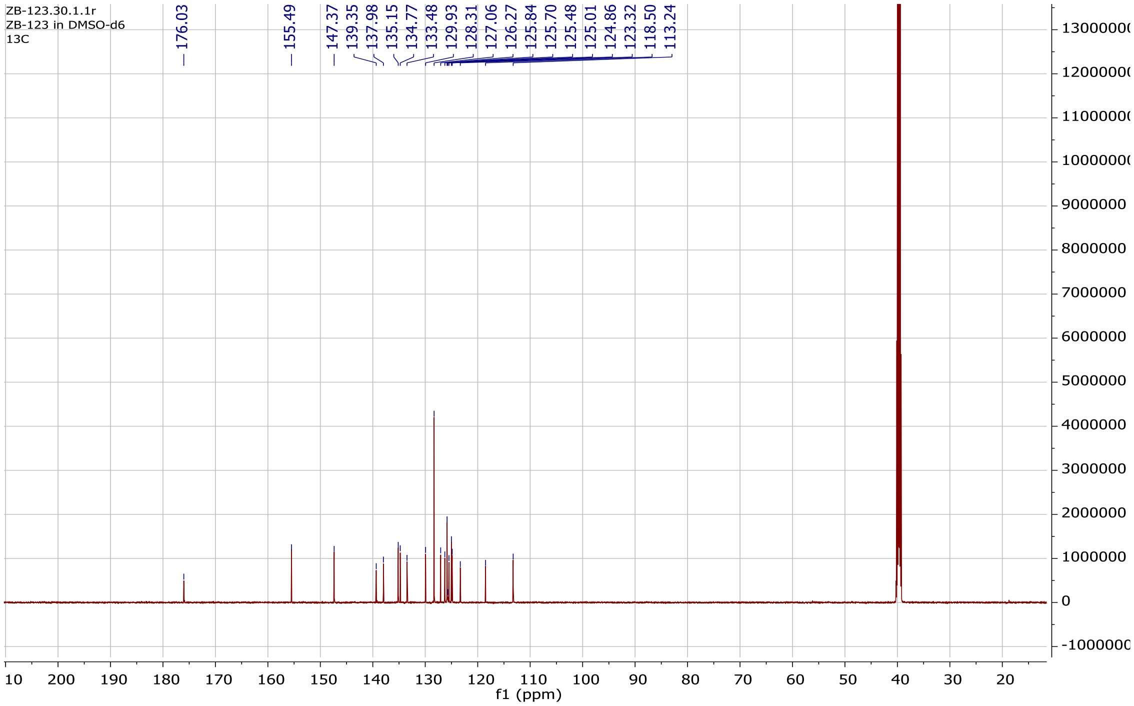


**HRMS of 5b**

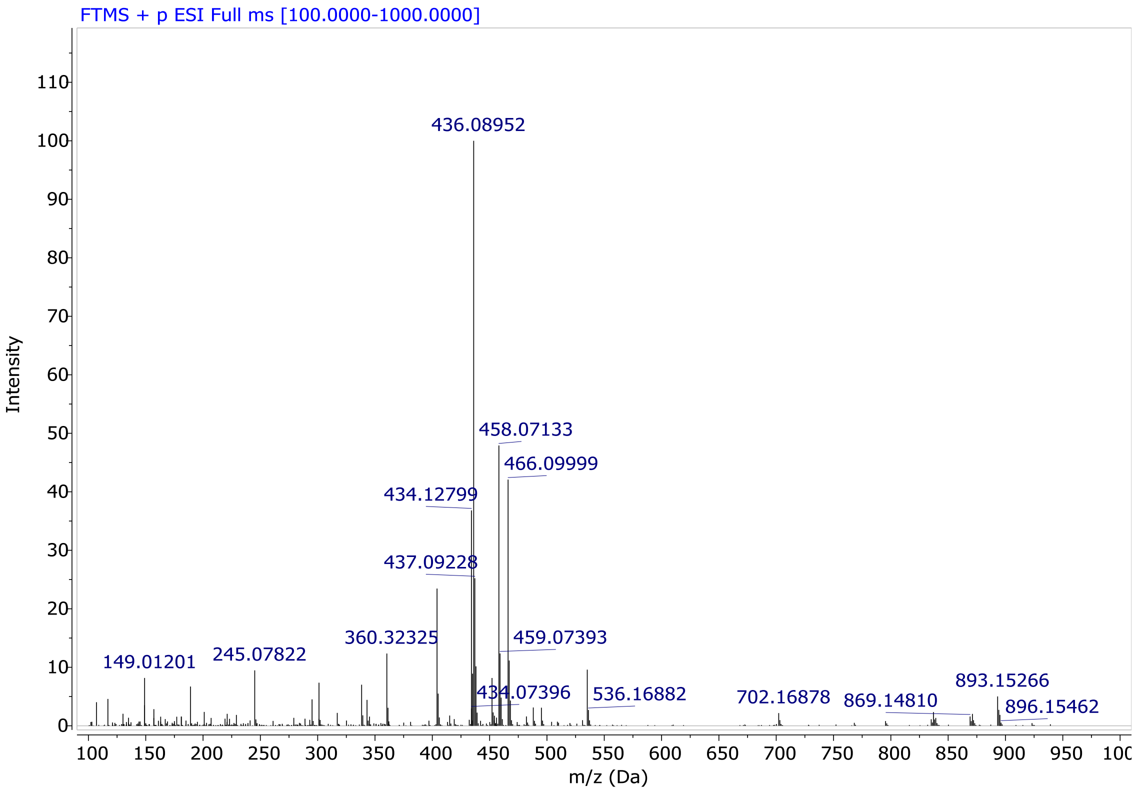

## $^{1}$HNMR of 5c

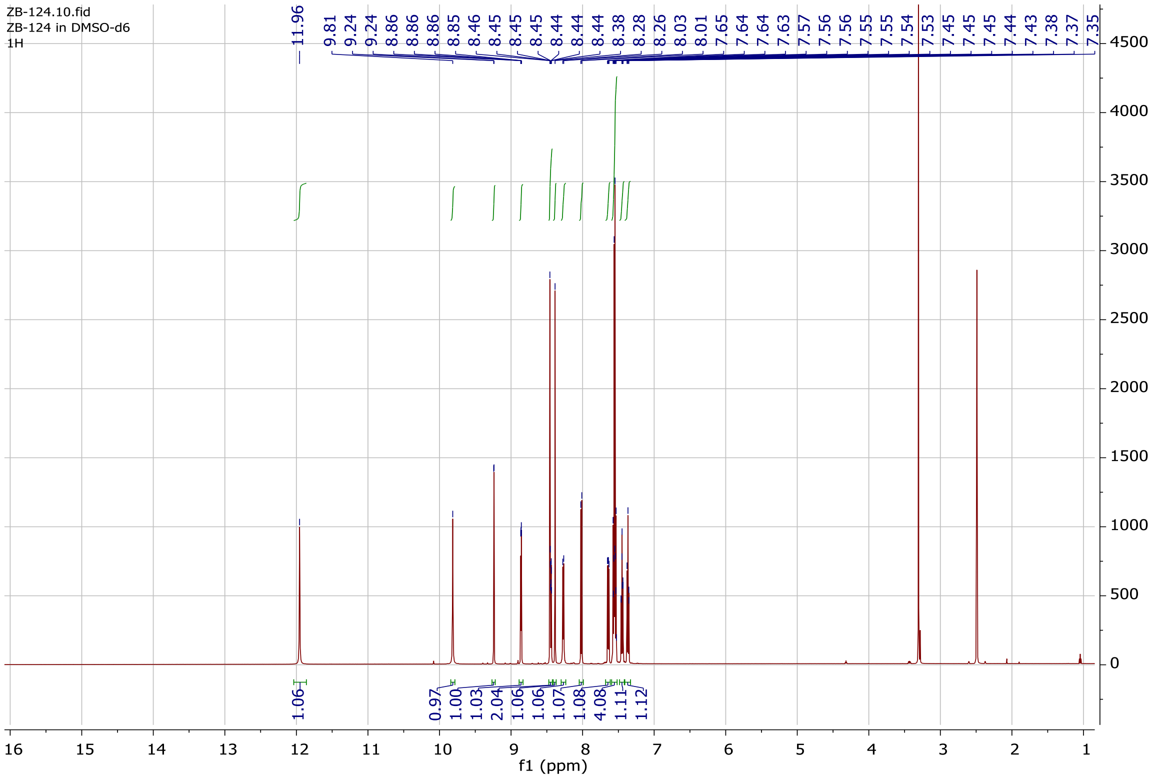

## $^{13}$CNMR of 5c

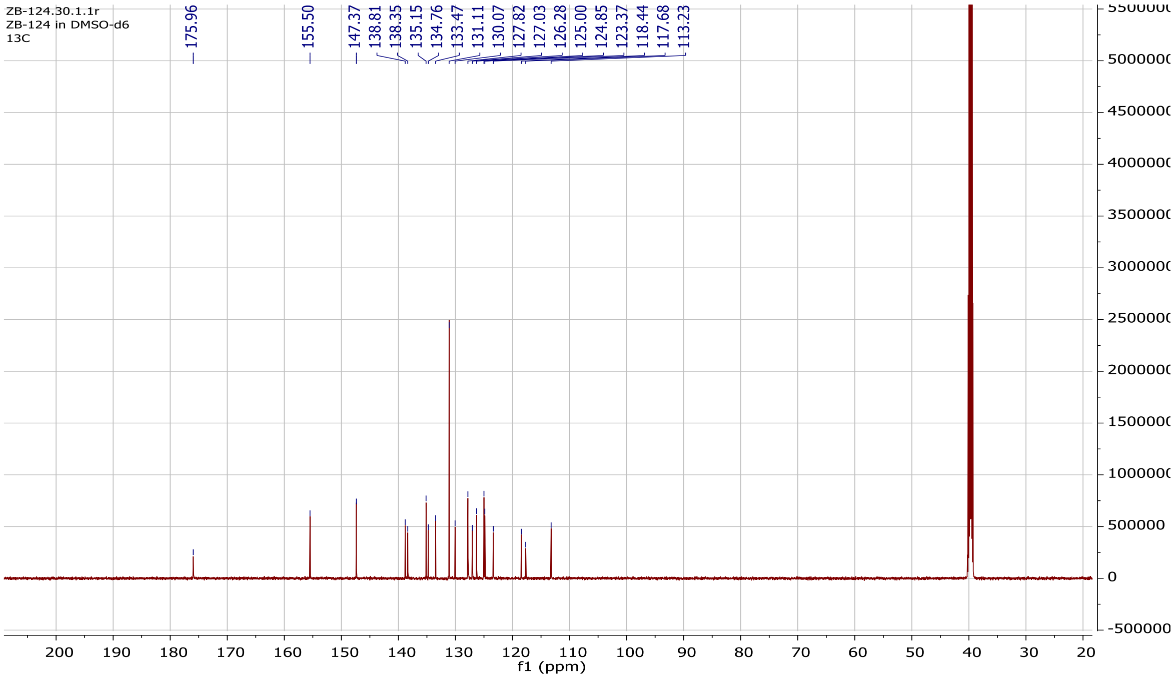

## HRMS of 5c

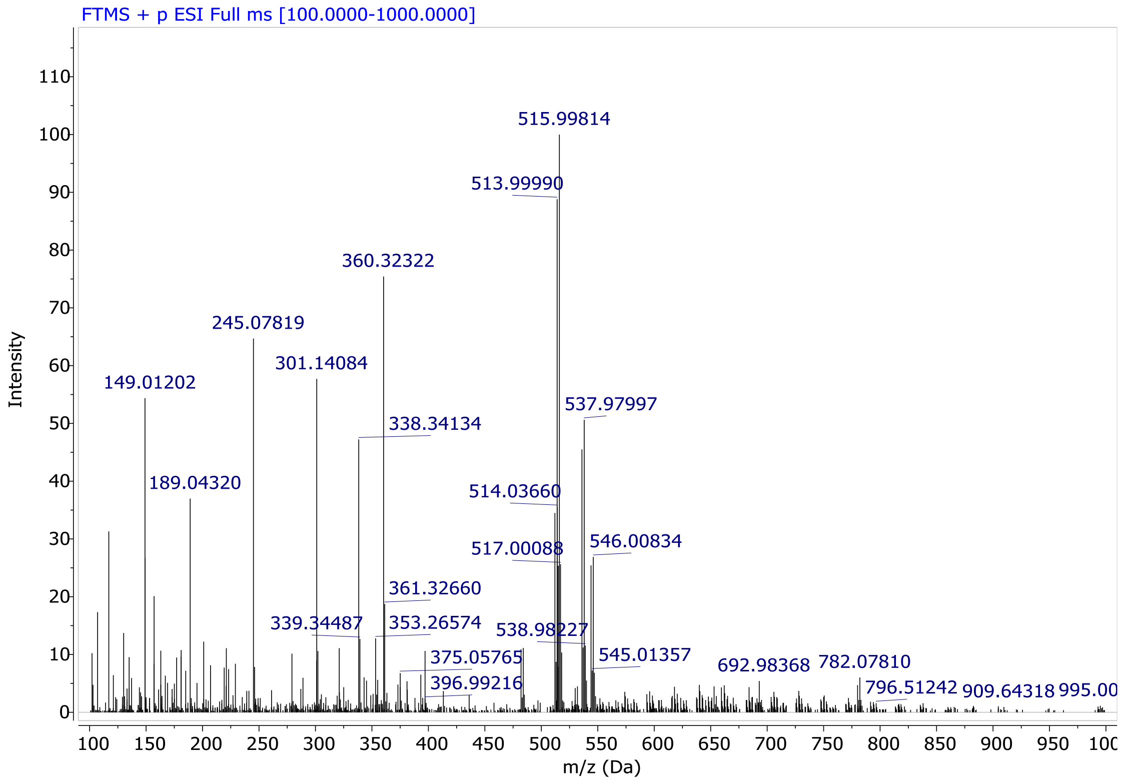


## $^{1}$HNMR of 5d

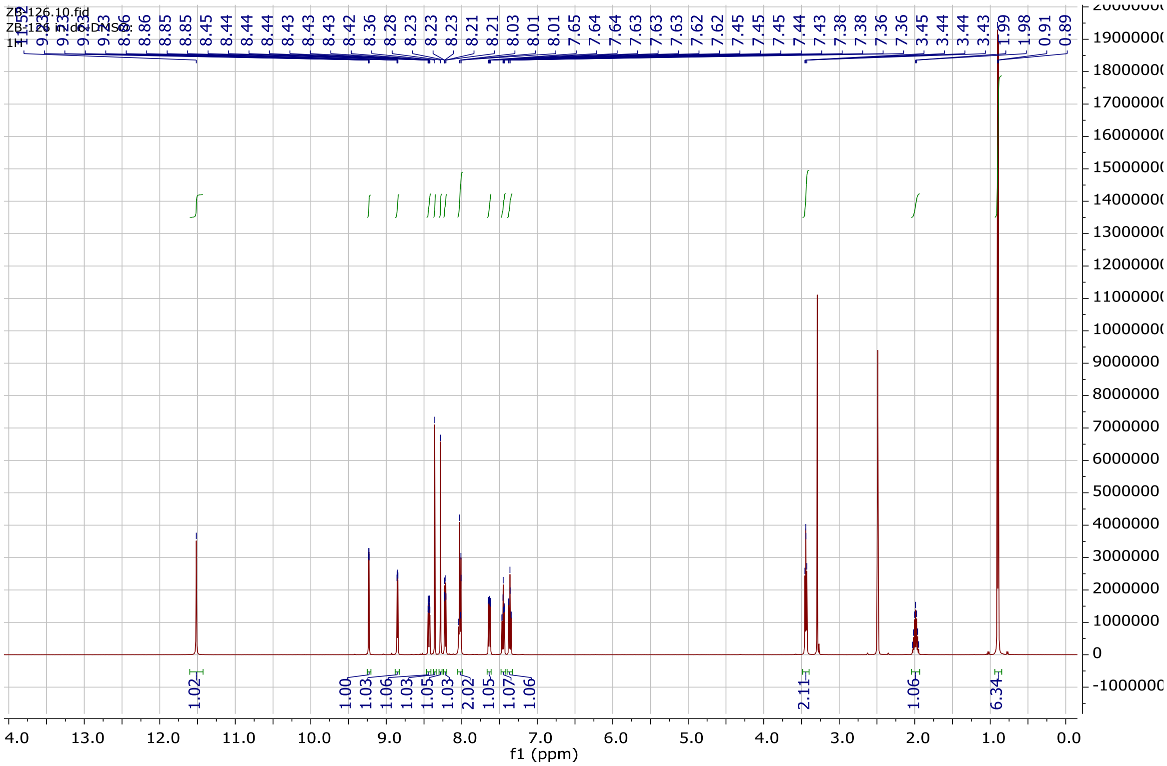

**$^{13}$CNMR of 5d**

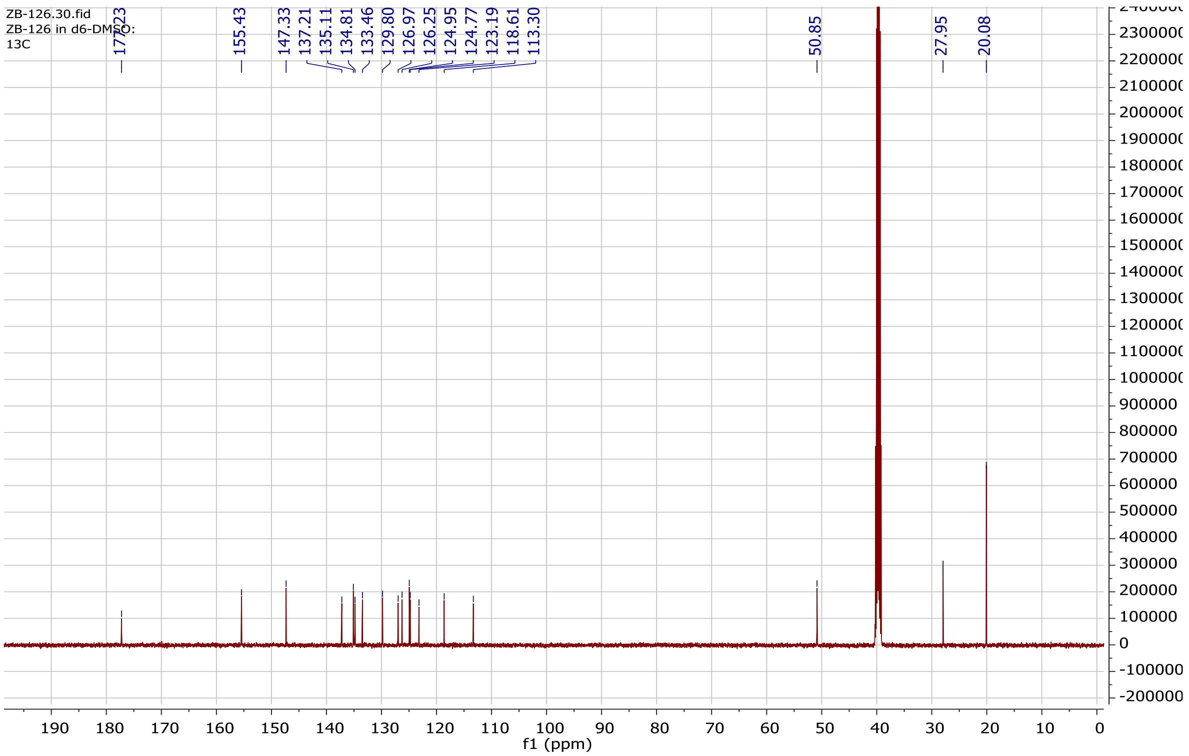


**HRMS of 5d**

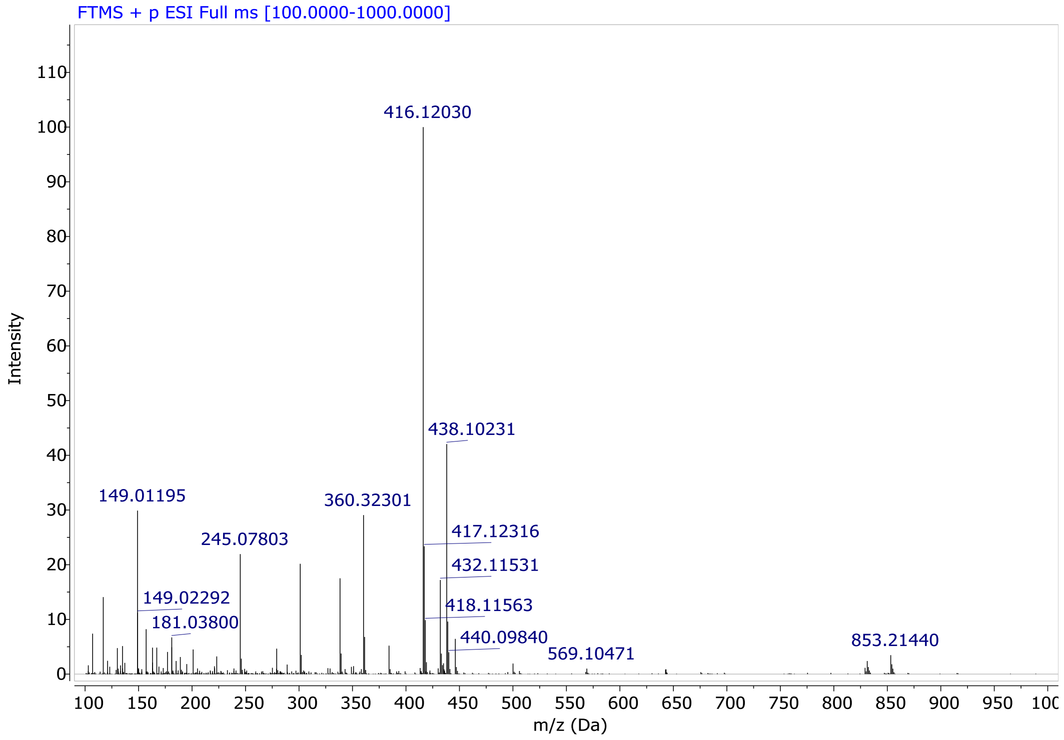

## $^{1}$HNMR of 5e

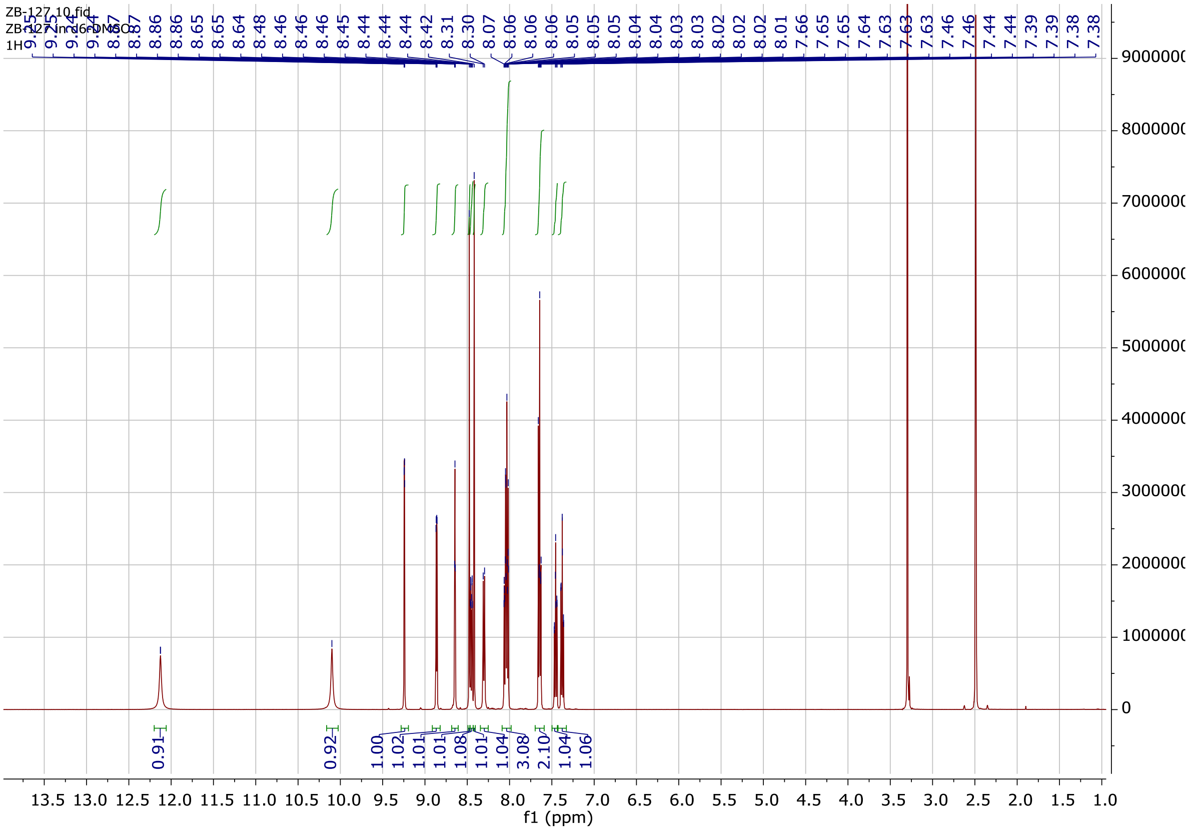

## $^{13}$CNMR of 5e

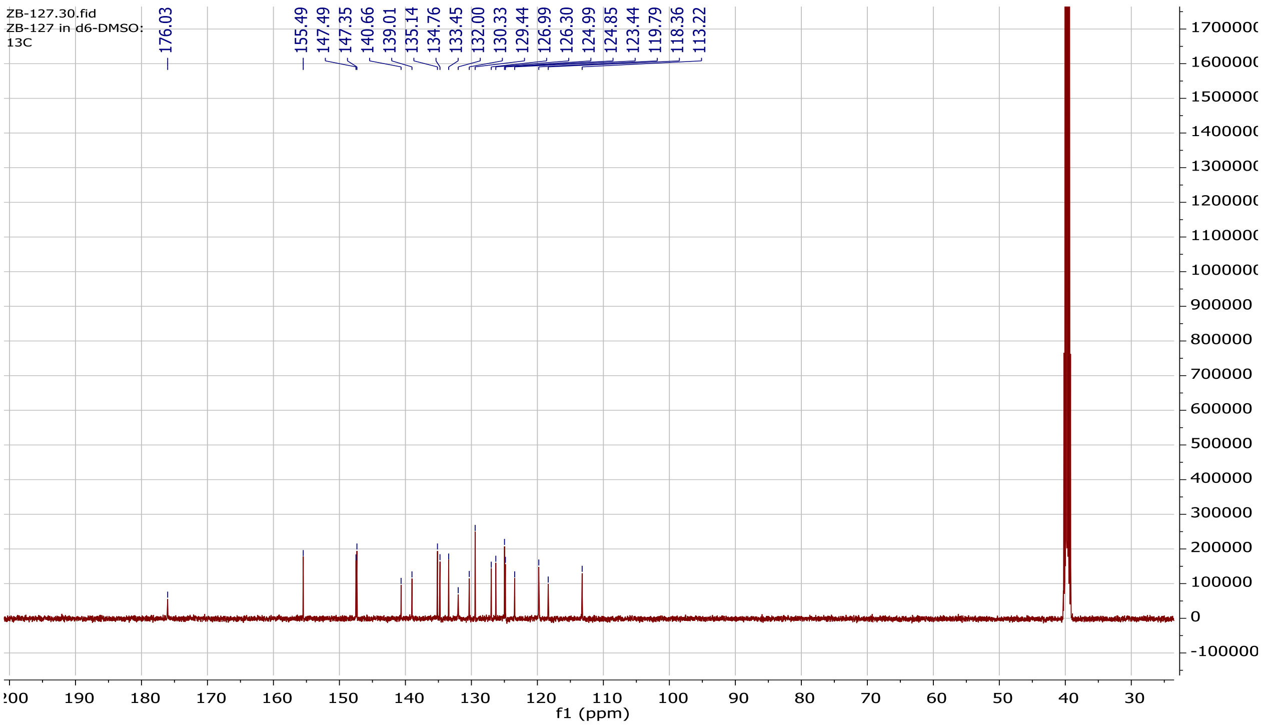

## HRMS of 5e

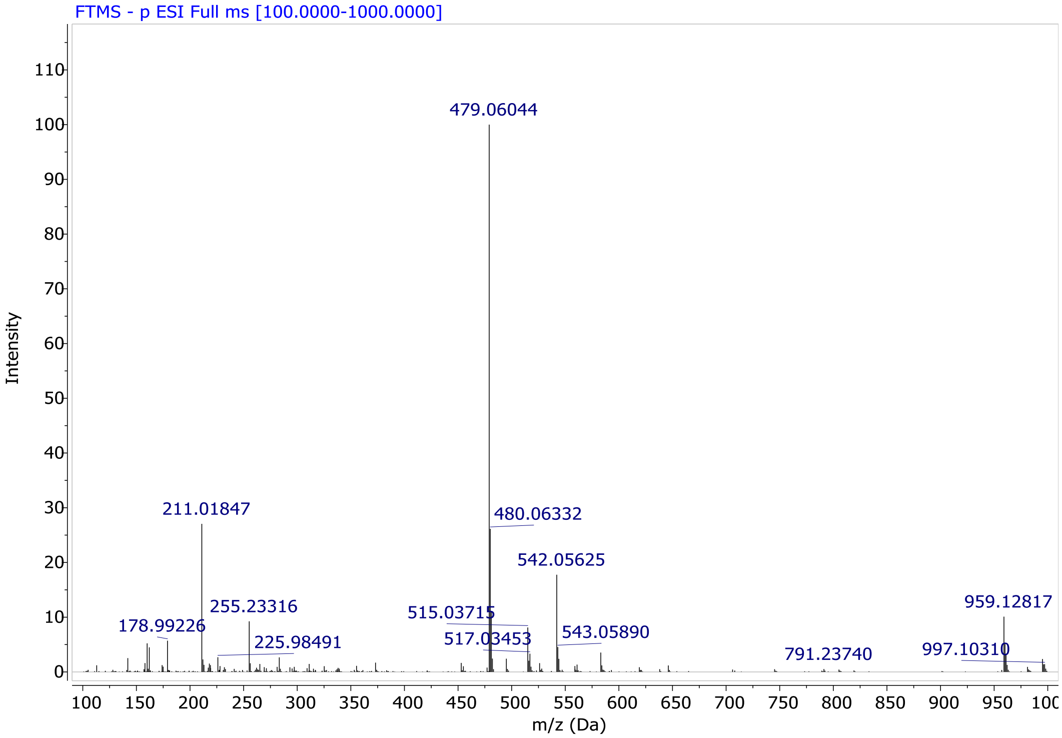


## $^{1}$HNMR of 5f

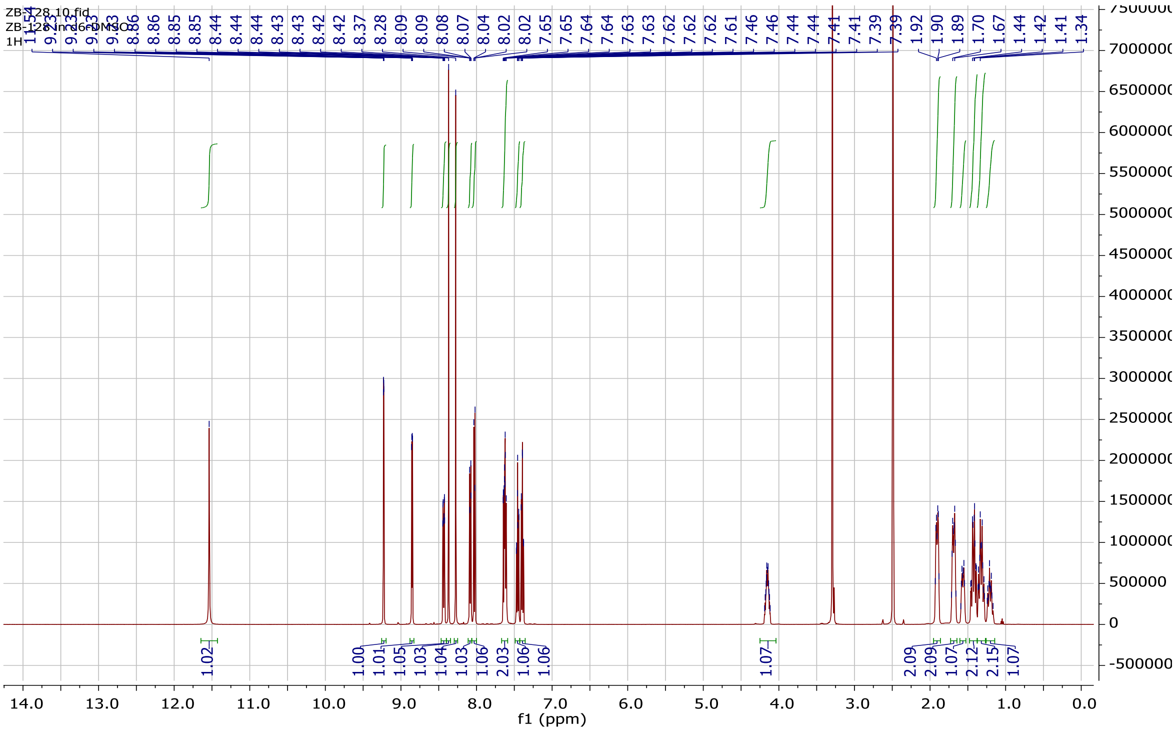

## $^{13}$CNMR of 5f

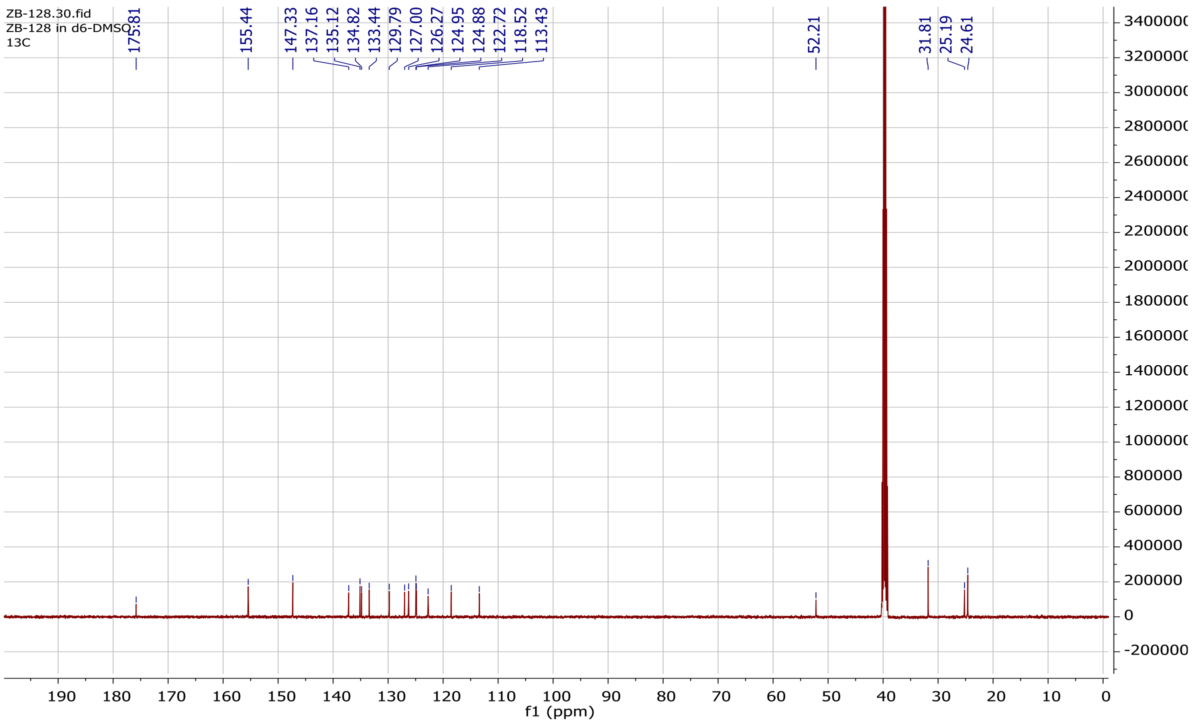


## HRMS of 5f

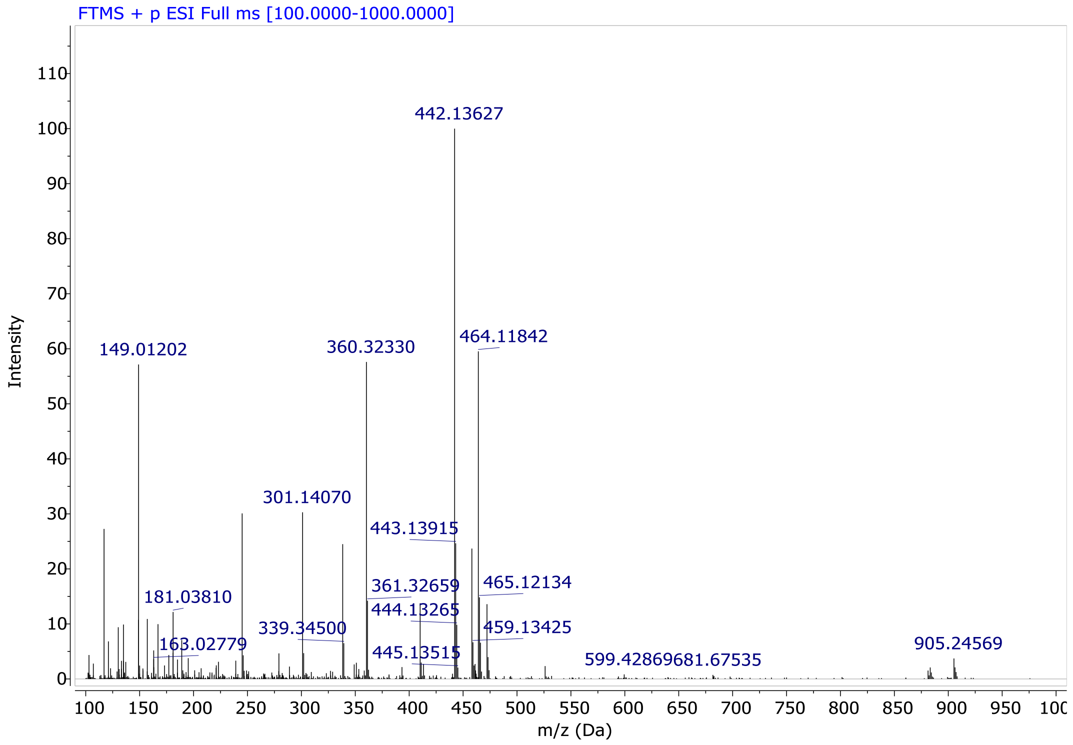

## $^{1}$HNMR of 5g

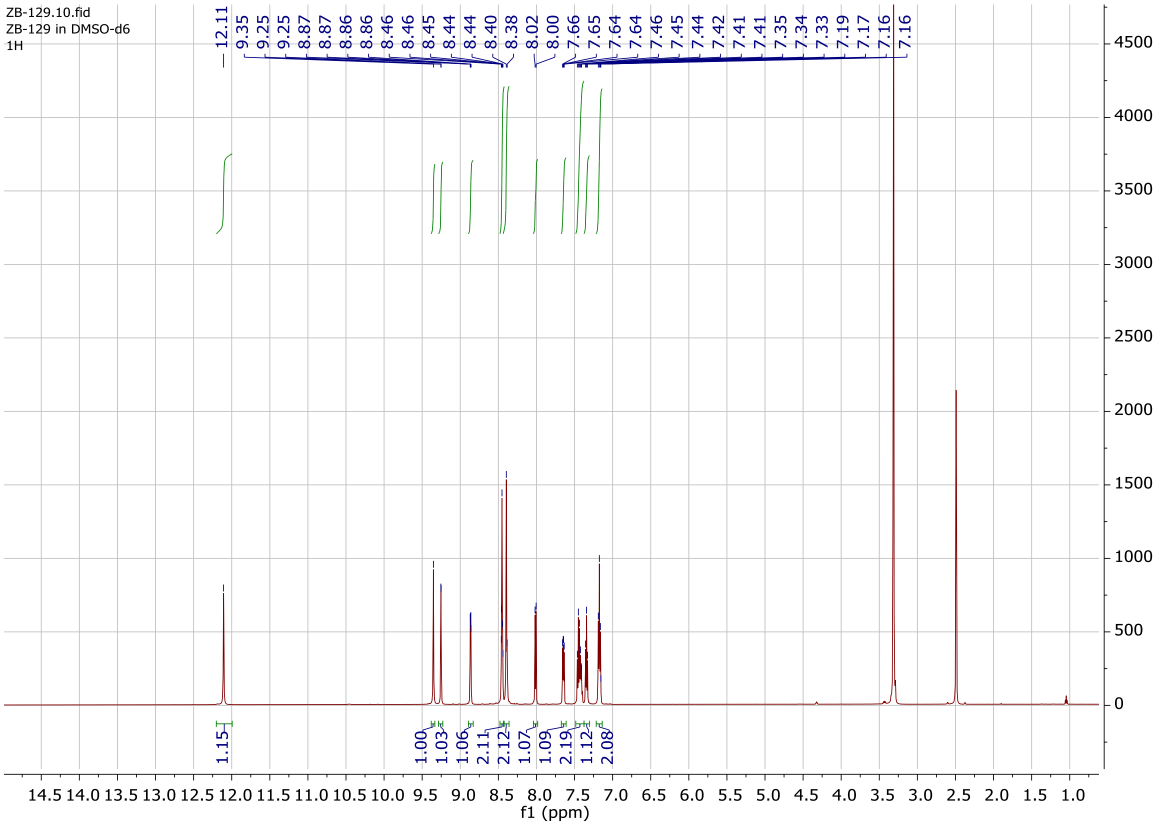


## $^{13}$C NMR of 5g

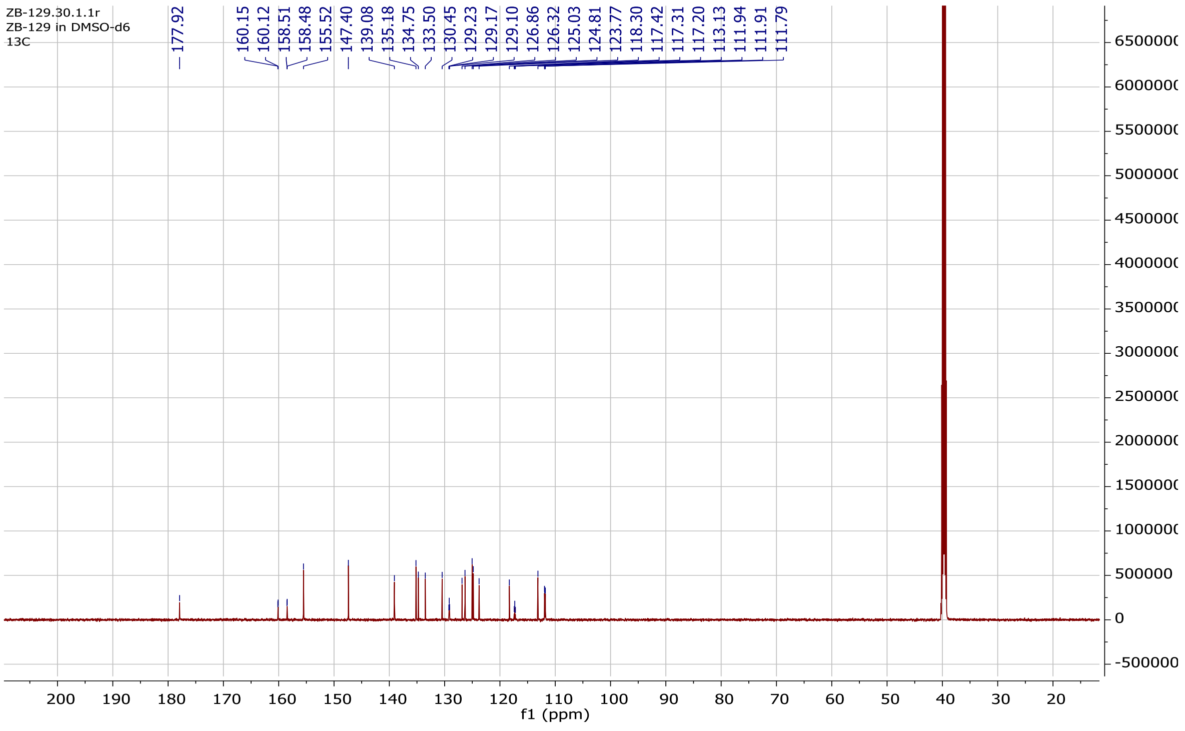

## HRMS of 5g

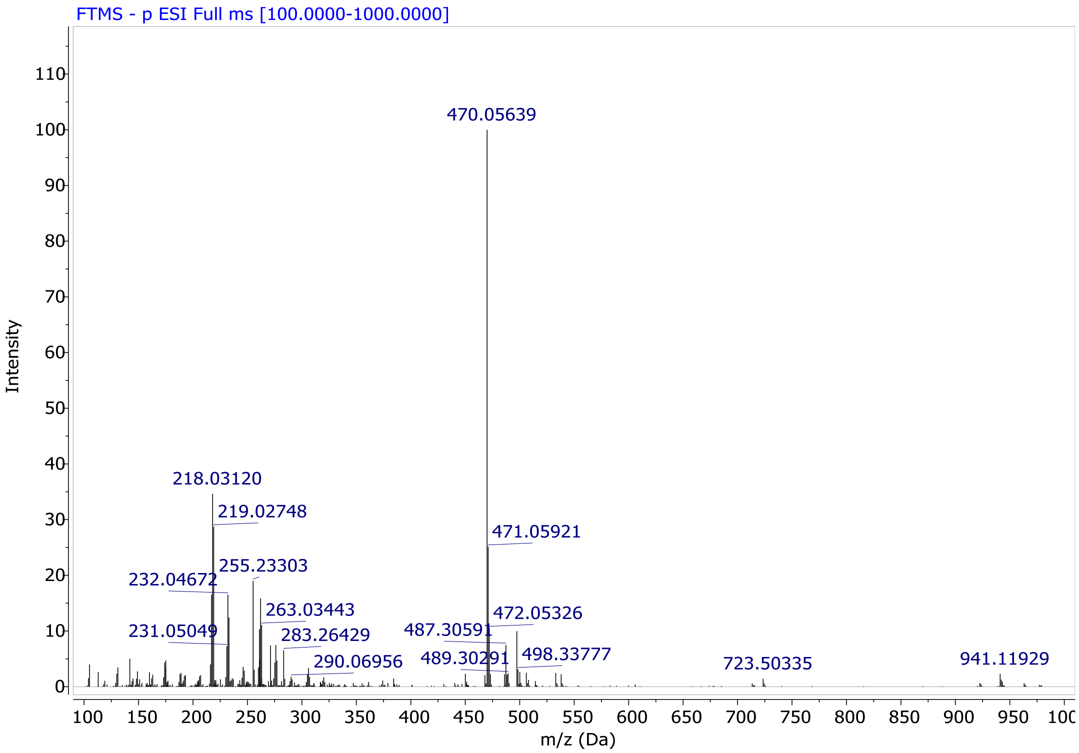


## $^{1}$HNMR of 5h

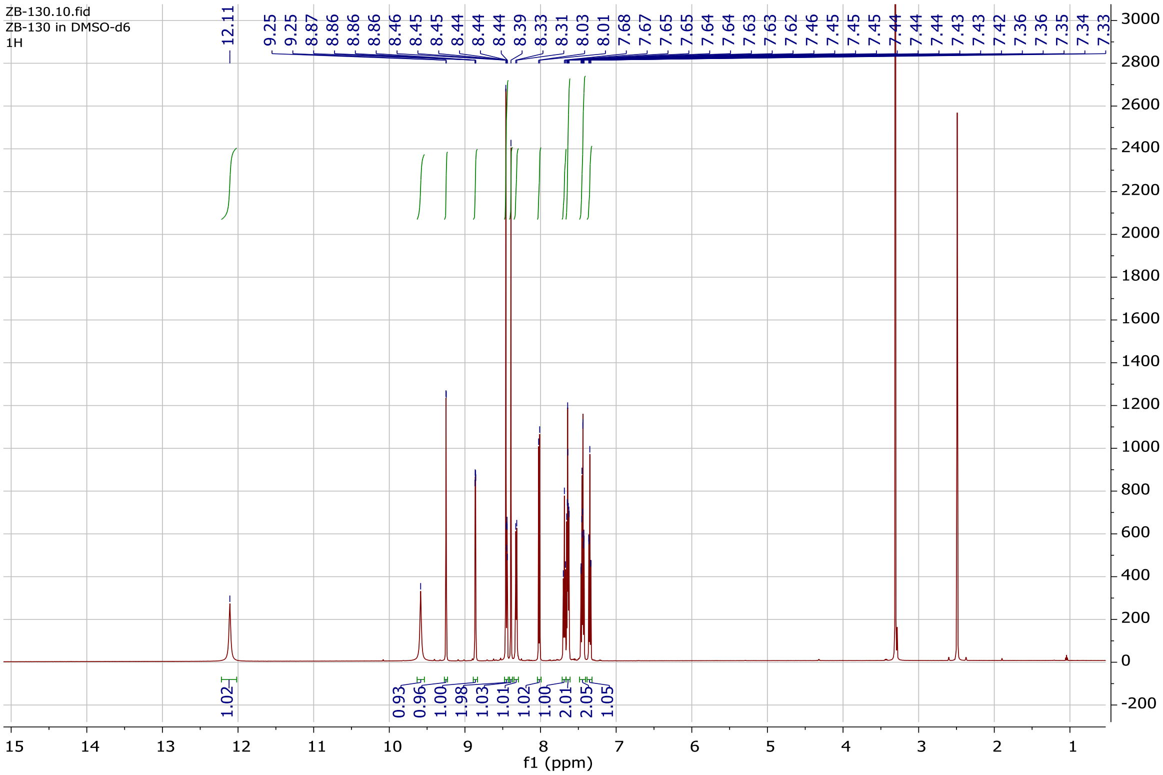

## $^{13}$CNMR of 5h

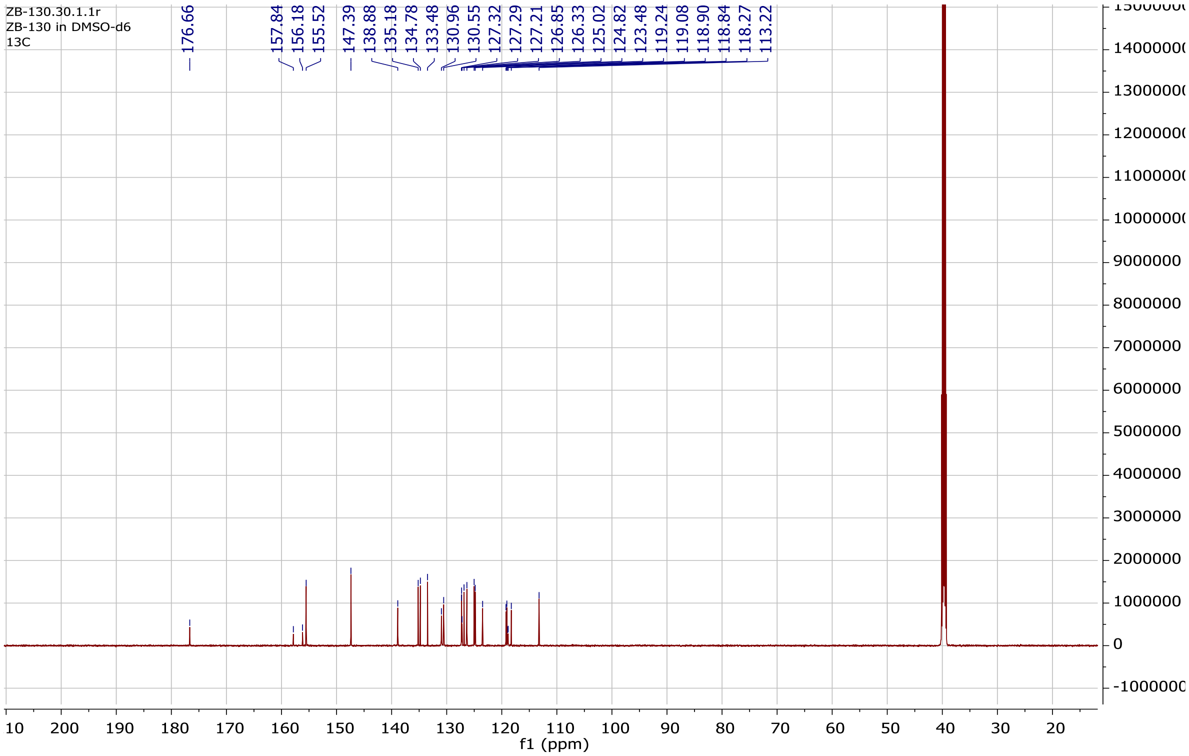


## HRMS of 5h

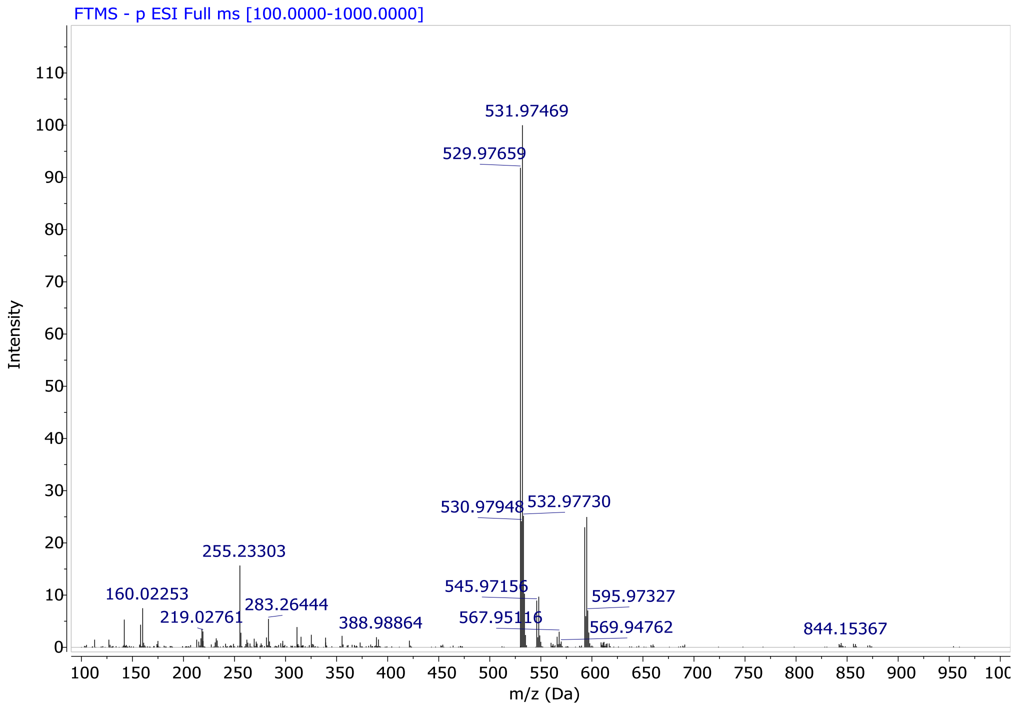

## 1HNMR of 5i

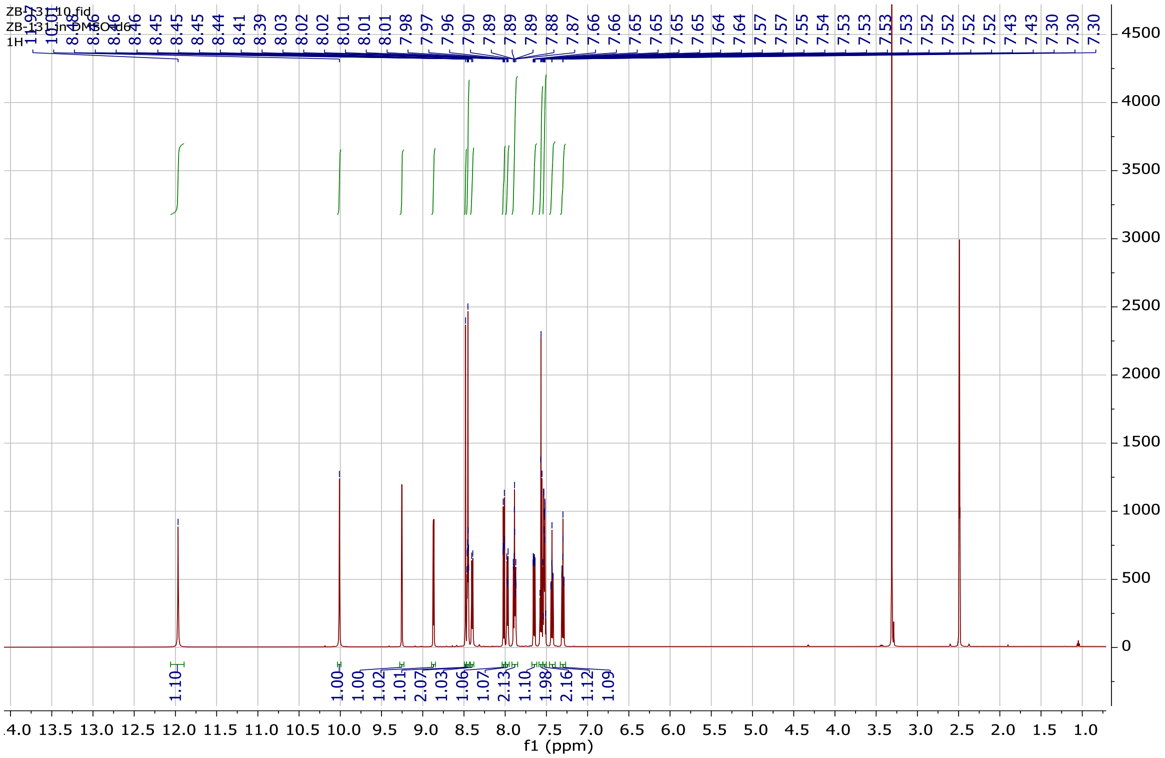


## 13C NMR of 5i

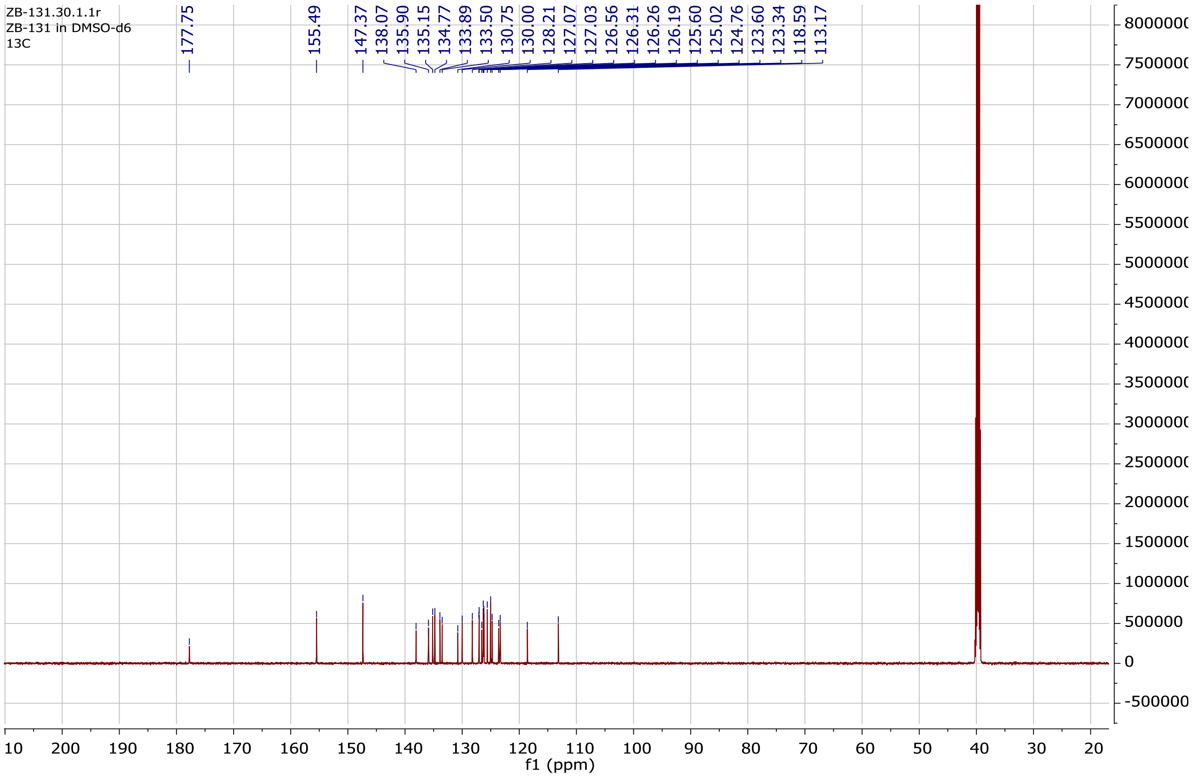

## HRMS of 5i

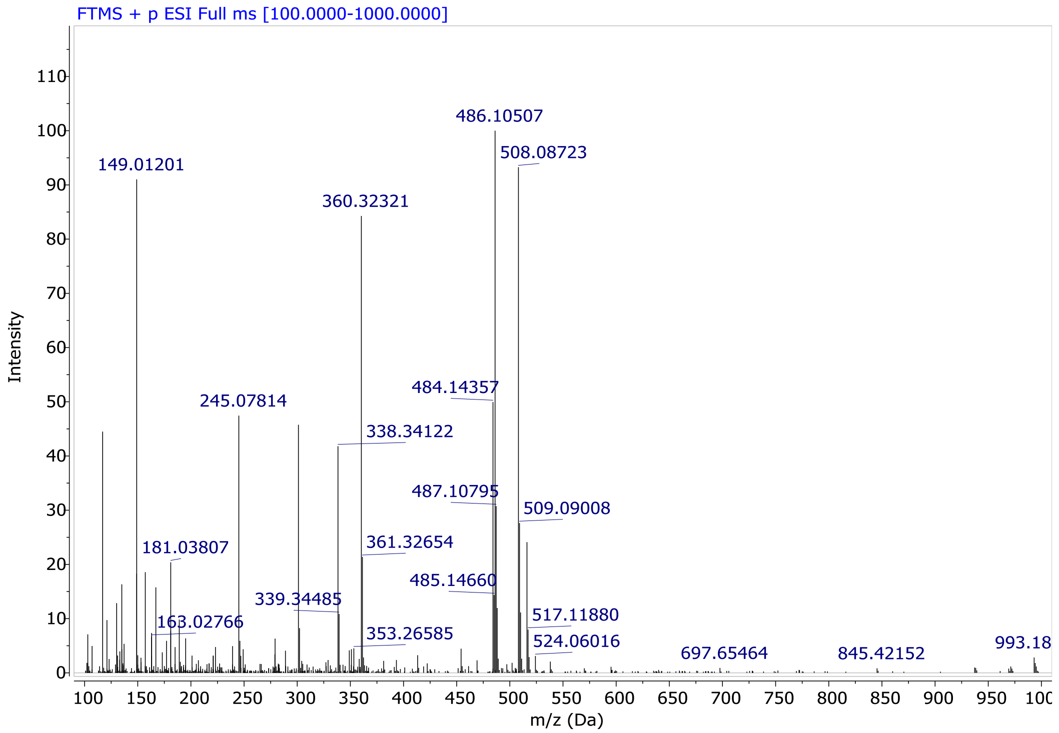


## $^{1}$HNMR of 5j

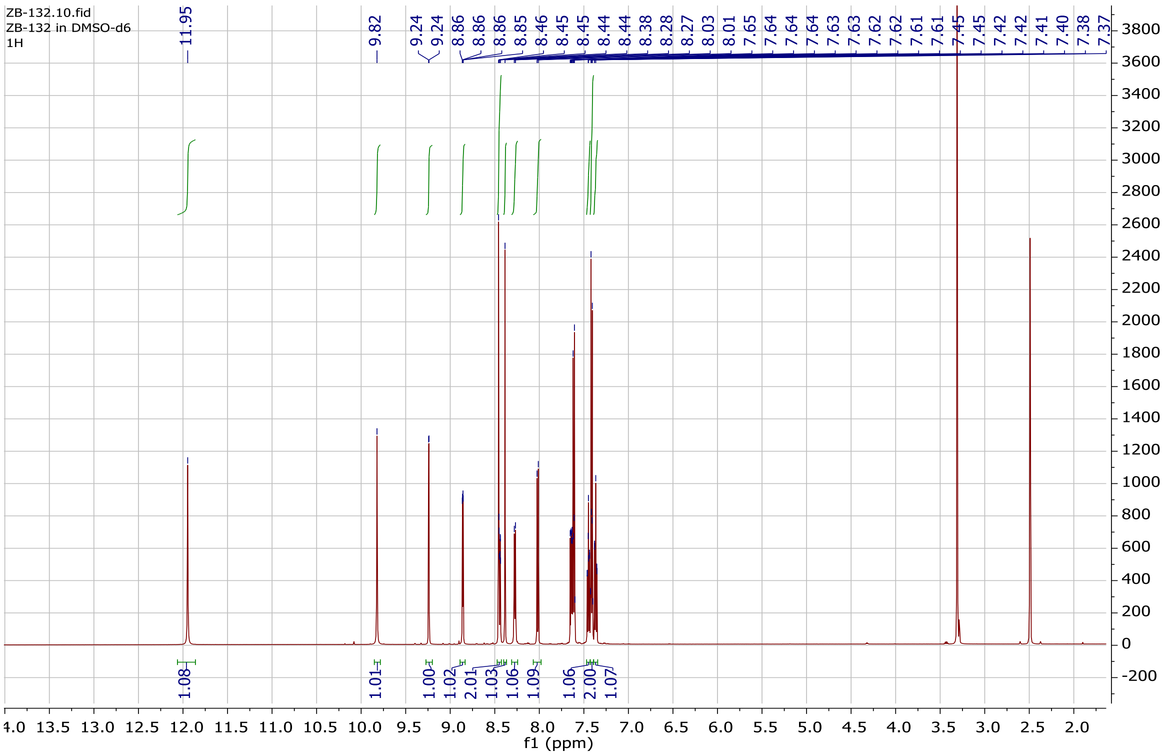

## $^{13}C$ NMR of 5j

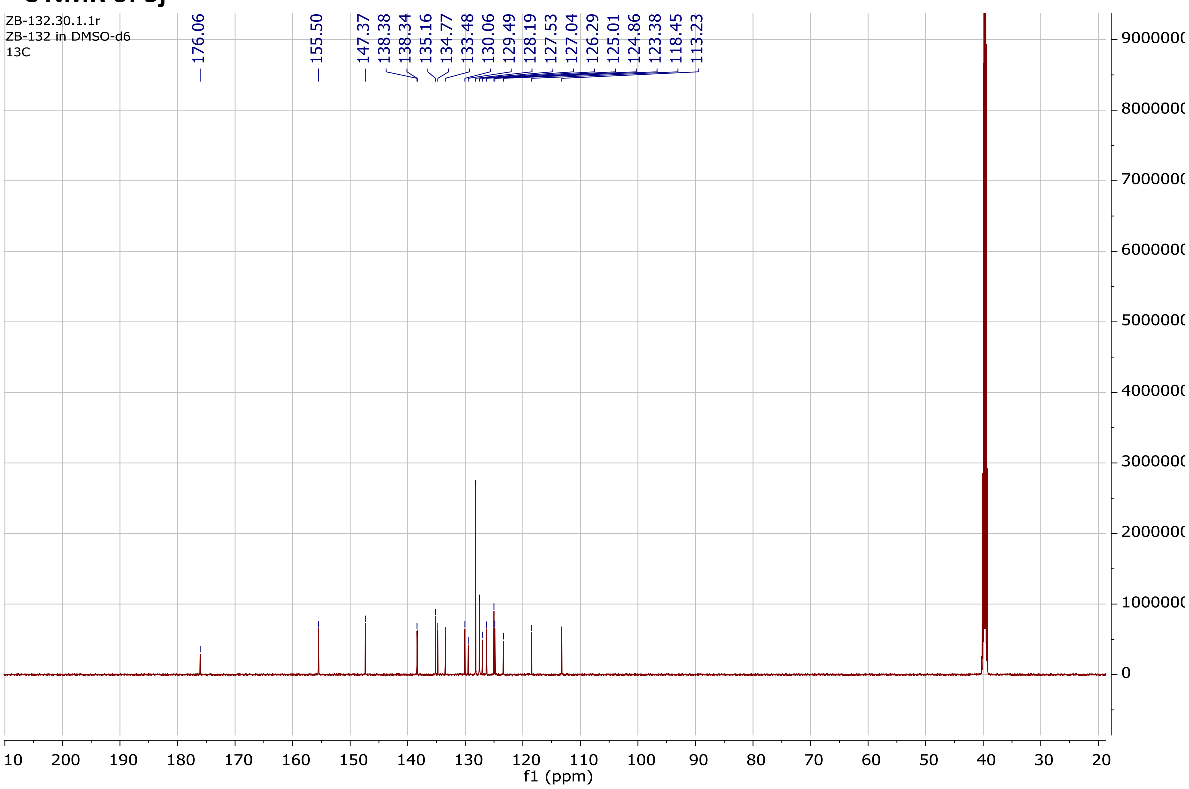


## HRMS of 5j

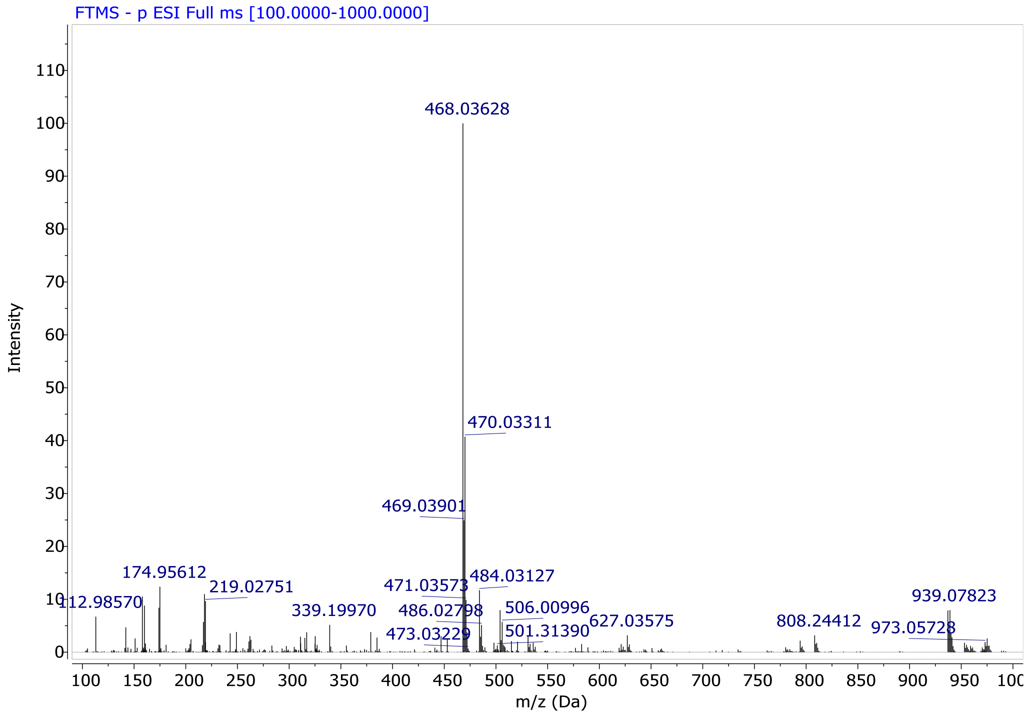

## $^{1}$HNMR of 5k

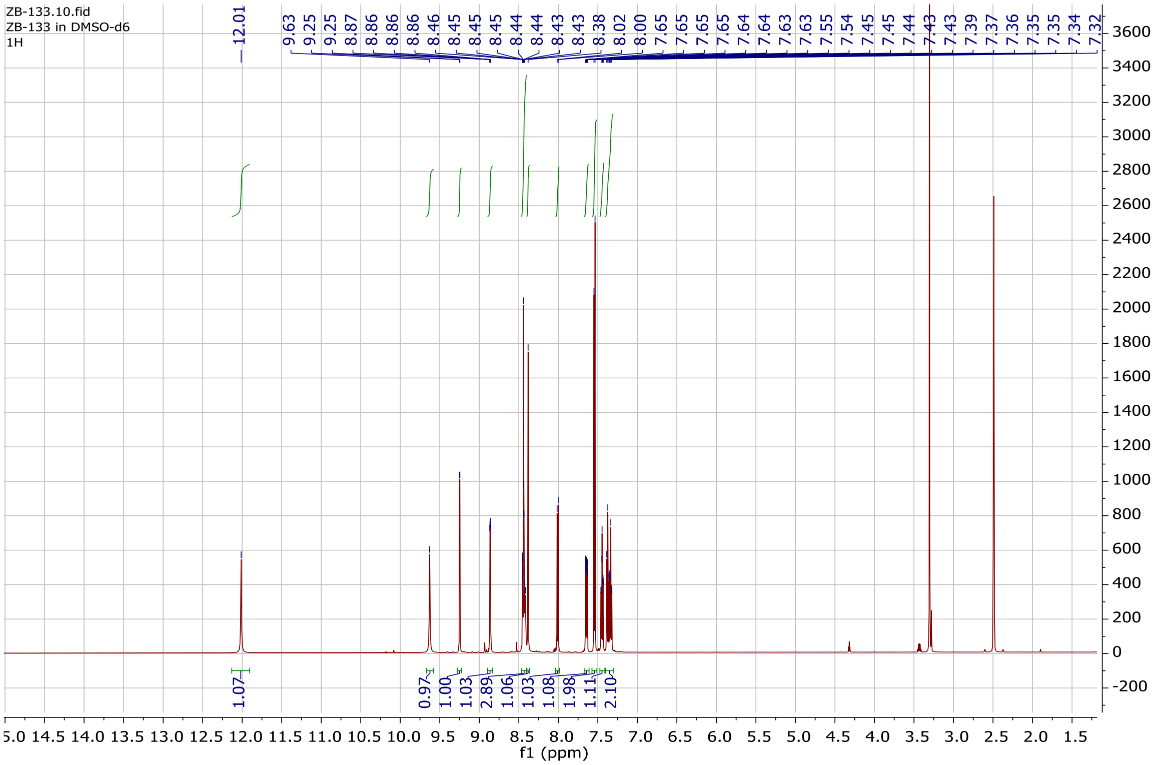


## $^{13}$C NMR of 5k

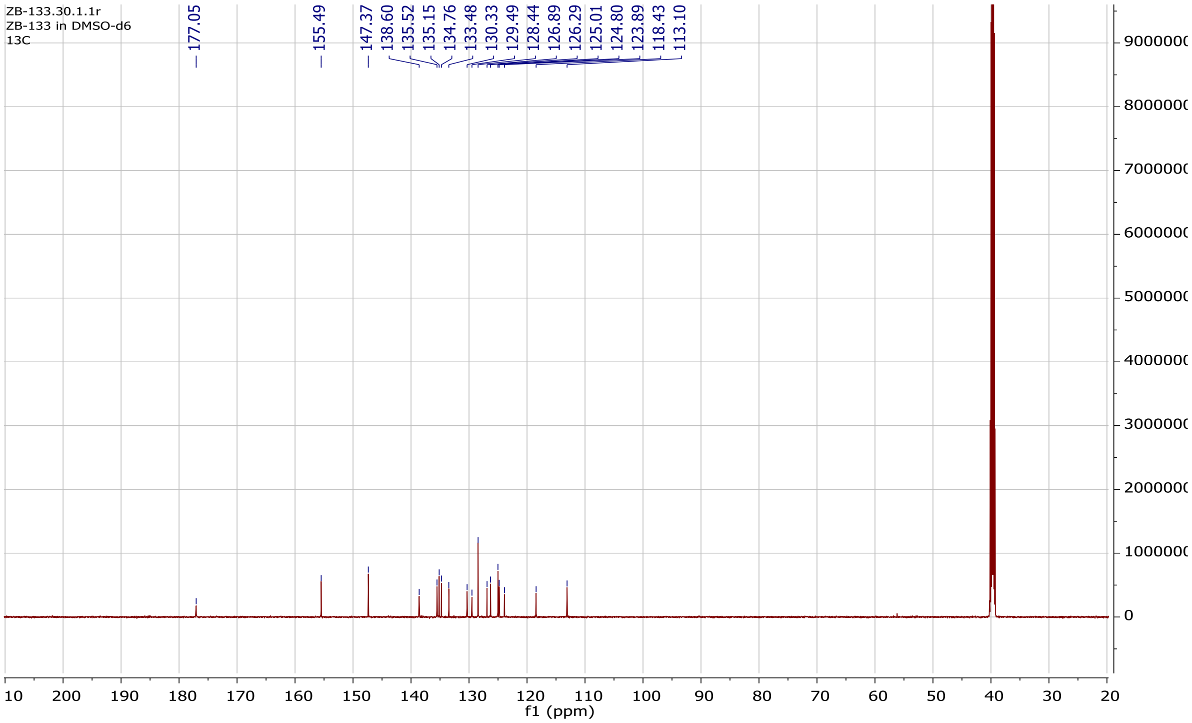

## HRMS of 5k

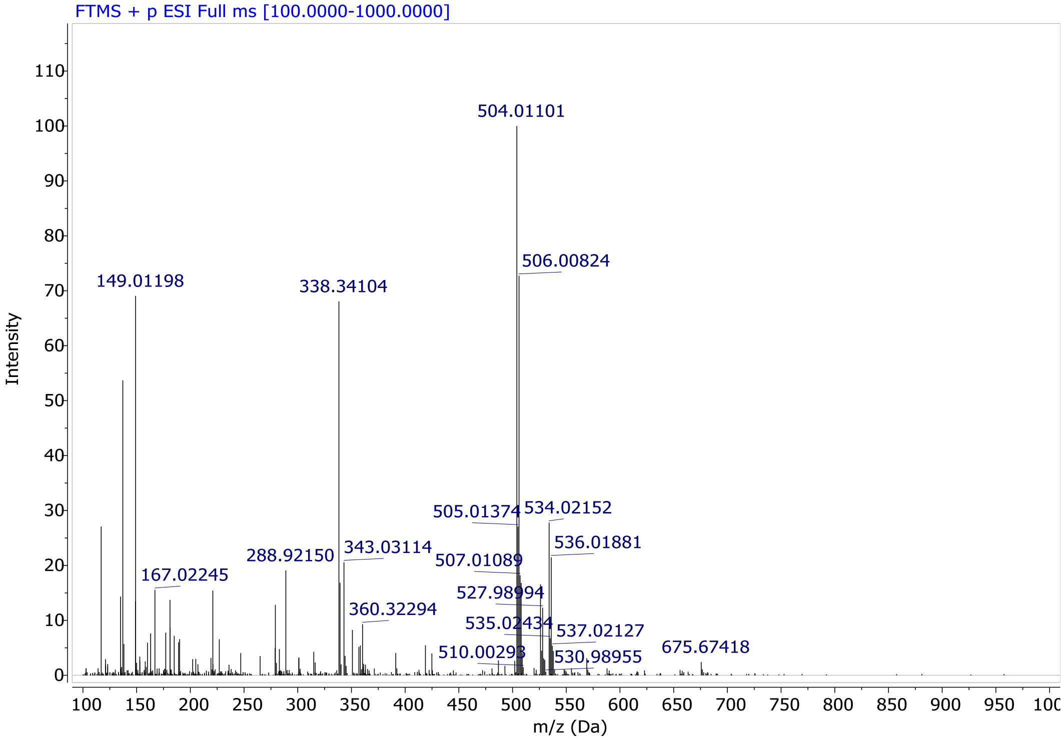


## $^{1}$HNMR of 5l

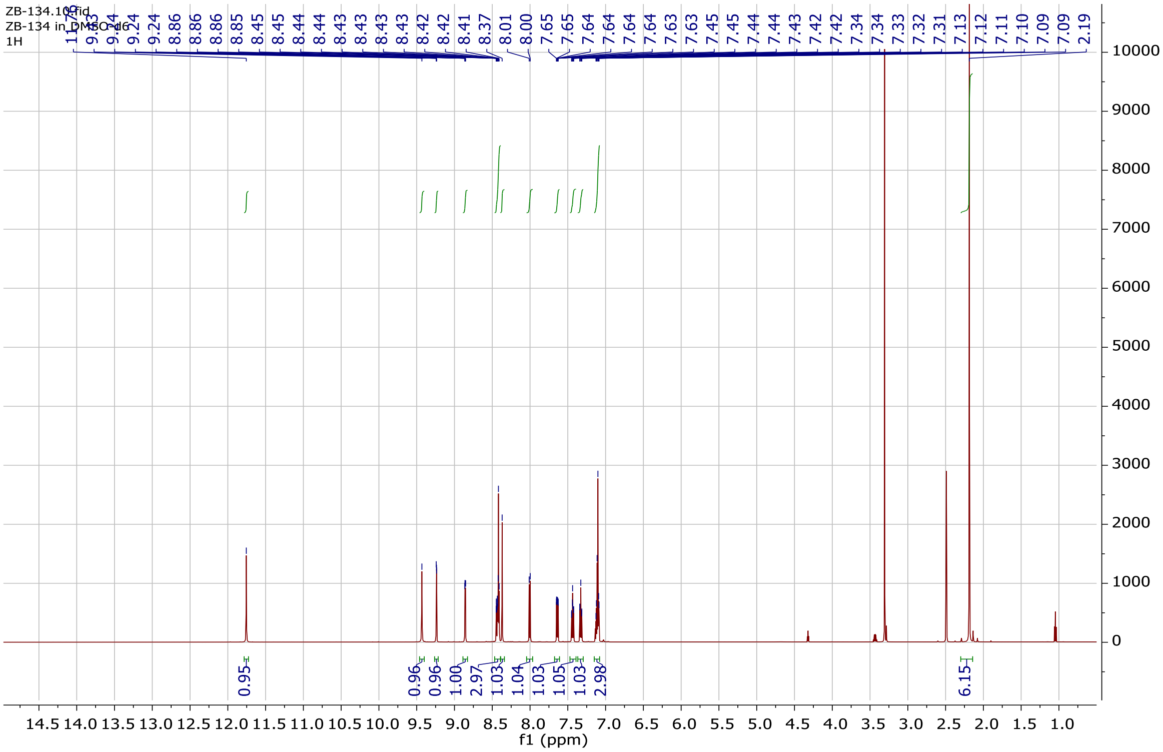

## $^{13}$C NMR of 5l

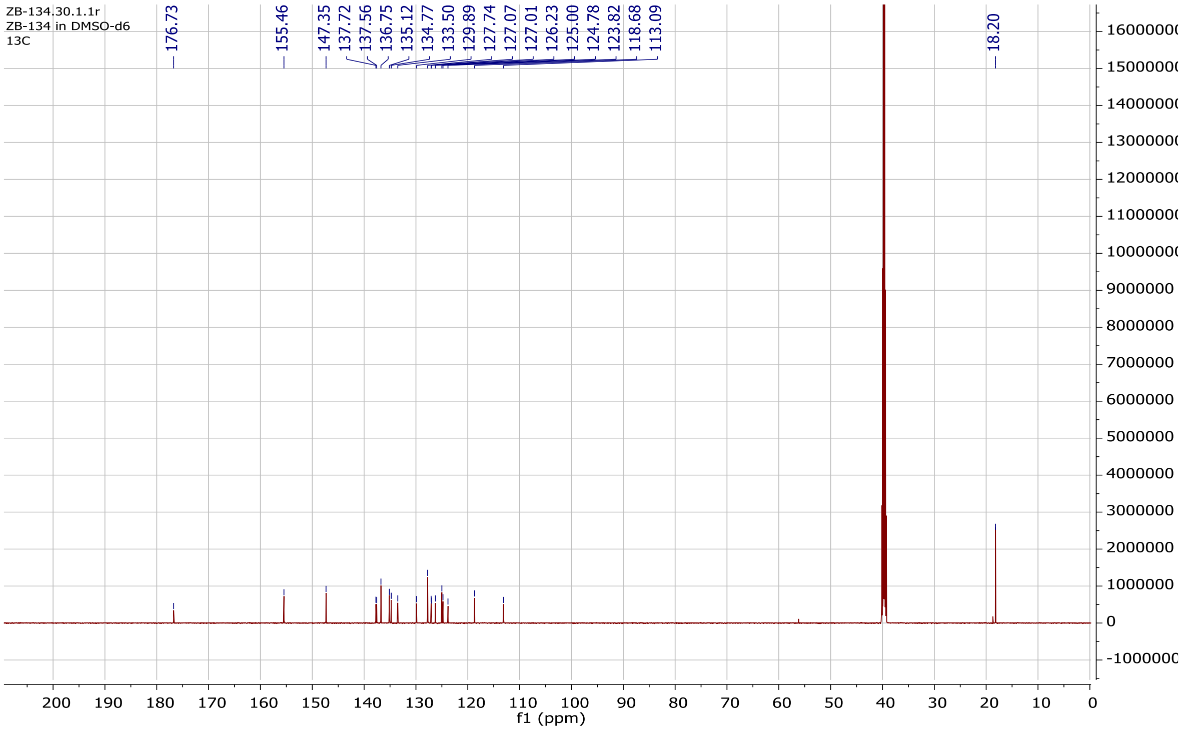


## HRMS of 5l

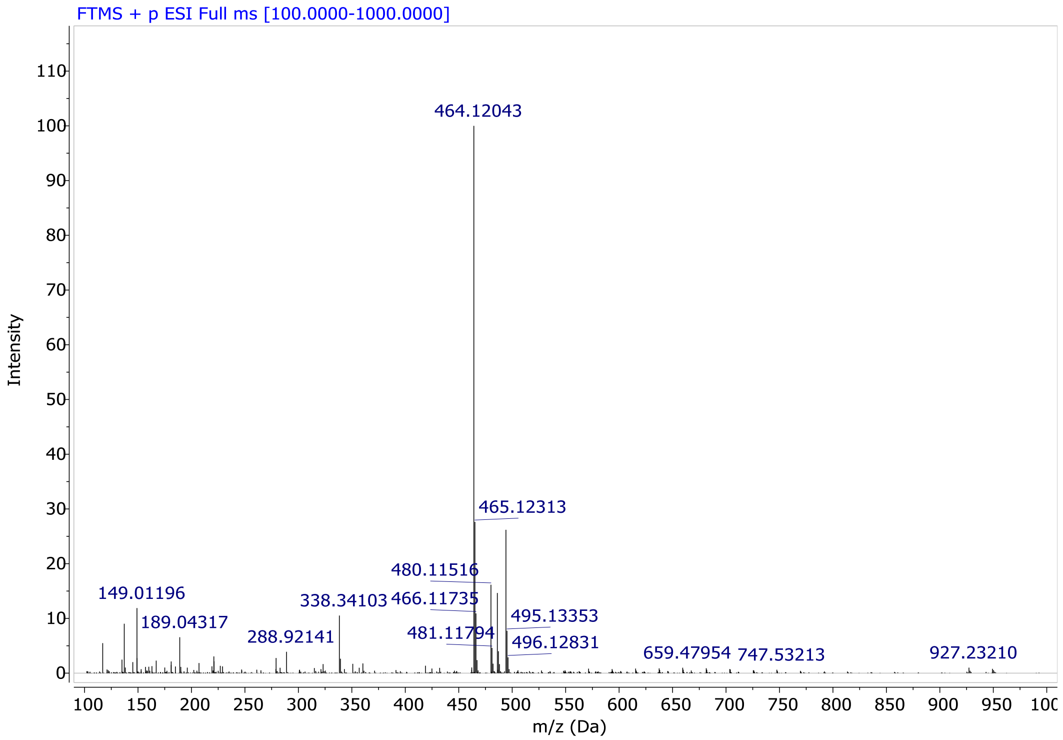

## $^{1}$HNMR of 5m

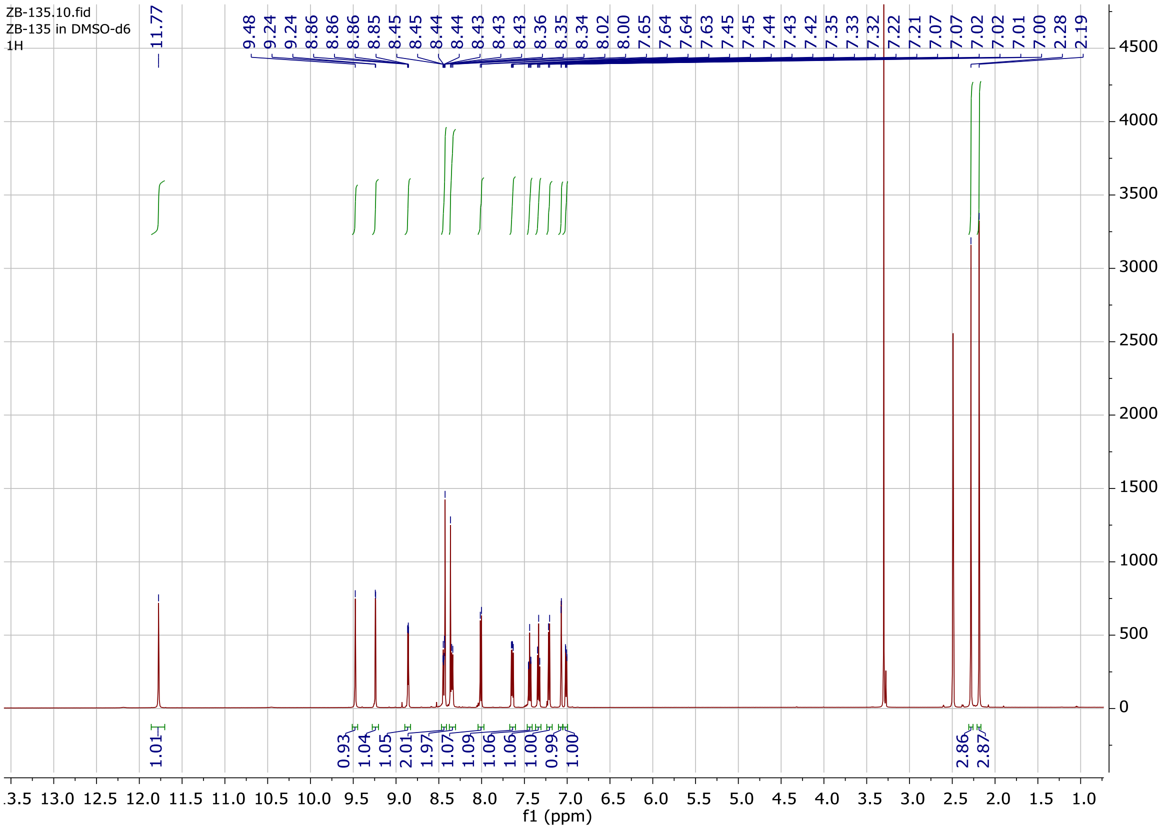


## $^{13}$C NMR of 5m

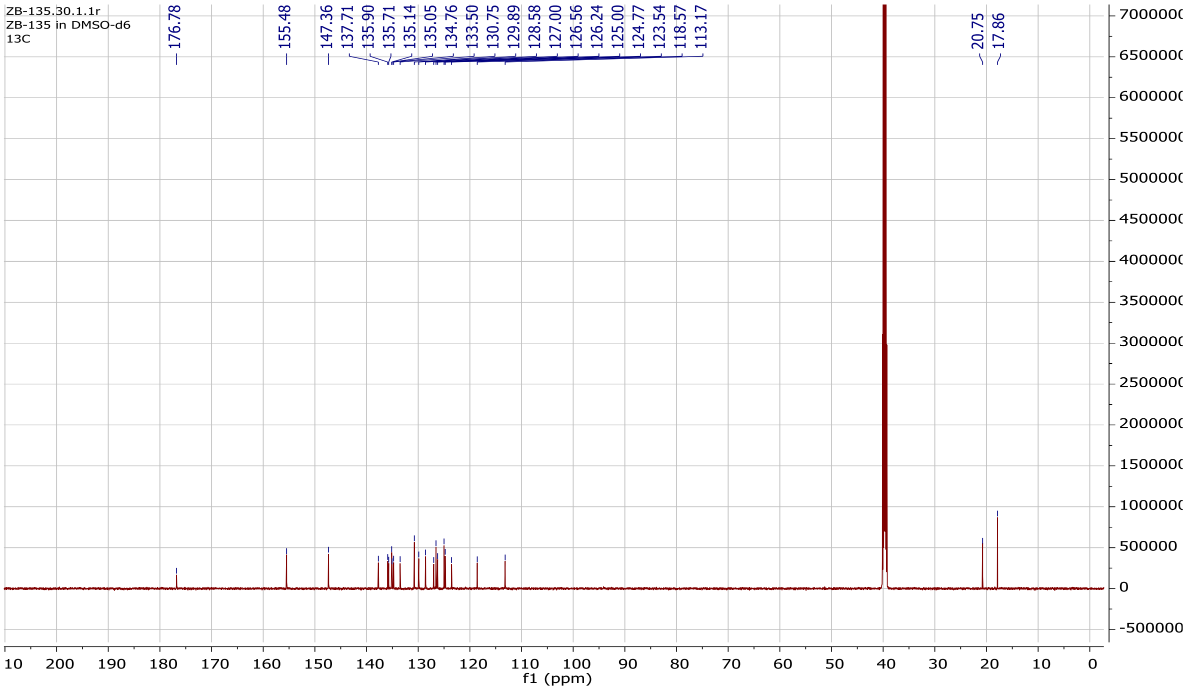

## HRMS of 5m

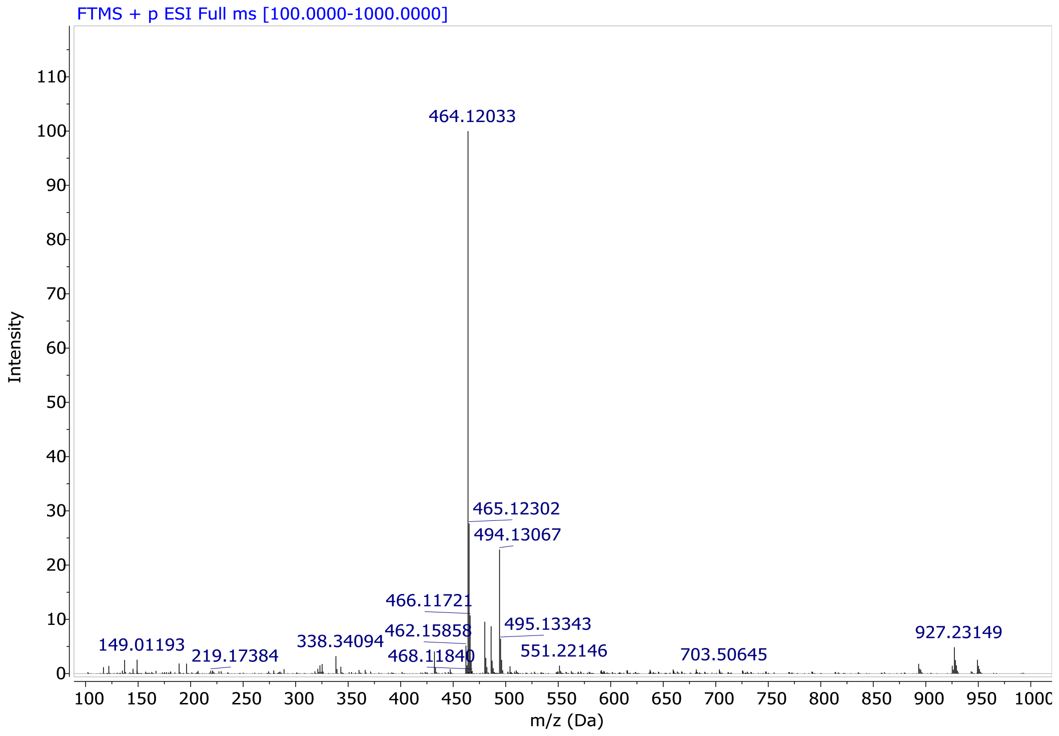


## $^{1}$HNMR of 5n

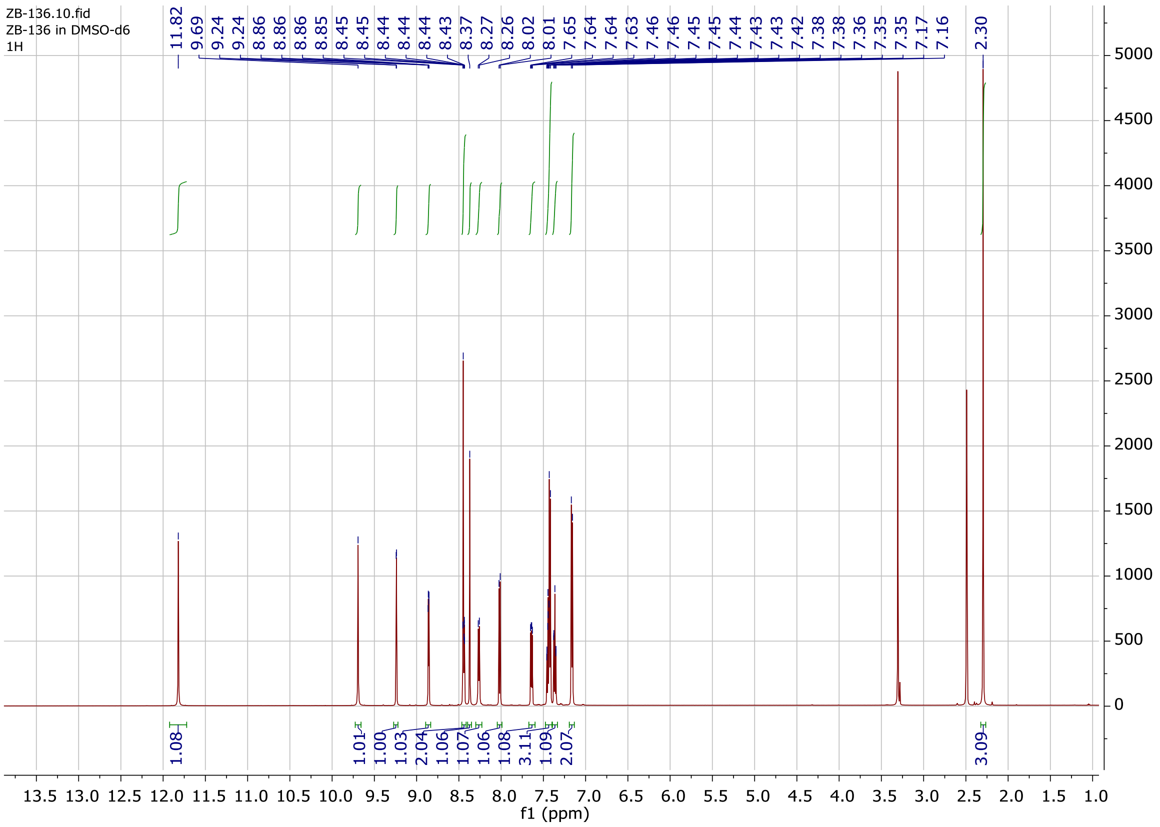

## $^{13}$C NMR of 5n

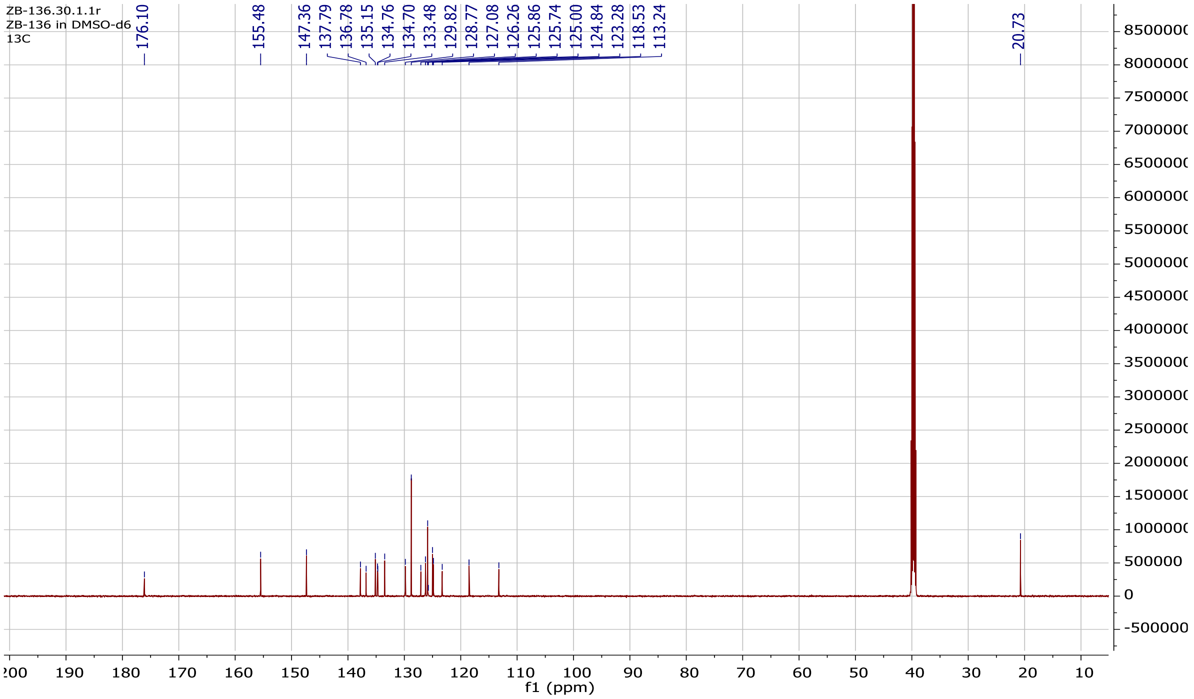


## HRMS of 5n

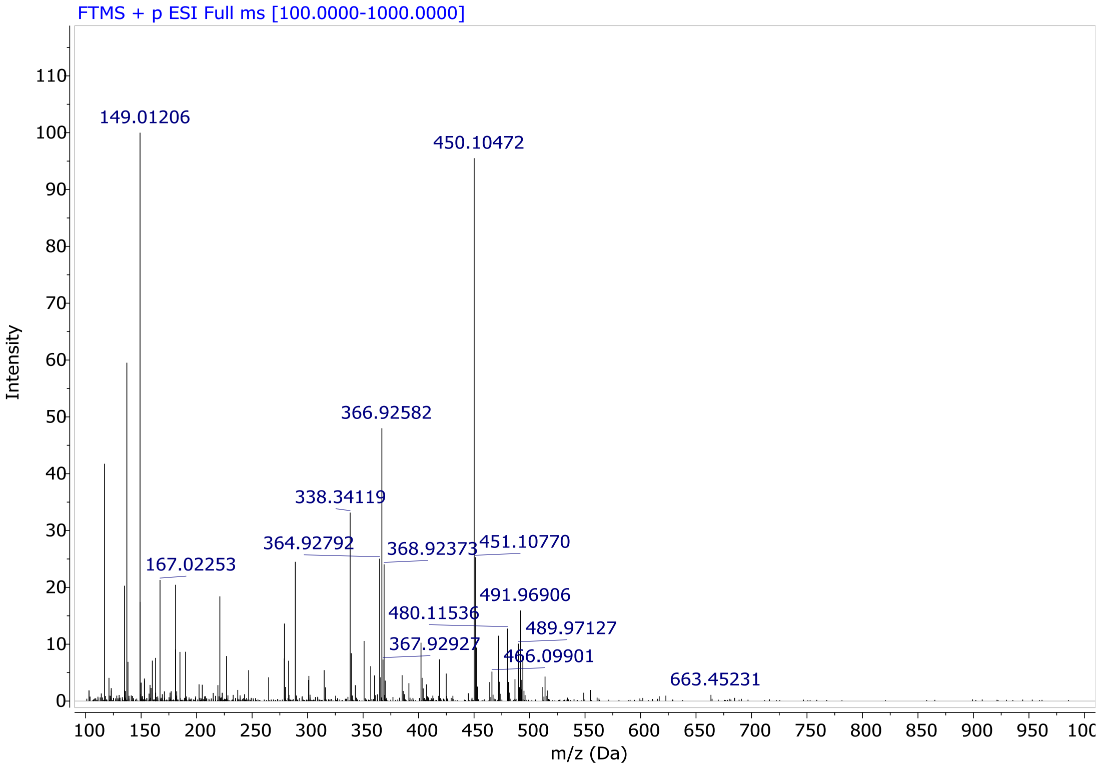

## $^1$HNMR of 5o

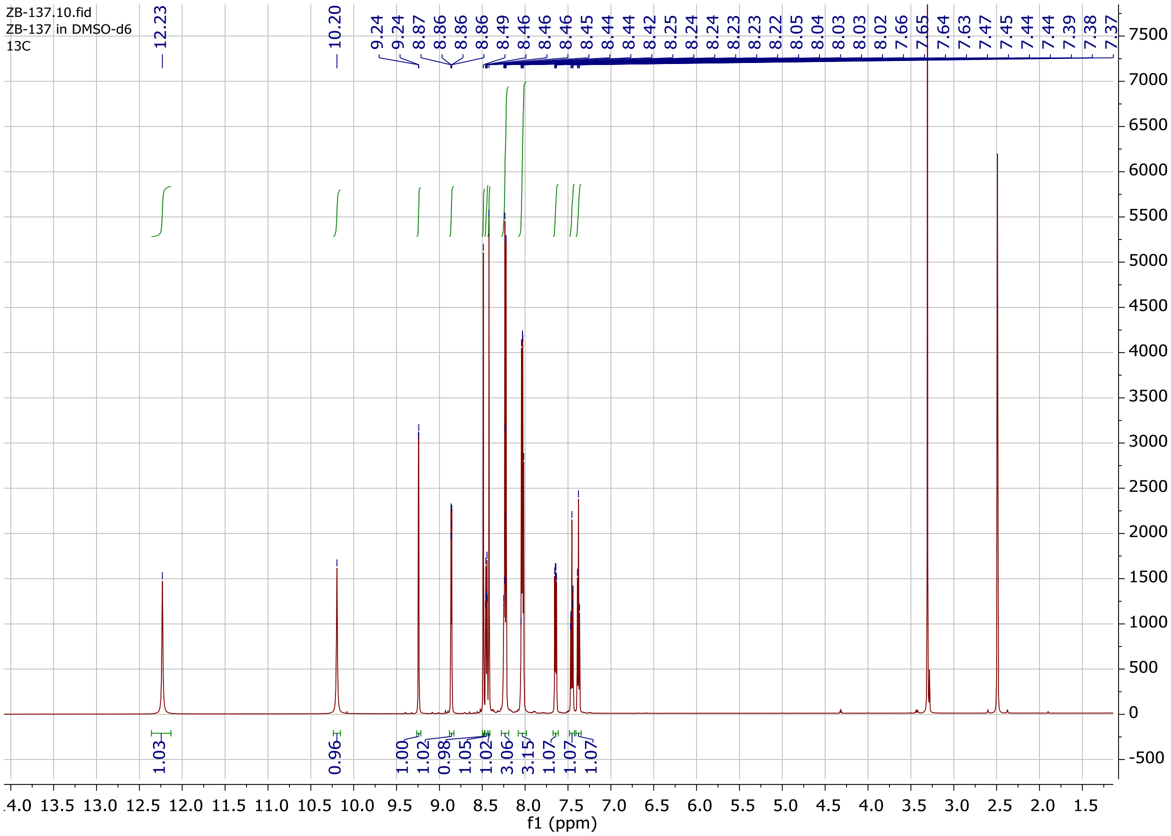

## $^{13}$C NMR of 5o

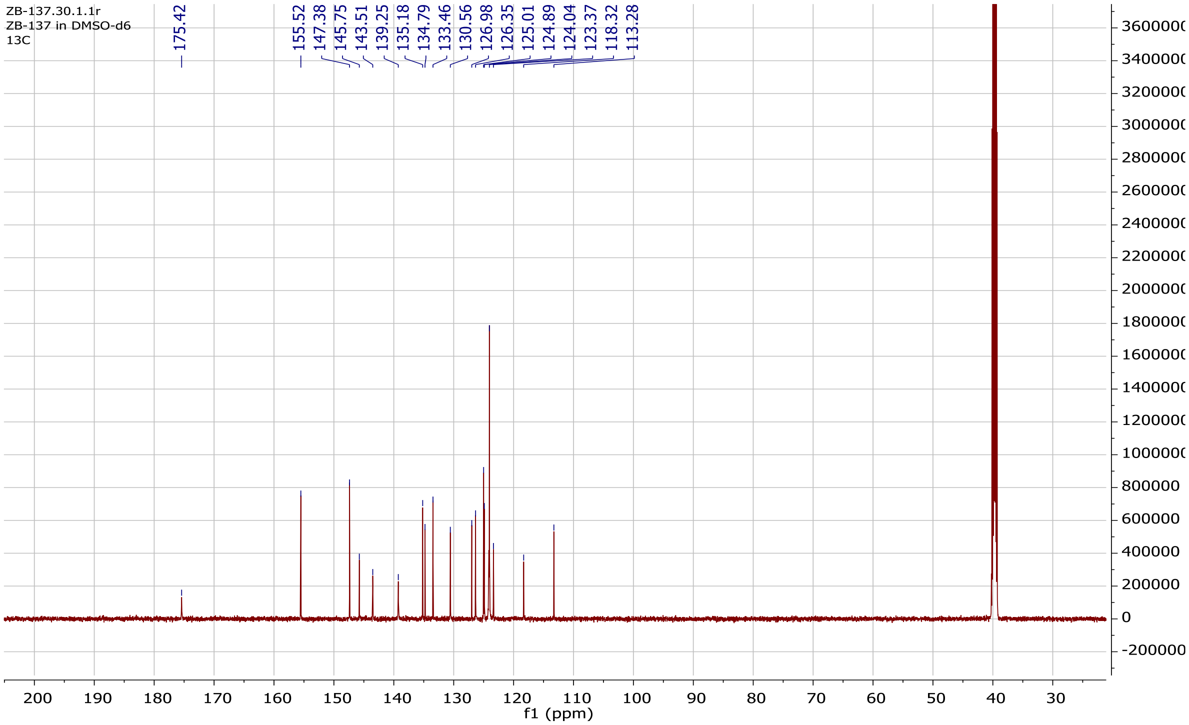

## HRMS of 5o

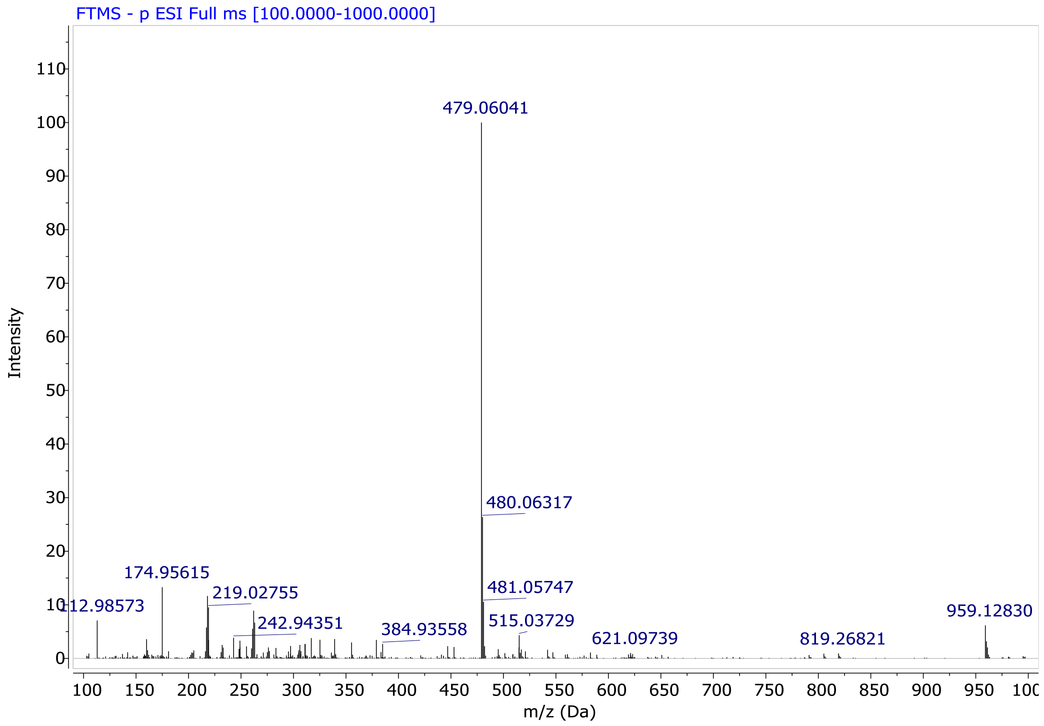


## $^{1}$HNMR of 5p

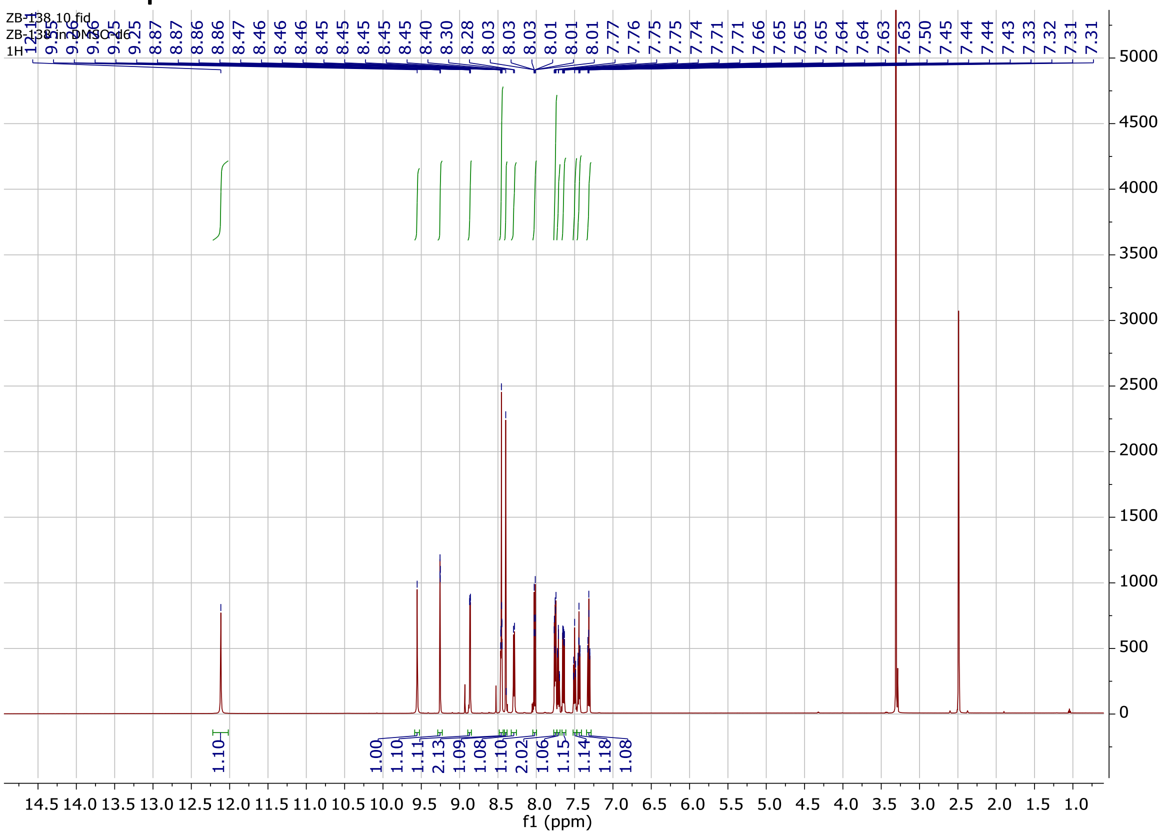

**$^{13}$C NMR of 5p**

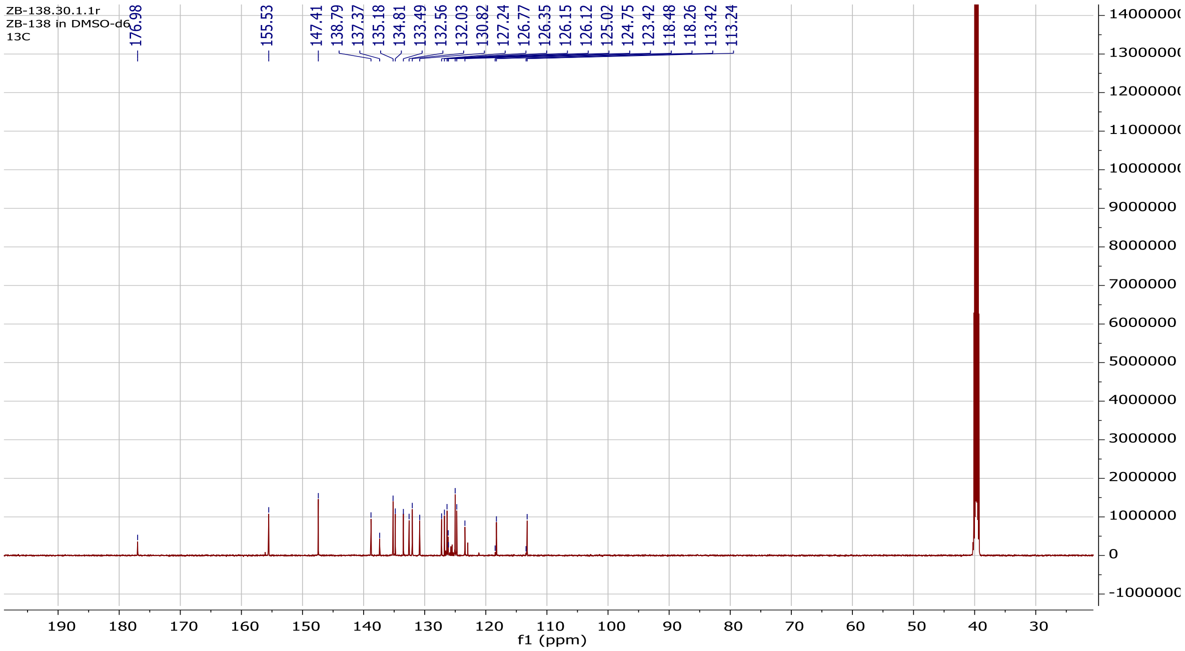


**HRMS of 5p**

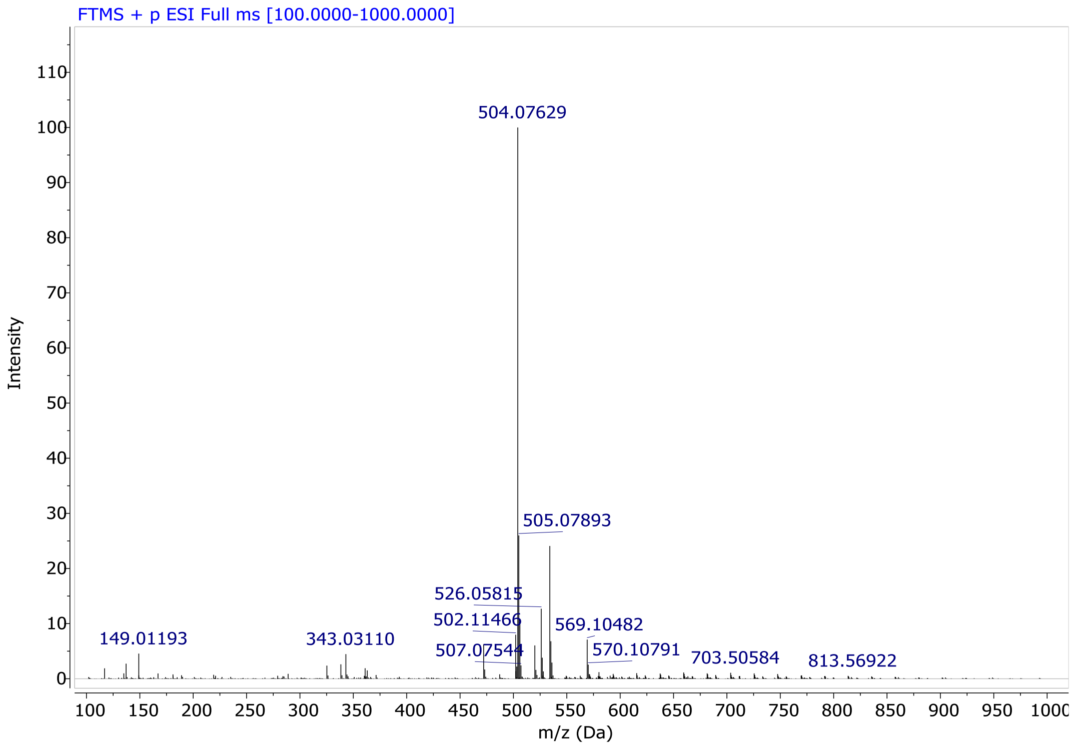

## $^{1}$HNMR of 5q

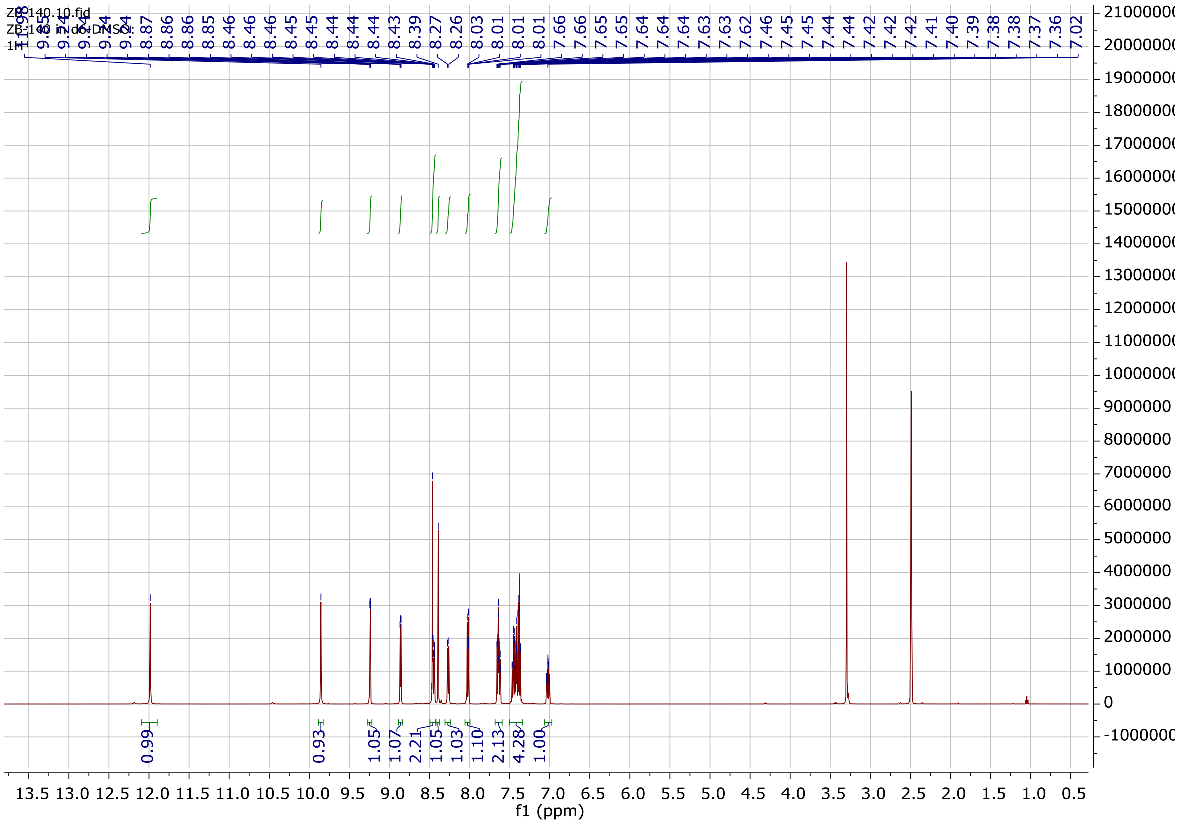


## $^{13}$C NMR of 5q

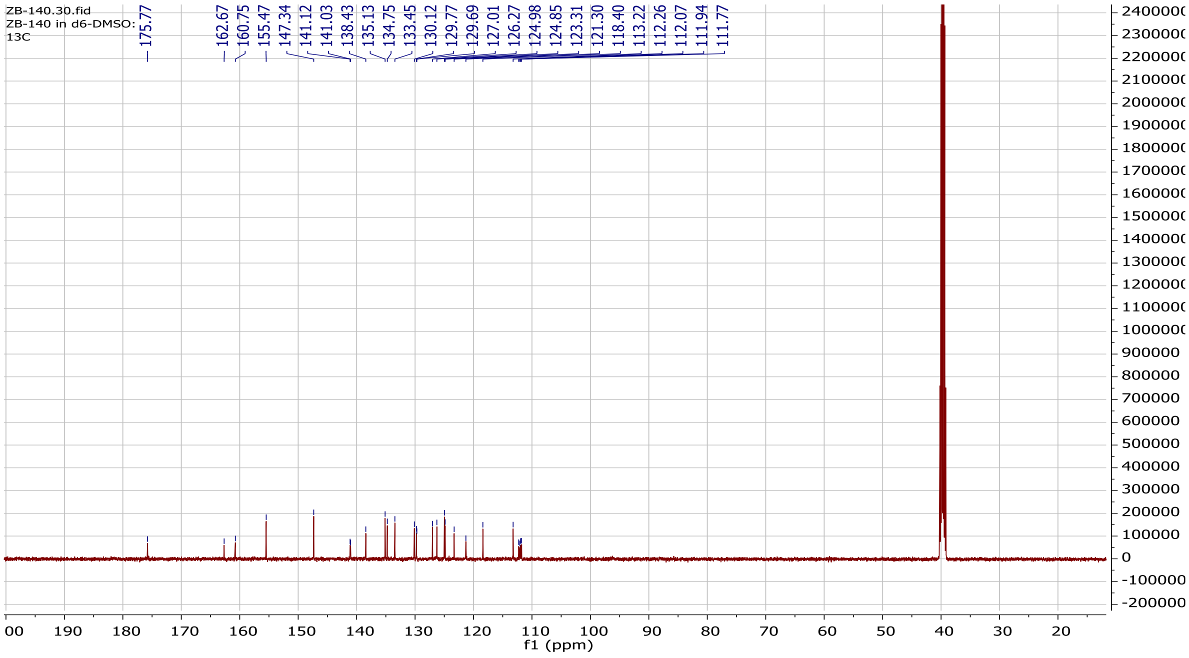

## HRMS of 5q

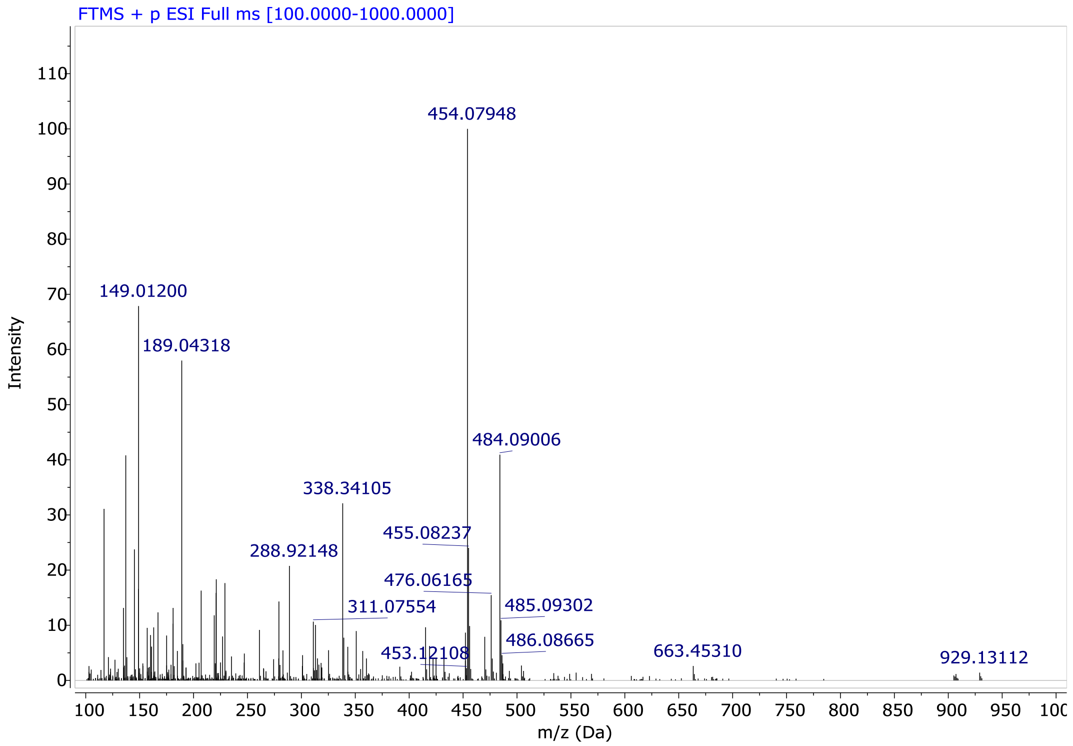


## $^{1}$HNMR of 5r

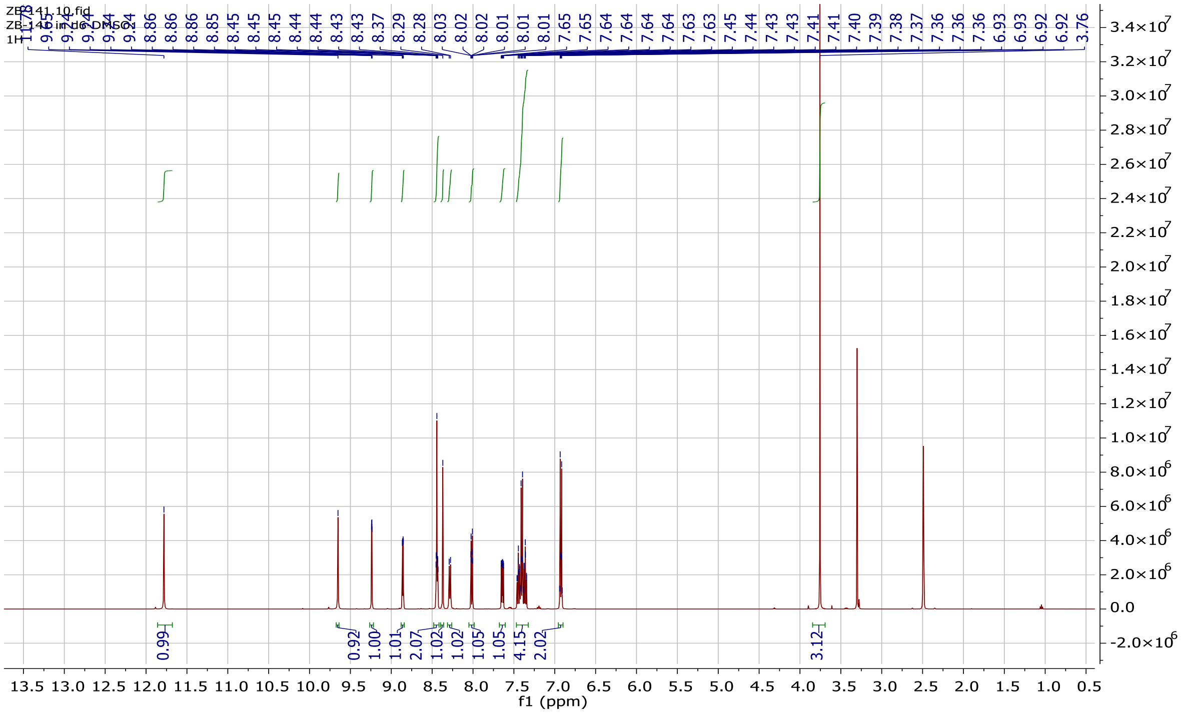

## $^{13}$C NMR of 5r

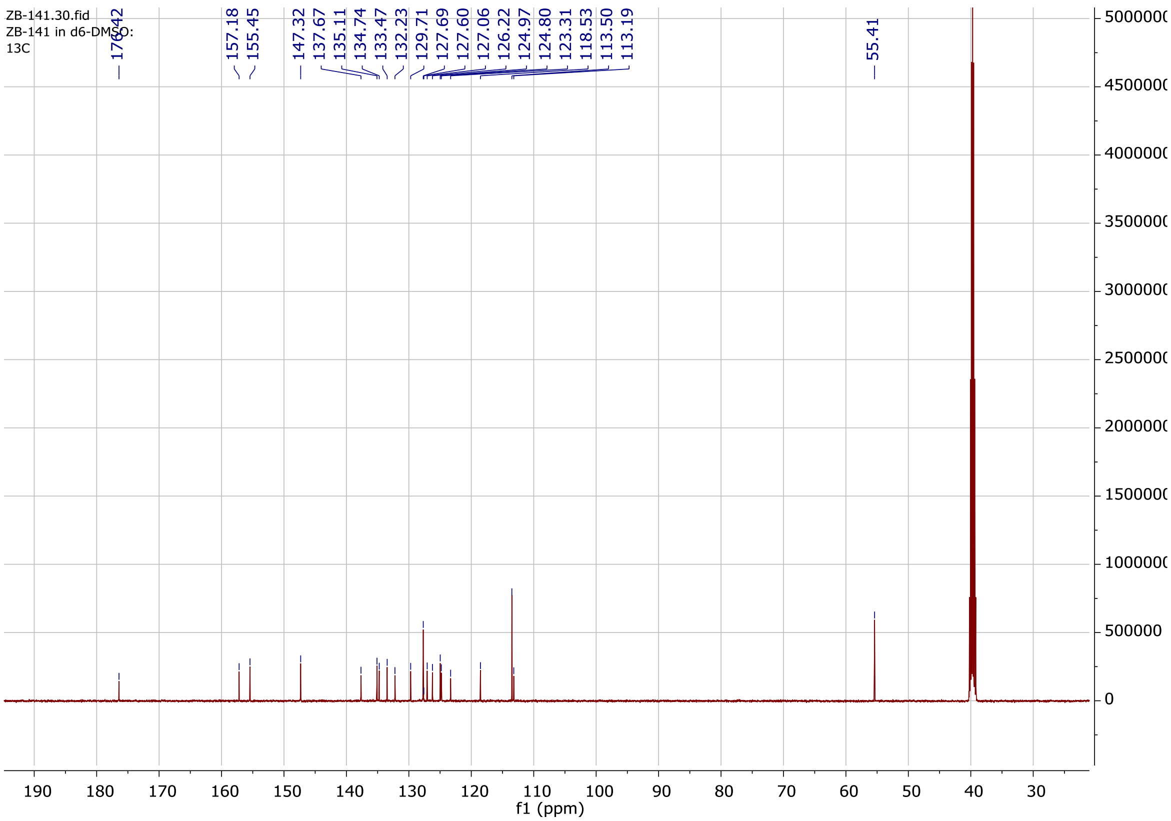


## HRMS of 5r

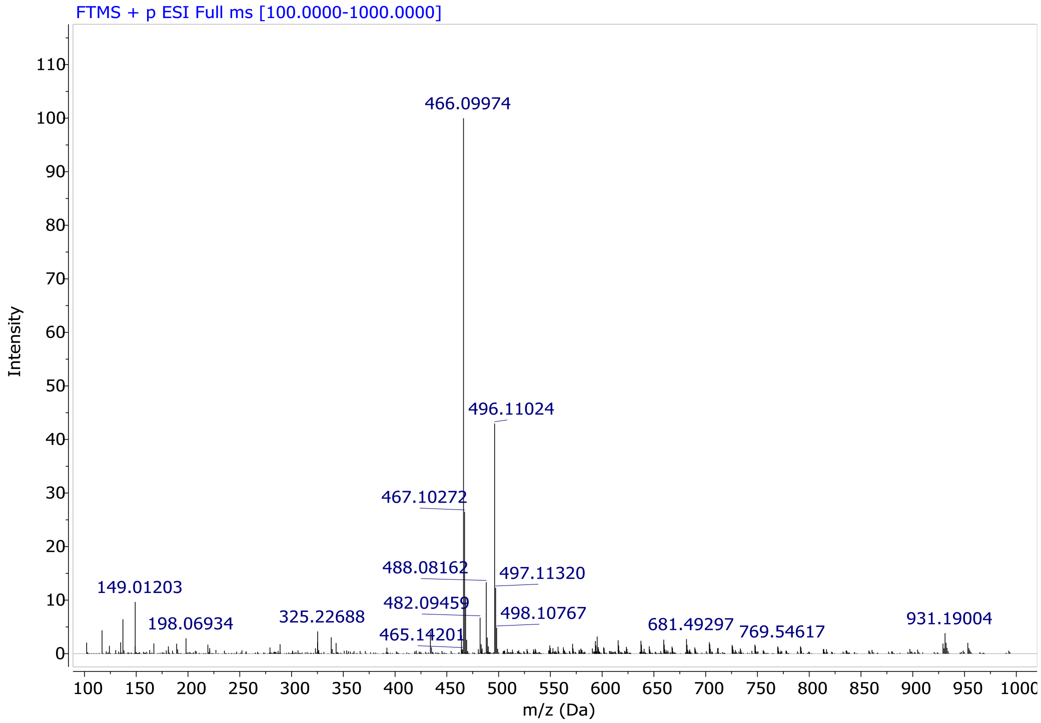

**$IC_{50}$ Graphs of Compounds**

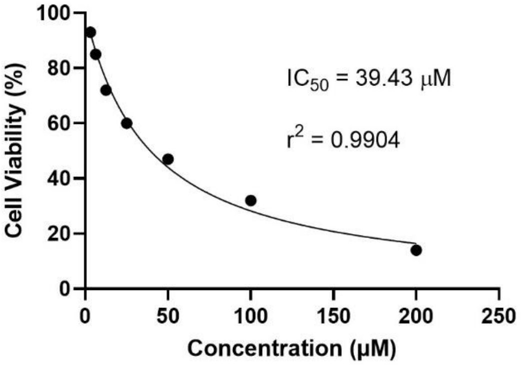


Compound 3 for A549

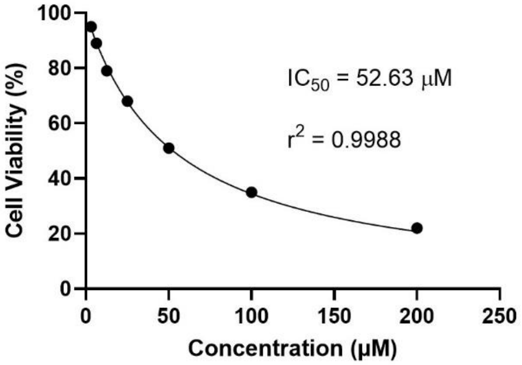


Compound 3 for BEAS-2B

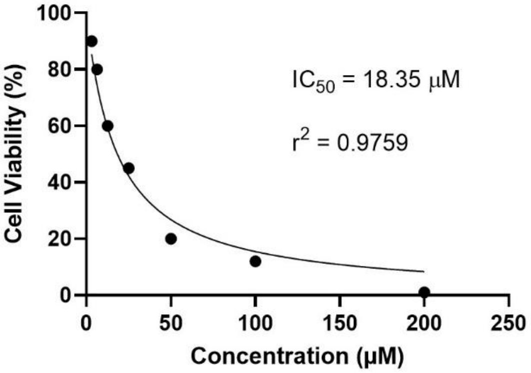


Compound 5a for A549

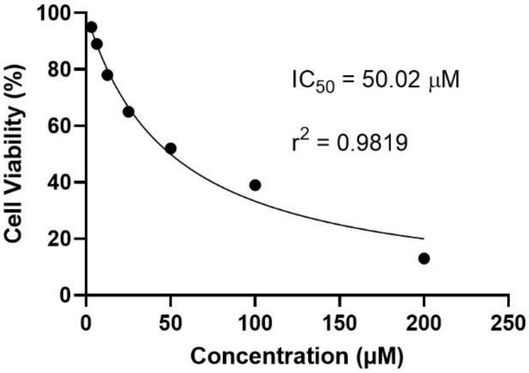


Compound 5a for BEAS-2B

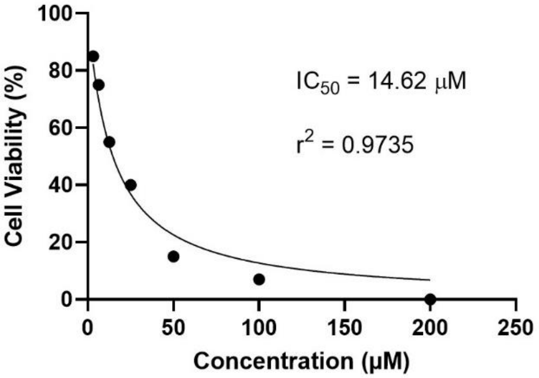


Compound 5b for A549

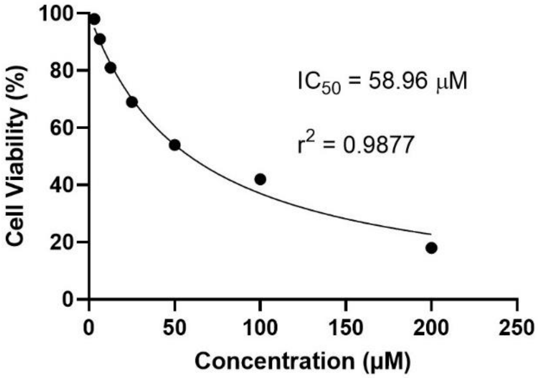


Compound 5b for BEAS-2B

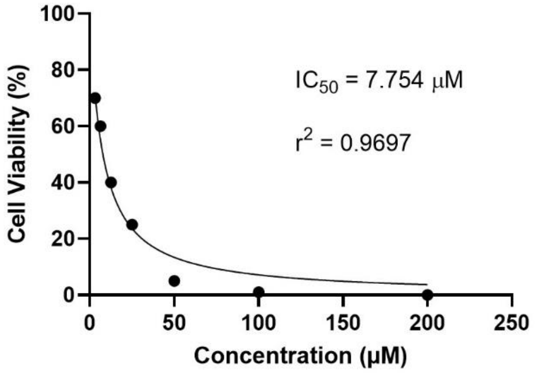


Compound 5c for A549

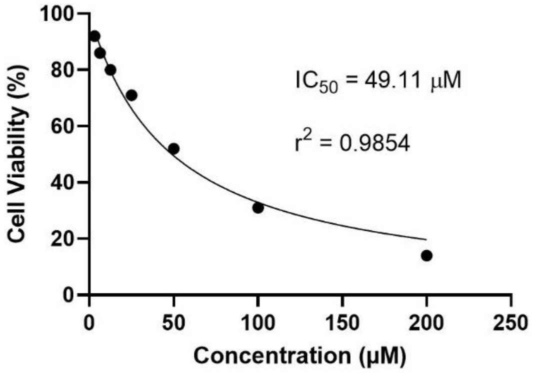


Compound 5c for BEAS-2B

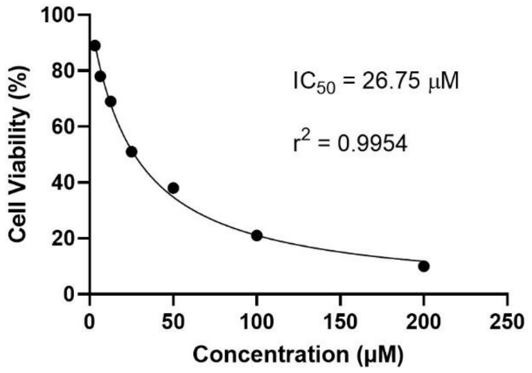


Compound 5d for A549

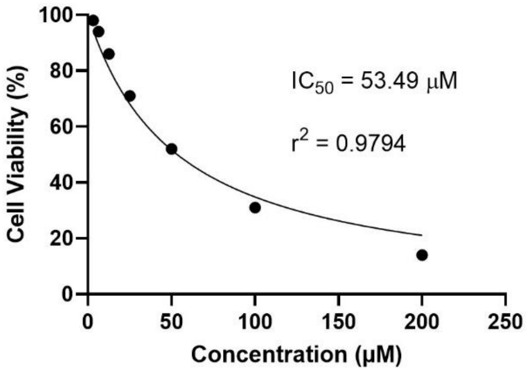


Compound 5d for BEAS-2B

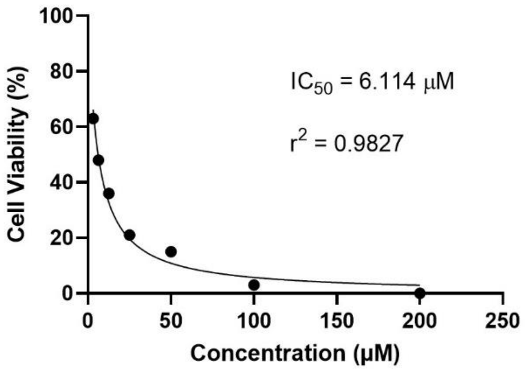


Compound 5e for A549

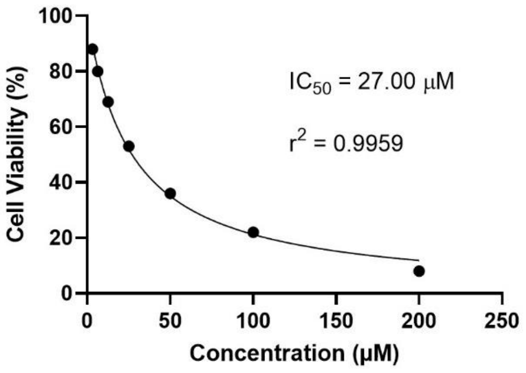


Compound 5e for BEAS-2B

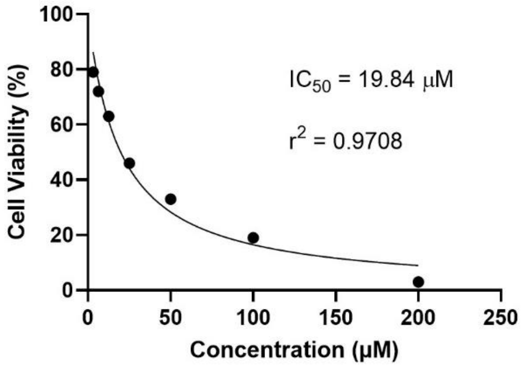


Compound 5f for A549

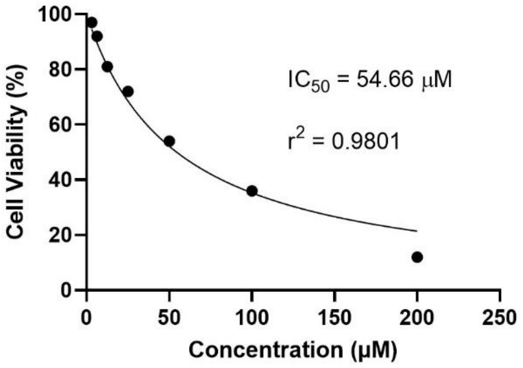


Compound 5f for BEAS-2B

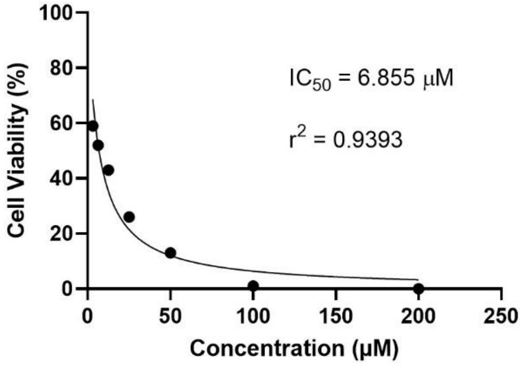


Compound 5g for A549

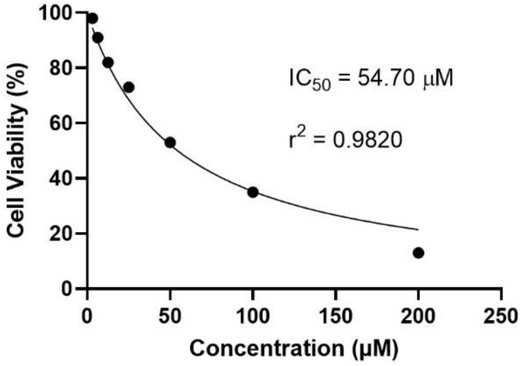


Compound 5g for BEAS-2B

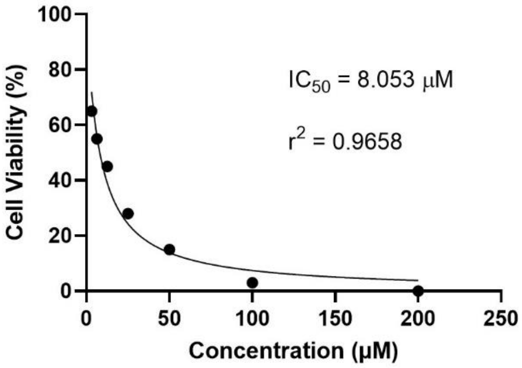


Compound 5h for A549

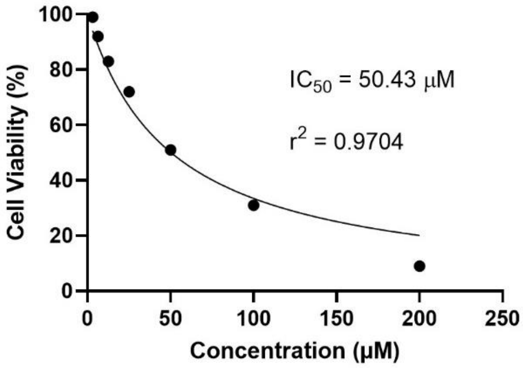


Compound 5h for BEAS-2B

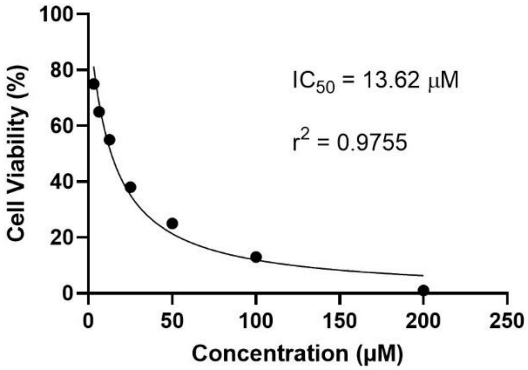


Compound 5i for A549

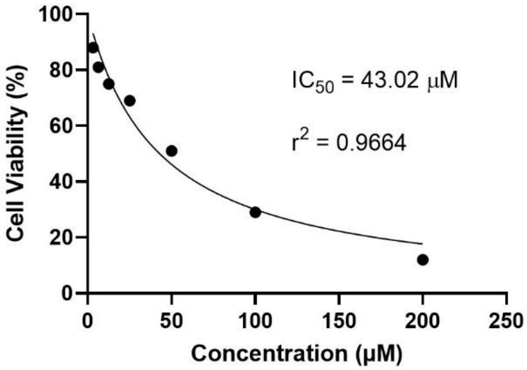


Compound 5i for BEAS-2B

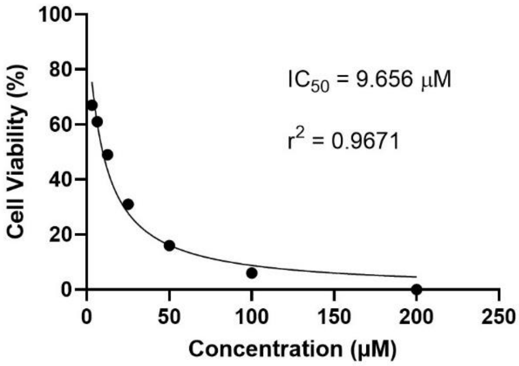


Compound 5j for A549

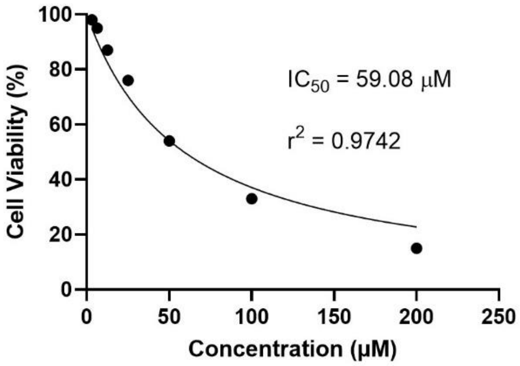


Compound 5j for BEAS-2B

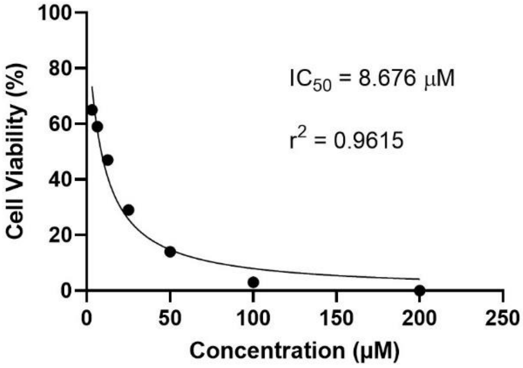


Compound 5k for A549

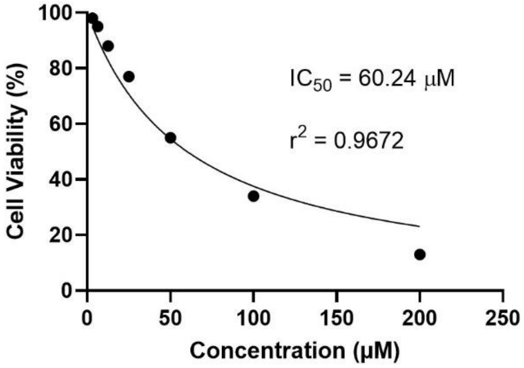


Compound 5k for BEAS-2B

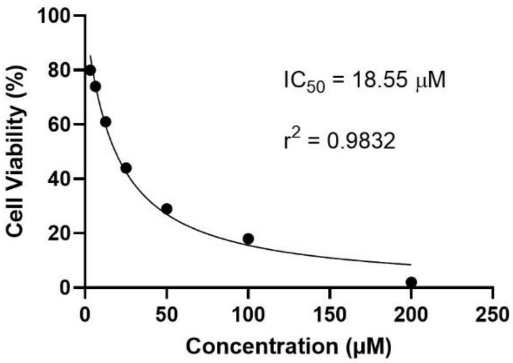


Compound 5l for A549

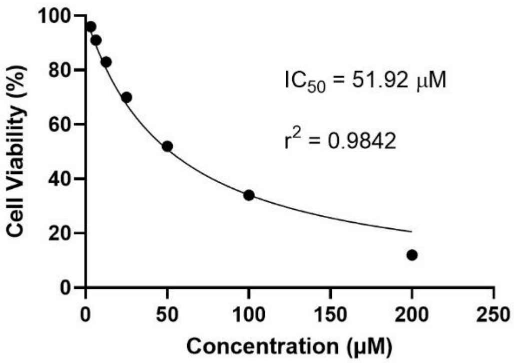


Compound 5l for BEAS-2B

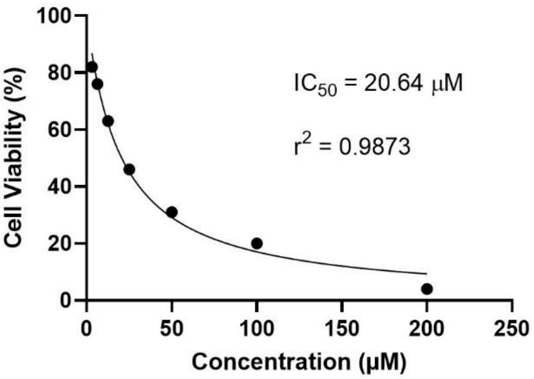


Compound 5m for A549

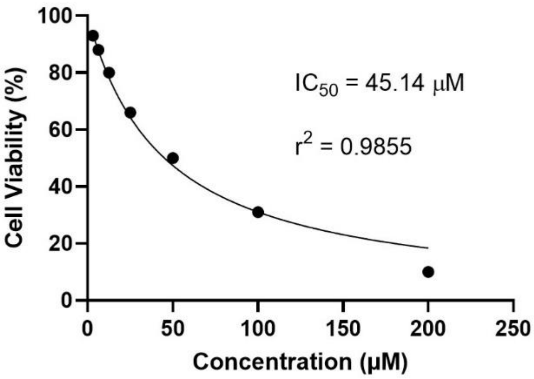


Compound 5m for BEAS-2B

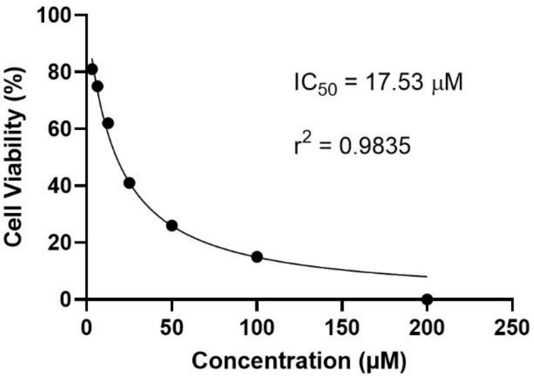


Compound 5n for A549

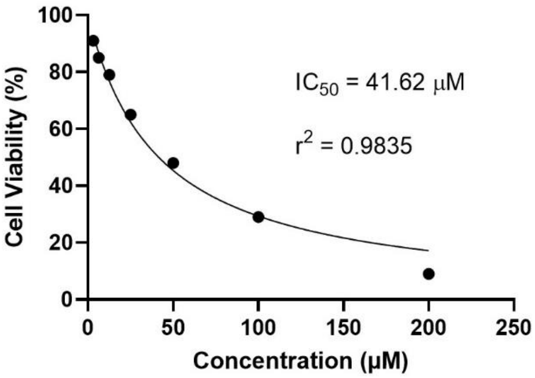


Compound 5n for BEAS-2B

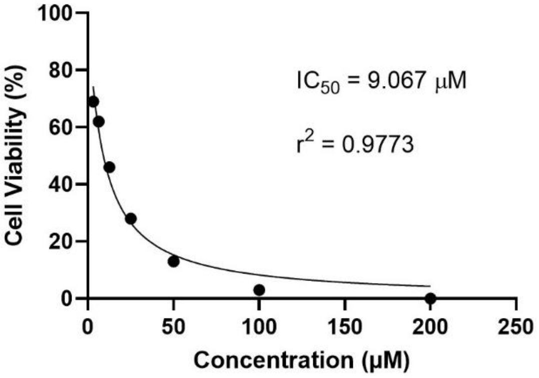


Compound 5o for A549

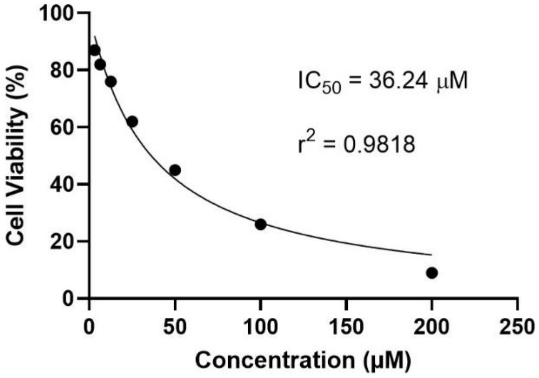


Compound 5o for BEAS-2B

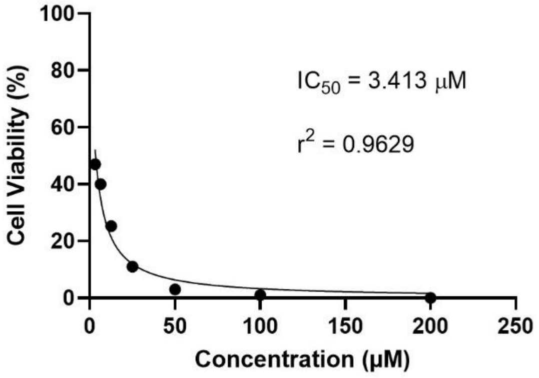


Compound 5p for A549

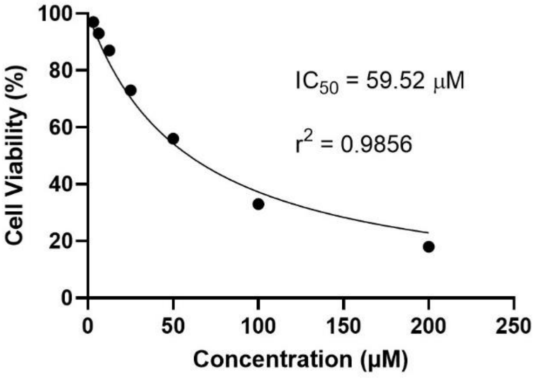


Compound 5p for BEAS-2B

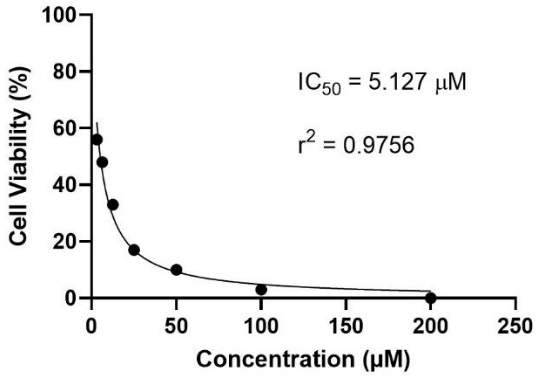


Compound 5q for A549

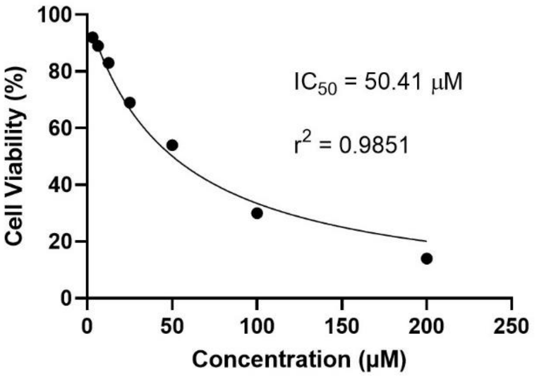


Compound 5q for BEAS-2B

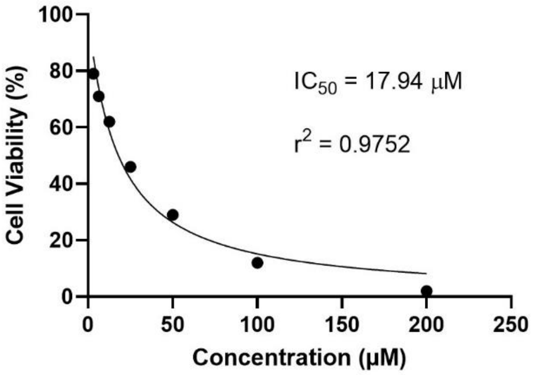


Compound 5r for A549

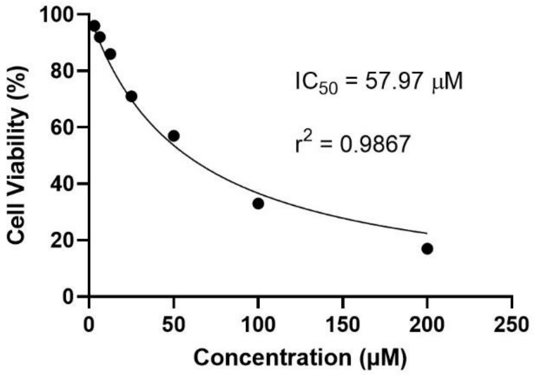


Compound 5r for BEAS-2B

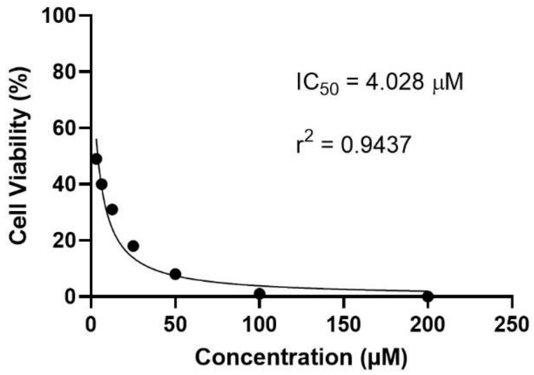


Sorafenib for A549

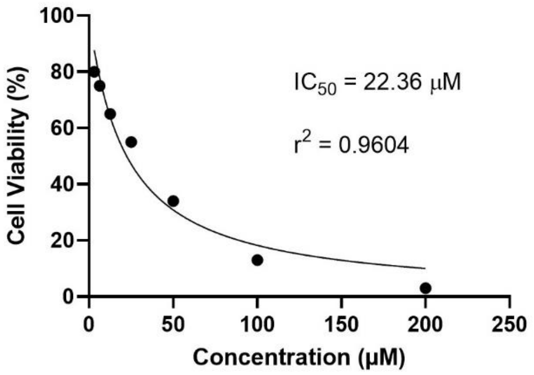


Sorafenib for BEAS-2B
